# PRKCSH contributes to TNFSF resistance by extending IGF1R half-life and activation in lung cancer

**DOI:** 10.1038/s12276-023-01147-1

**Published:** 2024-01-10

**Authors:** Gu-Choul Shin, Hyeong Min Lee, Nayeon Kim, Sang-Uk Seo, Kwang Pyo Kim, Kyun-Hwan Kim

**Affiliations:** 1 Department of Precision Medicine, School of Medicine, Sungkyunkwan University, Suwon, 16419 Republic of Korea; 2 Department of Microbiology, College of Medicine, The Catholic University of Korea, Seoul, 06591 Republic of Korea; 3 Department of Applied Chemistry, Institute of Natural Science, Global Center for Pharmaceutical Ingredient Materials, Kyung Hee University, Yongin, 446-701 Republic of Korea; 4 Department of Biomedical Science and Technology, Kyung Hee Medical Science Research Institute, Kyung Hee University, Seoul, 02453 Republic of Korea

**Keywords:** Non-small-cell lung cancer, Tumour biomarkers, Cancer therapeutic resistance

## Abstract

Tumor necrosis factor superfamily (TNFSF) resistance contributes to the development and progression of tumors and resistance to various cancer therapies. Tumor-intrinsic alterations involved in the adaptation to the TNFSF response remain largely unknown. Here, we demonstrate that protein kinase C substrate 80K-H (PRKCSH) abundance in lung cancers boosts oncogenic IGF1R activation, leading to TNFSF resistance. PRKCSH abundance is correlated with IGF1R upregulation in lung cancer tissues. Specifically, PRKCSH interacts with IGF1R and extends its half-life. The PRKCSH-IGF1R axis in tumor cells impairs caspase-8 activation, increases Mcl-1 expression, and inhibits caspase-9, leading to an imbalance between cell death and survival. PRKCSH deficiency augmented the antitumor effects of natural killer (NK) cells, representative TNFSF effector cells, in a tumor xenograft IL-2Rg-deficient NOD/SCID (NIG) mouse model. Our data suggest that PRKCSH plays a critical role in TNFSF resistance and may be a potential target to improve the efficacy of NK cell-based cancer therapy.

## PRKCSH: a potential game-changer in lung cancer resistance

Cancer cells often develop resistance to treatments that stimulate the natural cell death process, including immune therapies that initiate a group of proteins known as tumour necrosis factors (TNFs - proteins that can cause cell death). Researchers at Sungkyunkwan University in South Korea have discovered that a protein named PRKCSH is vital in assisting lung cancer cells to resist TNFs. Through a sequence of lab tests, they found that PRKCSH extends the lifespan of another protein, IGF1R, which subsequently prevents the activation of proteins that start cell death. When PRKCSH was reduced, cancer cells became more susceptible to TNFs and were more effectively eliminated by immune cells known as natural killer cells. These results suggest that focusing on PRKCSH could help overcome resistance to TNF-based cancer treatments. “This summary was initially drafted using artificial intelligence, then revised and fact-checked by the author.” not present in xml.

## Introduction

Tumor necrosis factor superfamily (TNFSF) resistance promotes tumor development and incapacitates the antitumor immune response^1–4^. Although several TNFSFs, including FasL, TNF-α, and TNF-related apoptosis-inducing ligand (TRAIL), have been identified as emerging cancer immunotherapeutic targets ^5–7^, most cancers are adapted to the TNFSF response, leading to an imbalance between cell death and survival; this renders them intractable, even against various cancer therapies such as immune checkpoint blockades and chemotherapeutics^8,9^. Sensitivity to the TNFSF response was initially thought to be a result of preferential expression of the individual TNFSF receptors in tumor cells^10^. However, TNFSF sensitivity in tumor cells was found to be more complex and is regulated at various levels of intracellular signaling^11–14^. Thus, an in-depth study of the common factors involved in tumor-intrinsic alterations is critical for developing novel approaches to restore sensitivity to the TNFSF response.

Natural killer (NK) cells contribute to spontaneous antitumor responses and are recognized as potent effector cells in cancer therapy^15^. Autologous or allogeneic NK cells are deemed safer effector cells in targeted cancer cell therapy than T cells because they preferentially target tumor cells without the need for prior sensitization or knowledge of specific tumor antigens^16–18^. NK cells exert their cytotoxic activity by inducing TNFSF expression^3,15,19^. Additionally, TNFSF is important for T and NK cell-mediated cytotoxicity to antigen-negative tumor cells^3,15,19^. Cytotoxic T-cell and NK cell evasion by tumor cells is primarily dependent on TNFSF resistance but occurs independently of perforin-mediated killing^20,21^. Although promising antitumor effects of NK cells have been reported, the underlying cellular mechanisms by which tumor cells adapt to NK cell-mediated antitumor immunity are still unclear.

Protein kinase C substrate 80K-H (PRKCSH), the glucosidase II beta subunit, is localized in the endoplasmic reticulum (ER) lumen, where it forms a complex with the glucosidase II alpha subunit and contributes to the folding and quality control of glycoproteins^22,23^. It also contributes to breast cancer progression and tumor resistance to EGFR inhibitors in non-small cell lung cancer^24,25^. Furthermore, we previously reported that PRKCSH may regulate the tumorigenesis of hepatocellular carcinoma by enhancing the activity of IRE1, a representative sensor of the ER stress response^26^. However, the key role of PRKCSH in tumor resistance against TNFSF- and NK cell-mediated cytotoxicity has not yet been investigated. Here, we demonstrate that PRKCSH inhibits the antitumor response by TNFSF members by affecting NK cell-mediated cytotoxicity.

## Materials and methods

### Cell lines and reagents

NK-92 (CRL-2407) human natural killer cells and non-small cell lung cancer cells, including A549 (CCL-185), H292 (CRL-1848), H1299 (CRL-5803), and H460 (HTB-177) cells, were obtained from the American Type Culture Collection (ATCC). Huh-7 (60104) and HepG2 (88065) hepatoma cells were purchased from the Korean Cell Line Bank. HCT116 (p53 null) human colon cancer cells were kindly provided by Dr. Y. H. Lee (Konkuk University, Republic of Korea). NK-92 cells were cultured in alpha-MEM (Welgene, LM008-02) supplemented with 10% fetal bovine serum, 10% horse serum, and 200 ng/mL human interleukin-2 (hIL-2), according to ATCC guidelines. Other cells were cultured in Dulbecco’s modified Eagle’s medium (DMEM) (Welgene, LM001-05) supplemented with 10% fetal bovine serum and 100 U/mL penicillin and streptomycin at 37 °C in a 5% CO_2_ incubator. All cells were maintained with a mycoplasma removal agent (MP Biomedicals, 3050044) and tested negative for mycoplasma using the Lonza Mycoplasma Detection Kit (Lonza Mycoalert, LT07-318) before experimental use.

For the neutralization assay for each receptor, the following antibodies were used: Iso-type control (R&D Systems, MAB002), TNF-α (R&D Systems, MAB610), and TRAIL antibody (R&D Systems, MAB375). For immunoblotting, following antibodies were used: TNF-R1 (R&D Systems, MAB225), TRAIL-R2 (Cell Signaling Technology, 8074), PRKCSH (Santacruz biotechnology, sc-46685), ACTB (Sigma‒Aldrich, A5441), Cas8 (Cell Signaling Technology, 9746; Proteintech, 13423-1-AP), Cas9 (Cell Signaling Technology, 9502), PARP1 (Cell Signaling Technology, 9542), p53 (Santacruz biotechnology, sc-7944), poly-ubiquitin (Enzo, PW0150), K63 ubiquitin (Cell Signaling Technology, 12930), K48 ubiquitin (Cell Signaling Technology, 8081), phospho-Cas8 (S347) (GeneTex, GTX55080), Mcl-1 (Santacruz biotechnology, sc-819), phospho-IGF1R (Cell Signaling Technology, 3918), IGF1R (Cell Signaling Technology, 9750), phospho-EGFR (Cell Signaling Technology, 3777), EGFR (Cell Signaling Technology, 4267), phospho-AKT (R&D Systems, AF887), AKT (Cell Signaling Technology, 9272), phospho-ERK1/2 (Cell Signaling Technology, 9101), ERK1/2 (Cell Signaling Technology, 4695), FADD (Santacruz biotechnology, sc-5559), FLIP (AdipoGen life sciences, AG-20B-0056), cIAP2 (Santacruz biotechnology, sc-7944), IRE1α (Cell Signaling Technology, 3294), phospho-IRE1α (Novus Biologicals, NB100-2323), sXBP-1 (Cell Signaling Technology, 40435), HERPUD1 (OriGene, TA507019), and Flag M2 (Sigma‒Aldrich, F1804). The following antibodies were used for IP: PRKCSH (Proteintech, 12148-1-AP), Cas8 (Cell Signaling Technology, 9746), and IGF1R (Cell Signaling Technology, 9750). For immunohistochemistry, the following antibodies were used: PRKCSH (Santa Cruz Biotechnology, sc-46685), IGF1R (Cell Signaling Technology, 14534), cleaved PARP1 (Cell Signaling Technology, 5625), and NKG2D (Abcam, ab203353). To determine the expression of each receptor on the surface of cancer cells, the following antibodies were purchased from R&D Systems: phycoerythrin (PE)-conjugated human TRAIL-R2 (FAB6311P), PE-conjugated IGF1R, and PE-conjugated isotype control (IC002P) antibodies. The anti-mouse antibody was conjugated with horseradish peroxidase (Sigma‒Aldrich, SAB3701153), and the anti-rabbit antibody was conjugated with horseradish peroxidase (Sigma‒Aldrich, A9169).

Human TRAIL (ABfrontier, LF-P0404), human TNF-α (YBDY Biotechnology, REC110), 3-[4,5-dimethylthiazol-2-yl]-2,5-diphenyltetrazolium bromide (MTT) (Sigma‒Aldrich, M2128), Cas8 inhibitor z-IETD-FMK (BioVision, 1064), Cas9 inhibitor z-LEHD-FMK (BioVision, 1074), pan-caspase inhibitor zVAD-FMK (BioVision, 1140), tunicamycin (TM) (Enzo Life Science, BML-CC104), chloroquine diphosphate salt (CQ) (Sigma‒Aldrich, C6628), MG132 (Sigma‒Aldrich, M8699), and cycloheximide (Sigma‒Aldrich, 01810) were used.

### Generation of acquired TRAIL-resistant cells

Acquired TRAIL-resistant cells, H292R and HepG2R, were derived from H292 (H292S) and HepG2 (HepG2S) cells, respectively, via in vitro selection for TRAIL resistance. Cells were periodically treated with increasing concentrations of TRAIL for two months and selected for resistance to TRAIL-induced apoptosis. TRAIL-resistant cells were continuously exposed to 50 ng/mL TRAIL to maintain resistance.

### siRNA and lentiviral shRNA transduction

Small interfering RNA (siRNA) duplexes were synthesized by Bionics (Seoul, Republic of Korea). Two siRNAs targeting PRKCSH (siPRK #1, 5′-GGA AGA AGU CUC UGG AAG ATT-3′ and siPRK #2, 5′-GGA AGA AGA GGC UGA AGA ATT-3′), siMcl-1 (5′-CAT CGA ACC ATT AGC AGA AAG TAT CTT-3′), siIGF1R (5′-AAG GAT ATT GGG CTT TAC AAC CTG ATT-3′), and a universal control siRNA were used. These siRNAs were transiently transfected using Lipofectamine 2000 reagent (Thermo Fisher Scientific, 11668027) at 20 nM/well concentration according to the manufacturer’s instructions.

Lentiviral particles containing shPRKCSH-GFP (LPP-HSH106860-LVRU6GP) were obtained from GeneCopoeia. Lung adenocarcinoma A549 cells were transduced with lentivirus in the presence of 10 μg/mL polybrene (Santa Cruz Biotechnology, sc-134220) and selected with 500 ng/mL puromycin (Thermo Fisher Scientific, A1113803) for 2 weeks. Transduced cells were confirmed by GFP expression and PRKCSH knockdown.

### Plasmid and lentiviral overexpression

For transient overexpression of human Mcl-1 (NM_021960), human Mcl-1 plasmids were obtained from Addgene. Lentiviral particles containing the human IGF1R plasmid (LPP-L0202-Lv105) were obtained from GeneCopoeia for overexpression of human IGF1R (NM_000875.4. To map the interaction domain, PRKCSH plasmids (carboxyl-terminal flag-tagged full-length, ΔG2B, ΔS/G2B, ΔMRH, and MRH) were used as previously reported^26^.

To stably overexpress hIL2 (NM_000586.3), lentiviral particles containing the hIL2 plasmid (LPP-A0413-Lv105) were obtained from GeneCopoeia and used to construct stable human interleukin-2 (hIL2)-expressing NK-92 cells (hIL2-NK-92 cells).

### Generation and analysis of tumor 3D spheroids

For spheroid generation, cells were seeded at optimized densities between 1000–1500 cells/well in 96-well U-bottom low-attachment plates (Corning, CLS7007) using a multichannel pipette. Spheroid formation was initiated by centrifuging the plates at 1000 × *g* for 10 min. Plates were incubated under standard cell culture conditions. Spheroid images were captured using an inverted microscope (Carl Zeiss AxioImager M2 fluorescence microscope), and spheroid volume and GFP signal intensity were analyzed using NIH ImageJ software.

### Tumor cell killing assays of NK-92 cells

For 2D culture conditions, NK cell cytotoxicity against tumor cells was analyzed using an LDH release assay. Tumor cells were seeded, and on the next day, NK-92 cells were added at various ratios (1:2.5, 1:5, and 1:10; target cells: effector cells). After 4 h of coculture, an aliquot of 50 μL of medium was used in the LDH cytotoxicity assay using the LDH cytotoxicity assay kit (DoGenBio, DG-LDH500). The value of the corrected experimental LDH release was calculated by subtracting the value of spontaneous LDH release from effector cells at the corresponding dilutions. NK cell cytotoxicity was defined as follows: percentage of cytotoxicity = (experimental value − effector cells spontaneous control − target cells spontaneous control)/(target cell maximum control − target cells spontaneous control) × 100.

For 3D tumor spheroid culture conditions, NK cell cytotoxicity against tumor cells was analyzed using propidium iodide (PI)/RNase staining solution (Cell Signaling Technology, 4087). Tumor cells were seeded, and on the next day, NK-92 cells were added at various ratios (1:2.5, 1:5, and 1:10; target cells: effector cells). After 5 days of coculture, dead cells were measured by the ratio of GFP (live cells) to PI (dead cells) signal intensity and spheroid volume. Images were captured using an inverted microscope (Carl Zeiss AxioImager M2 fluorescence microscope), and the spheroid volume and GFP signal intensity were measured using NIH ImageJ software.

For the NK cell cytotoxicity neutralization assay, NK-92 cells were treated with antibodies against TRAIL and TNF-α for 1 h, and then these NK-92 cells were applied to treat tumor cells in 2D culture or 3D organoids. After coculture, LDH release assays for 2D cultured tumor cells or measurements of spheroid volume and GFP signal intensity for 3D tumor organoids were performed as described above.

### Analysis of cell viability and cell death

Cell viability was determined using a 3-[4,5-dimethylthiazol-2-yl]-2,5-diphenyltetrazolium bromide (MTT) assay. Cells were seeded in 96-well plates and treated with various concentrations of human TRAIL (20, 40, 60, 80, and 100 ng/mL) and TNF-α/CHX (0.5, 1, 2.5, and 5 ng/mL) for 24 h. In some experiments, caspase inhibitors (25 μM; z-VAD-FMK, z-IETD-FMK, or z-LEHD-FMK) were added 1 h before the addition of 100 ng/mL TRAIL. At the end of the experiment, the culture medium was removed, and 50 μg/mL MTT solution was added for 30 min. Absorbance was measured at 450 nm using a SpectraMAX microplate reader (Molecular Devices), and the percentage of viable cells was calculated relative to that of untreated control cells.

Tumor cell death was determined by Annexin V and PI staining using the Annexin A5 Apoptosis Detection Kit (BioLegend, 640914) according to the manufacturer’s instructions. After treatment with TRAIL or TNF-α, cells were harvested and stained with 5 μL of FITC-conjugated Annexin V in 500 μL ice-cold binding buffer and 10 μL of PI solution for 15 min. Stained cells were analyzed using a FACSCalibur flow cytometer (Becton Dickinson) and Flowing Software (University of Turku, Finland) or WinMDI2.8 software (Scripps Research Institute, USA).

### Immunoblot analysis

Proteins were extracted from the cells lysed using SDS lysis buffer (100 mM Tris-HCl, pH 6.8, 10% glycerol, and 1% SDS) supplemented with a protease inhibitor cocktail (Thermo Fisher Scientific, 78441). The protein concentration was determined using a BCA protein assay kit (Thermo Fisher Scientific, 23225). Cell lysates were boiled in 1× sample buffer (10 mM Tris–HCl pH 6.8, 1% SDS, 5% glycerol, 0.05% bromophenol blue, and 1% β-mercaptoethanol) for 5 min. Proteins were separated by SDS‒PAGE and electrotransferred to Immobilon-P membranes (Merck, IPVH00010). Signals were detected using a LAS-4000 Luminescent Image Analyzer (GE Healthcare Bio-Sciences), and their intensity was assessed by calculating the relative density of each band normalized to that of the β-actin (ACTB) band using Multi Gauge software (Fujifilm).

### Immunohistochemistry analysis in lung cancer tissues

Lung cancer tissue array slides, 75 cases of lung squamous cell carcinoma (HLug-Squ150CS-01), and 75 cases of lung adenocarcinoma (HLugA150CS02) containing adjacent normal tissues were purchased from US Biomax and used to determine the levels of PRKCSH and IGF1R expression by immunohistochemical analysis. Clinicopathological information is available from the manufacturer’s website (http://www.tissue-array.co.kr/). The slides were deparaffinized with xylene and dehydrated with ethanol. The slides were subjected to antigen retrieval and incubated with a blocking solution to prevent nonspecific antibody binding, followed by incubation with anti-PRKCSH and anti-IGF1R primary antibodies as recommended by the suppliers at 4 °C overnight. After counterstaining with hematoxylin QS (Vector Laboratories, H-3404), the sections were dehydrated and mounted. The staining intensity of each protein was measured using NIH ImageJ software with the IHC Profiler plugin (http://rsb.info.nih.gov/ij/). The staining intensities of PRKCSH and IGF1R are shown as inverted median pixel values (IMPVs).

### RNA isolation and qPCR

Total RNA was isolated using TRIzol reagent (Sigma‒Aldrich, T9424) and reverse transcribed using M-MLV reverse transcriptase (IntRon Biotechnology, 27032) and an oligo-dT primer according to the manufacturer’s instructions. Quantitative PCR was performed on a 7500 Real-Time PCR System (Applied Biosystems) with SYBR Green PCR Master Mix (Thermo Fisher Scientific, 4309155) following the manufacturer’s protocols. To investigate the expression levels of Mcl-1 short and long form mRNAs, semiquantitative PCR was performed on an XP Thermal Cycler System (BIOER Technology). All primer sequences are listed in Supplementary Table 1.

### Analysis of the expression of receptors on the cell surface by flow cytometry

The cell surface expression of TRAIL-R2 and IGF1R was analyzed. Live cells were stained with PE-conjugated human TRAIL-R2 or PE-conjugated IGF1R antibodies for 30 min on ice. Stained cells were analyzed using a FACSCalibur flow cytometer (Becton Dickinson) and Flowing Software (University of Turku, Finland) or WinMDI2.8 software (Scripps Research Institute, USA). Receptor expression on the cell surface is shown as the percentage of the mean fluorescence intensity of the control cells.

### Analysis of ubiquitination of Cas8

For IP under denaturing conditions, proteins were extracted from cells using SDS lysis buffer, and then the lysates were heated for 10 min at 95 °C; the protein concentration was determined using the BCA protein assay kit. Samples were then diluted 1:10 in regular IP buffer (50 mM Tris-HCl [pH 7.2], 10 mM NaCl, 1% NP-40 [Sigma‒Aldrich, NP40S], and protease inhibitor cocktail [Cell Signaling Technology, 5871]) before IP. The prediluted lysate was precleared with protein A agarose (Sigma‒Aldrich, 11134515001) at 4 °C for 1 h, incubated with anti-caspase 8 (1:50) or normal isotype antibodies (1:50) at 4 °C overnight, and then incubated with protein A agarose at 4 °C for 4 h. The agarose beads were washed three times with IP buffer and boiled in 1× sample buffer. Boiled samples were analyzed by SDS‒PAGE and immunoblotting with anti-poly-ubiquitin, K63 ubiquitin, or K48 ubiquitin antibodies.

### Analysis of phosphorylation of receptor tyrosine kinases using antibody array

A549-shCon and A549-shPRK cells were treated with 100 ng/mL TRAIL or 10 ng/mL TNF-α for 4 h, and the phosphorylation of receptor tyrosine kinases was examined using a human phospho-RTK array kit (R&D Systems, ARY001B) according to the manufacturer’s instructions. Array signals were detected using an LAS-4000 Luminescent Image Analyzer, and their intensity was assessed by measuring the relative density of each spot using Multi Gauge software.

### Protein‒protein interaction assay

Nontransfected cells or cells transiently transfected with individual constructs were lysed using IP buffer containing a protease inhibitor cocktail. The lysate was precleared with protein A agarose at 4 °C for 1 h and incubated with anti-PRKCSH (1:50), anti-IGF1R (1:50), anti-Flag M2 (1:50), or corresponding normal isotype antibodies (1:50) at 4 °C overnight and then with protein A agarose at 4 °C for 4 h. The agarose beads were washed three times with lysis buffer and boiled in 1× sample buffer. The boiled samples were subjected to immunoblot analysis.

### Omics data analysis using public datasets

To analyze the differential expression of *PRKCSH* or *IGF1R* mRNA between cancer and normal tissues, the gene expression profiles of 1135 human lung cancer tissues (including 585 lung adenocarcinoma (LUAD) and 550 lung squamous cell carcinoma (LUSC) tissues) and 396 normal tissues were obtained from The Cancer Genome Atlas (TCGA) and Genotype-Tissue Expression (GTEX) databases using Xena Browser (https://xenabrowser.net). Additionally, *PRKCSH* and *IGF1R* mRNA expression in other cancer types and at different tumor stages of lung cancer was verified using the Gene Expression Profiling Interactive Analysis (GEPIA) online platform (https://gepia.cancer-pku.cn).

For survival analysis of patients with lung cancer, the prognostic values of *PRKCSH* or *IGF1R* mRNA levels were estimated using Kaplan–Meier plotter (https://www.kmplot.com). To evaluate the time to first progression, overall survival, or postprogression survival of lung cancer patients, patients were divided into high- and low-expression groups according to median mRNA levels. Hazard ratios (HRs) with 95% confidence intervals (CIs) and log-rank *P* values were calculated. Log-rank *P* values < 0.05 were considered to indicate significance. Univariate Cox regression analysis was conducted with adjustments for smoking status, clinical stage, chemotherapy, and lung cancer histology.

To analyze the functional gene pathways related to the expression of *PRKCSH* or *IGF1R* mRNA, coexpressed genes related to the expression of *PRKCSH* or *IGF1R* mRNA were obtained from the RNA-seq expression profiles of 503 LUAD (TCGA, PanCancer Atlas) and 487 LUSC (TCGA, PanCancer Atlas) samples from cBioPortal (https://www.cbioportal.org). Spearman’s correlation coefficient was used to assess the association between candidate gene expression and *PRKCSH* or *IGF1R* mRNA expression. Candidate genes significantly related to *PRKCSH* mRNA expression were selected from 8269 genes for LUAD and 8929 genes for LUSC. Candidate genes that were significantly related to *IGF1R* mRNA expression were selected from 8444 genes for LUAD and 8719 genes for LUSC. A Gene Ontology search was performed using G-Profiler to explore pathways in the Reactome and Kyoto Encyclopedia of Genes and Genomes (KEGG).

For GSEA, the coexpressed genes related to *PRKCSH* or *IGF1R* mRNA expression were analyzed and visualized using GSEA software (version 4.1.0, http://www.broadinstitute.org/gsea). NES and FDR were calculated for comparison.

### Xenograft tumor model for analysis of NK cell-mediated therapeutic effect

All animal experiments were performed in accordance with the guidelines of the internationally accepted principles for the care and use of laboratory animals and were covered by a personal license for GHBIO Genes to Health Biotechnology (AE2022-08 and AE2022-88). All mice were maintained in individually ventilated cages (IVCs) located in a specific pathogen-free (SPF) room at a constant temperature of 22 ± 1 °C, humidity of 55 ± 10%, and a 12-hour light/dark cycle.

To evaluate NK cell-mediated antitumor effects in xenograft tumor models, IL-2Rg-deficient NOD/SCID (NIG) mice (GHBIO Genes to Health Biotechnology) were used. A549-shCon or shPRK cells (5 × 10^6^ cells per 100 μL Matrigel) were injected subcutaneously into the flanks of 6-week-old male NIG mice (*n* = 10 each). Two weeks after tumor inoculation, when the tumor volume reached 150 mm^3^, the mice were randomly assigned to two groups. Saline or 5 × 10^6^ hIL2-NK-92 cells were intravenously injected into the mice on Days 22, 27, and 34 after tumor cell inoculation. Body weight and tumor size were monitored every 3‒4 days, and tumor volume was calculated using the formula *V* = π/6 (*L* × *W*^2^), where *W* and *L* are the tumor width and length, respectively. Mice were sacrificed on Day 40, and the tumors were weighed and measured.

For histological evaluation, tumors were collected from mice and processed into 5 μm slices. The paraffin sections were stained with anti-cleaved PARP1 and anti-NKG2D antibodies. IHC and data analysis were then performed as described previously.

### Statistical analysis

All experiments were repeated at least three times. Data are expressed as the mean ± SEM. Differences between the two groups were analyzed using Student’s *t* test. Immunohistochemical staining of the tissue array was performed using the chi-squared test. The survival times of patients with lung cancer were analyzed using Kaplan–Meier survival analyses with the log-rank test. Correlations between PRKCSH and IGF1R expression in lung cancer tissues were assessed using Pearson’s rank correlation coefficient. Statistical analyses were performed, and graphs were plotted using GraphPad Prism software (version 6, GraphPad Software, Inc.). *P* values < 0.05 were considered to indicate statistical significance.

## Results

### The expression level of PRKCSH is associated with TNFSF resistance in lung cancer

**Fig. 1:**
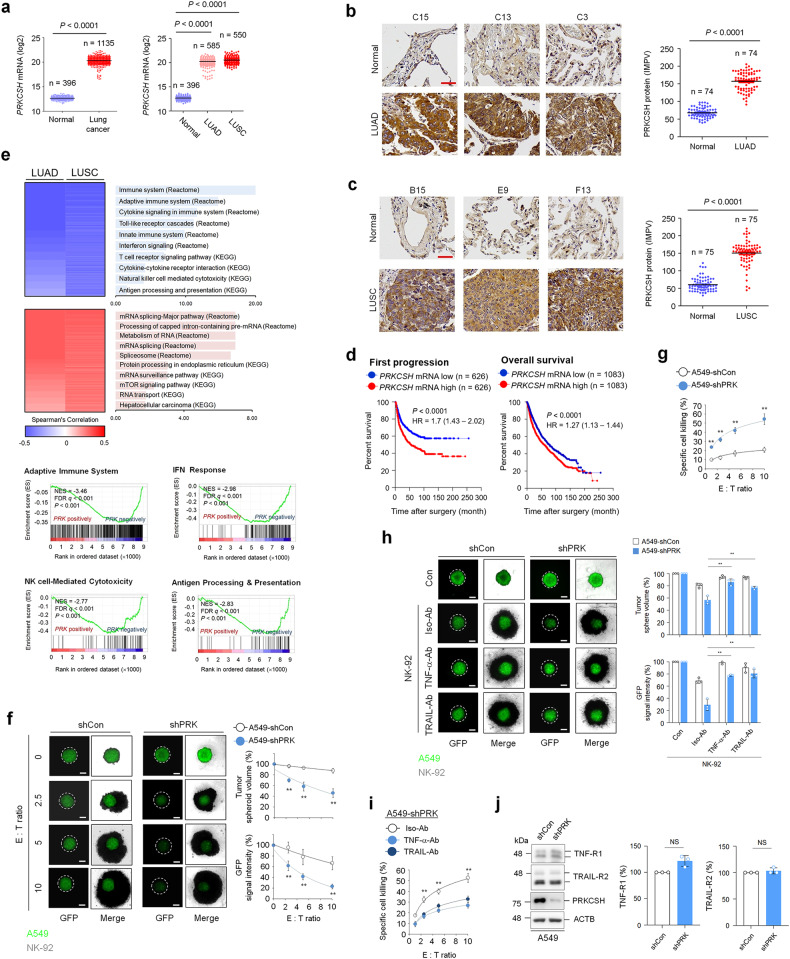
High expression of PRKCSH in lung cancer represses NK cell-mediated cytotoxicity. **a** Quantitative analysis of *PRKCSH* mRNA expression levels between normal (*n* = 396) and cancerous (*n* = 1135) lung tissues, including lung adenocarcinoma (LUAD) (*n* = 585) and lung squamous cell carcinoma (LUSC) (*n* = 550). The gene expression profiles were obtained from The Cancer Genome Atlas and Genotype-Tissue Expression databases. Data are presented as the mean ± the two-tailed Student determined SD. Statistical significance’s *t* test. **b** Representative images of immunohistochemical staining of PRKCSH in LUAD (*n* = 74 LUAD tissues; *n* = 74 adjacent tissues) (scale bar = 50 μm). Quantitative analysis of PRKCSH expression levels in paired clinical samples. **c** Representative images of immunohistochemical staining of PRKCSH in LUSC (*n* = 75 of LUSC tissues; n = 75 of adjacent tissues) (scale bar = 50 μm). Quantitative analysis of PRKCSH expression levels in paired clinical samples. **d** Kaplan‒Meier plot of the first progression rate or overall survival rate of patients with lung cancer stratified by *PRKCSH* mRNA expression level. Patients were divided into two groups: high *PRKCSH* mRNA expression vs. low *PRKCSH* mRNA expression. The significance of differences was determined by the two-sided log-rank test. **e** Two-dimensional hierarchical clustering shows top-ranked pathways in transcriptome analysis showing negatively coexpressed genes relative to *PRKCSH* mRNA expression (blue) and positively coexpressed genes (red) in LUAD and LUSC tissues. Gene-set enrichment analysis of negatively coexpressed genes with *PRKCSH* mRNA in LUSC tissues. NES, normalized enrichment score; FDR *q*, false discovery rate *q* value. **f** NK cell-A549 cell killing assay in tumor 3D spheroid culture (scale bar = 200 μm). The tumor organoids of A549-shCon and A549-shPRK cells were treated with NK-92 cells at the indicated E:T ratio for 5 days. The NK cell-mediated cytotoxicity against tumor organoids was analyzed by measuring spheroid volume and GFP signal intensity. Data are shown as the means ± SDs of three independent assays. The statistical significance of differences between two groups was determined with the two-tailed Student’s *t* test. **p < 0.01. **g** NK cell-A549 cell killing assay in tumor cell 2D culture. A549-shCon and A549-shPRK cells were seeded onto culture dishes following treatment of NK-92 cells at the indicated E:T ratio for 4 h. The NK cell-mediated cytotoxicity against tumor cells was measured using a lactate dehydrogenase release assay. **h** The inhibition assay of NK cell cytotoxicity with neutralizing antibodies against TNF-α or TRAIL in tumor 3D spheroid culture (scale bar = 200 μm). The tumor organoids of A549-shCon and A549-shPRK cells were treated with a complex of the neutralizing antibody and NK-92 cells for 5 days. The NK cell cytotoxicity against tumor organoids was analyzed by measuring spheroid volume and GFP signal intensity. **i** The inhib**i**tion assay of NK cell cytotoxicity with neutralizing antibodies against TNF-α or TRAIL in tumor cell 2D culture. A549-shCon and A549-shPRK cells were treated with the complex of the neutralizing antibody and NK-92 cells for 4 h. The NK cell-mediated cytotoxicity against tumor cells was measured by a lactate dehydrogenase release assay. **j** Immunoblot analysis of TNF-R1 and TRAIL-R2 expression in A549-shCon and A549-shPRK cells. Representative immunoblots and quantitative analysis of gene expression levels in A549-shCon and A549-shPRK cells are shown. ACTB was used as a loading control.

To first investigate the expression levels of PRKCSH in lung cancer, we analyzed *PRKCSH* mRNA expression using datasets from The Cancer Genome Atlas (TCGA) database. *PRKCSH* mRNA levels were higher in lung adenocarcinoma (LUAD) and lung squamous cell carcinoma (LUSC) tissues than in normal tissues (Fig. 1a). Consistent with public data, immunohistochemistry (IHC) using a tissue microarray (TMA) also showed an increased level of PRKCSH in LUAD and LUSC tissues compared to their paired normal tissues (Fig. 1b, c). Importantly, a follow-up analysis of patient survival using Kaplan‒Meier curves revealed that high expression of *PRKCSH* mRNA was correlated with poor first-progression survival and overall survival in patients with lung cancer (Fig. 1d). Subsequently, we analyzed *PRKCSH* mRNA expression levels at different stages of lung cancer. PRKCSH expression did not differ at different tumor stages in LUAD and LUSC (Supplementary Fig. 1). These results suggest that increased PRKCSH expression may be associated with the malignant transformation of lung cancer. To investigate PRKCSH-related functional events, we searched the TCGA database of lung cancer to identify genes correlated with *PRKCSH* mRNA expression. The Gene Ontology (GO) functional enrichment analysis and gene set enrichment analysis (GSEA) of genes negatively correlated with *PRKCSH* mRNA expression showed significant enrichment of the adaptive immune system and NK cell-mediated cytotoxicity, whereas the positively correlated genes were significantly enriched in the unfolded protein response (Fig. 1e and Supplementary Fig. 2).

Next, we investigated the potential role of PRKCSH in NK cell-mediated cytotoxicity. The tumor spheroid assay showed that PRKCSH depletion (shPRK) in A549 cells increased the sensitivity to NK-92 cell-mediated cytotoxicity compared to that in the control group (shCon) (Fig. 1f). This effect was also confirmed by a lactate dehydrogenase (LDH) release assay (Fig. 1g). We then investigated the relevant key regulators of NK cell-mediated cytotoxicity. The sensitization to NK-92 cell-mediated cytotoxicity mediated by PRKCSH depletion was significantly attenuated by antibodies against TNF-α and TRAIL (Fig. 1h, i). PRKCSH depletion did not cause the loss of TNF and TRAIL receptors (Fig. 1j). Our data indicated that PRKCSH may contribute to TNFSF resistance and lead to diminished NK cell-mediated cytotoxicity.

### PRKCSH contributes to the loss of TNFSF sensitivity

**Fig. 2:**
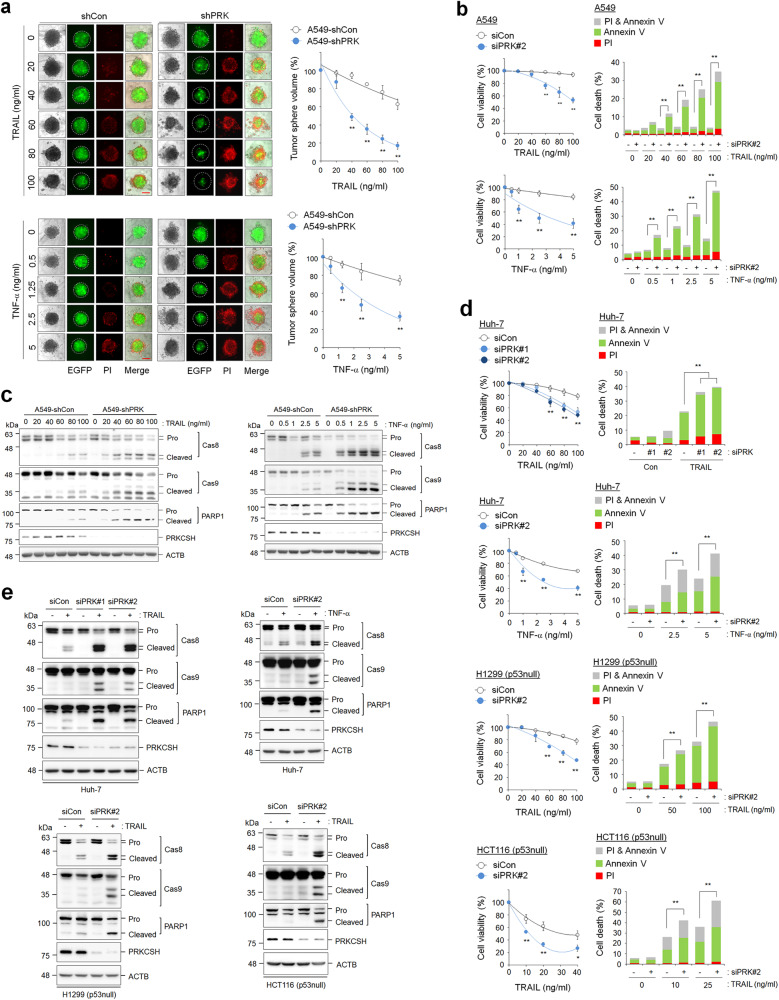
PRKCSH depletion increases the cytotoxicity of TNFSF against tumor cells. **a** TNFSF-mediated tumor cell killing assay in tumor 3D spheroid culture (scale bar = 200 μm). The tumor organoids of A549-shCon and A549-shPRK cells were treated with TRAIL or TNF-α at the indicated concentrations for 24 h. TNFSF-mediated cytotoxicity against tumor organoids was analyzed by measuring spheroid volume. Propidium iodide-stained cells indicate dead cells in tumor organoids. Data are shown as the means ± SDs of three independent assays. The statistical significance of differences between two groups was determined with the two-tailed Student’s *t* test. **b** TNFSF-mediated tumor cell killing assay in 2D cultured A549 cells. A549 cells transfected with PRKCSH siRNA (siPRK#2) or control siRNA (siCon) were treated with TRAIL or TNF-α at the indicated concentrations for 24 h. TNFSF-mediated cytotoxicity against tumor cells was analyzed by MTT assay (for cell viability) and propidium iodide and Annexin V staining assay (for cell death). **c** Immunoblot analysis of TNFSF-mediated activation of caspases in A549-shCon and A549-shPRK cells. Cells were treated with TRAIL or TNF-α at the indicated concentrations for 24 h, and the activation of caspases in cell lysates was analyzed by immunoblotting. **d** TNFSF-mediated tumor cell killing assay in other tumor cells. Hepatoma (Huh-7), lung cancer (H1299, p53 null), and colon cancer (HCT116, p53 null) cells were transfected with siRNAs to PRKCSH (siPRK #1 or #2) or control siRNA (siCon), followed by treatment with TRAIL or TNF-α at the indicated concentrations for 24 h. TNFSF-mediated cytotoxicity against tumor cells was analyzed by MTT assay (for cell viability) and propidium iodide and Annexin V staining assay (for cell death). **e** Immunoblot analysis of TNFSF-mediated activation of caspases in other tumor cells. Hepatoma (Huh-7), lung cancer (H1299, p53 null), and colon cancer (HCT116, p53 null) cells were transfected with siRNAs to PRKCSH (siPRK #1 or #2) or control siRNA (siCon), followed by treatment with TRAIL or TNF-α at the indicated concentrations for 24 h, and activation of caspases in cell lysates was analyzed by immunoblotting. **p < 0.01, *p < 0.05.

To examine the role of PRKCSH in TNFSF resistance, we determined the cytotoxicity of TNFSF when it was directly applied to PRKCSH-depleted tumor cells. Compared to the control group, the PRKCSH-depleted group of A549 cells showed dose-dependent reductions in tumor sphere formation in response to both TRAIL and TNF-α (Fig. 2a). Consistent with the tumor sphere formation results, PRKCSH depletion also accelerated TRAIL- and TNF-α-mediated cell death in A549 cells (Fig. 2b). In accordance with this, PRKCSH depletion increased the activation of caspase-8 and caspase-9, subsequently leading to the notable cleavage of PARP-1 upon TRAIL or TNF-α treatment; the control cells were used as the reference group (Fig. 2c and Supplementary Fig. 3a). These data indicate that PRKCSH indeed contributes to TNFSF resistance.

p53 activation is closely associated with TNFSF sensitivity^27^. However, similar to the effects on A549 cells harboring wild-type p53, PRKCSH depletion increased cell death upon TRAIL or TNF-α treatment in p53-defective cells, including Huh-7 (p53 mutant), H1299 (p53-null), and HCT116 (p53-null) cells (Fig. 2d and Supplementary Fig. 4a). PRKCSH depletion also increased the activation of caspase-8 and caspase-9 in p53-defective cells (Fig. 2e). These data suggest that PRKCSH-induced TNFSF resistance occurs via a p53-independent mechanism.

### PRKCSH is a key target for overcoming TNFSF resistance

**Fig. 3:**
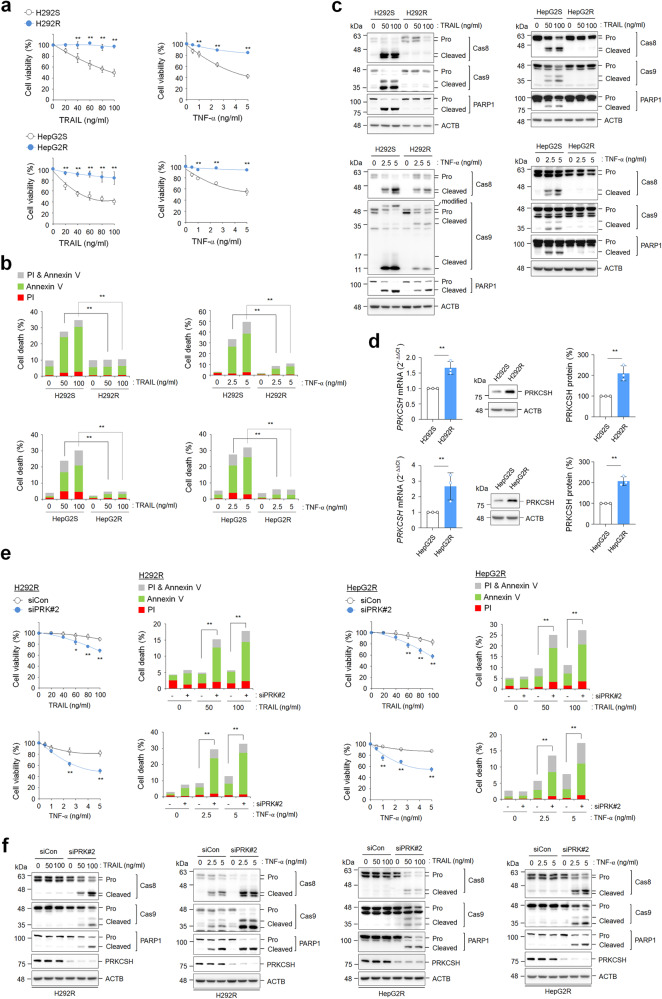
PRKCSH expression increased in cells with acquired TRAIL resistance, and its function was associated with cross-resistance to TNFSF. **a** Establishment of TRAIL-resistant H292 (H292R) and HepG2 (HepG2R) cells and identification of TRAIL sensitivity. Cell viability was assessed by MTT assay. Parent H292 (H292S) and H292R cells or parent HepG2 (HepG2S) and HepG2R cells were treated with TRAIL or TNF-α at the indicated concentrations for 24 h. Data are shown as the means ± SDs of three independent assays. The statistical significance of differences between two groups was determined with the two-tailed Student’s *t* test. **b** TNFSF-mediated tumor cell killing assay in H292S and H292R cells or HepG2S and HepG2R cells. Cells were exposed to TRAIL or TNF-α at the indicated concentrations for 24 h, and cytotoxicity was analyzed by propidium iodide and Annexin V staining assays. **c** Immunoblot analysis of TNFSF-mediated activation of caspases in H292S and H292R cells or HepG2S and HepG2R cells. Cells were treated with TRAIL or TNF-α at the indicated concentrations for 24 h, and the activation of caspases in cell lysates was analyzed by immunoblotting. **d** Analysis of PRKCSH mRNA and protein expression in H292S and H292R cells or HepG2S and HepG2R cells. Data are representative immunoblots and quantitative analysis of PRKCSH expression levels in each cell line. **e** TNFSF-mediated tumor cell killing assay in H292R or HepG2R cells. Cells transfected with siPRK#2 or siCon were treated with TRAIL or TNF-α at the indicated concentrations for 24 h. TNFSF-mediated cytotoxicity against tumor cells was analyzed by MTT assay (for cell viability) and propidium iodide and Annexin V staining assay (for cell death). **f** Immunoblot analysis of TNFSF-mediated activation of caspases in H292R or HepG2R cells. Cells transfected with siPRK#2 or siCon were treated with TRAIL or TNF-α at the indicated concentrations for 24 h, and the activation of caspases in cell lysates was analyzed by immunoblotting. **p < 0.01, *p < 0.05.

To further investigate whether PRKCSH is involved in acquired resistance to TNFSF-mediated cytotoxicity, we first established acquired TRAIL-resistant cells, denoted H292R (lung cancer cells) and HepG2R (hepatoma cells) from TRAIL-sensitive H292S and HepG2S cells, respectively, by long-term culture under TRAIL treatment. Both H292R and HepG2R cells were resistant to TRAIL-mediated cell death, as evidenced by the cell viability assay (Fig. 3a) and PI/annexin V staining (Fig. 3b). Interestingly, TRAIL-resistant cells were also resistant to TNF-α-mediated cell death (Fig. 3a, b). Consistent with the outcome of cell death, the activation of caspase-8 and caspase-9 was reduced in TRAIL-resistant cells (Fig. 3c). These results indicate that acquired TRAIL resistance in tumor cells induces cross-resistance to other TNFSF members.

Next, we investigated the potential role of PRKCSH in acquired TRAIL resistance. Published data on the expression of *PRKCSH* mRNA (GSE55859) revealed that *PRKCSH* mRNA expression is higher in H460R cells than in parental H460S cells (Supplementary Fig. 4b). *PRKCSH* mRNA and protein expression levels were significantly increased in H292R and HepG2R cells compared to parental H292S and HepG2S cells (Fig. 3d). We then determined whether PRKCSH depletion could attenuate the cell behavior changes related to acquired TRAIL resistance. PRKCSH depletion accelerated TRAIL- and TNF-α-mediated cell death in TRAIL-resistant cells (Fig. 3e). Accordingly, PRKCSH depletion in TRAIL-resistant cells increased the activation of caspase-8 and caspase-9 upon TRAIL or TNF-α treatment compared to that in control cells (Fig. 3f). These data suggest that PRKCSH may be a key factor in restoring acquired TNFSF resistance.

### PRKCSH regulates caspase-8 ubiquitination and phosphorylation

**Fig. 4:**
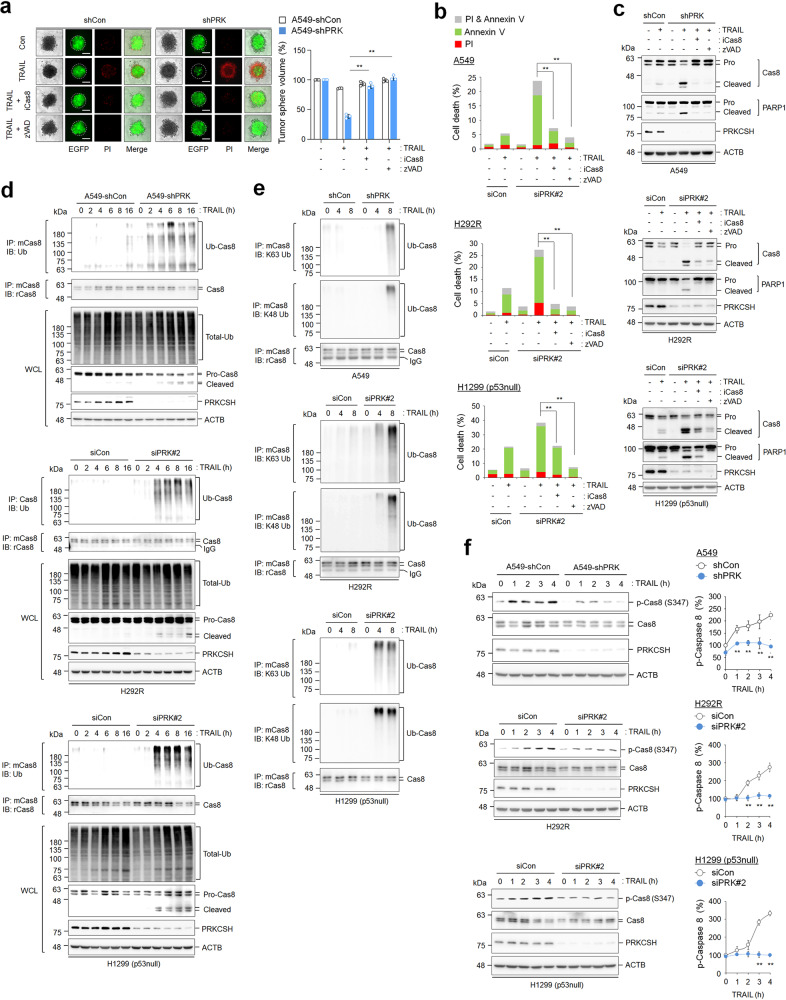
Role of PRKCSH in TNFSF-mediated caspase-8 activation, ubiquitination, and phosphorylation. **a** TNFSF-mediated tumor cell killing assay in tumor 3D spheroid culture (scale bar = 200 μm). The tumor organoids of A549-shCon and A549-shPRK cells were treated with caspase-8 inhibitor or pan-caspase inhibitor (zVAD-FMK) for 1 h, followed by treatment with 50 ng/mL TRAIL for 24 h. TNFSF-mediated cytotoxicity against tumor organoids was analyzed by measuring spheroid volume. Propidium iodide-stained cells indicate dead cells in tumor organoids. Data are shown as the means ± SDs of three independent assays. The statistical significance of differences between two groups was determined with the two-tailed Student’s *t* test. **b** TNFSF-mediated tumor cell killing assay in 2D cultured tumor cells. Lung cancer cells, including A549, H292R, and H1299 (p53 null) cells, were transfected with siPRK#2 or siCon, followed by treatment with 50 ng/mL TRAIL with or without caspase-8 inhibitor or zVAD-FMK for 24 h. TNFSF-mediated cytotoxicity against tumor cells was analyzed by propidium iodide and Annexin V staining assays. **c** Immunoblot analysis of TNFSF-mediated caspase-8 activation in lung cancer cells. Cells transfected with siPRK#2 or siCon were treated with TRAIL and/or caspase-8 inhibitor or zVAD-FMK for 24 h, and caspase-8 activation in cell lysates was analyzed by immunoblotting. **d** Immunoblot analysis of TRAIL-mediated ubiquitination of caspase-8 in A549-shCon and A549-shPRK cells or other lung cancer cells, including H292R and H1299 (p53 null) cells transfected with siPRK#2 or siCon. Cells were treated with 50 ng/mL TRAIL for the indicated times, and ubiquitinated caspase-8 in cell lysates was pulled down with anti-caspase-8 antibody, followed by immunoblot analysis with anti-ubiquitin antibody. **e** Immunoblot analysis of K63 or K48 ubiquitination of caspase-8 in A549-shCon and A549-shPRK cells or other lung cancer cells, including H292R and H1299 (p53 null) cells transfected with siPRK#2 or siCon. Cells were treated with 50 ng/mL TRAIL for the indicated times, and K63 or K48 ubiquitinated caspase-8 in cell lysates was pulled down with anti-caspase-8 antibody, followed by immunoblot analysis with anti-K63 or K48 ubiquitin antibody. **f** Immunoblot analysis of TNFSF-mediated caspase-8 phosphorylation in A549-shCon and A549-shPRK cells or other lung cancer cells, including H292R and H1299 (p53 null) cells transfected with siPRK#2 or siCon. Cells were treated with 50 ng/mL TRAIL for the indicated times, and caspase-8 phosphorylation in cell lysates was analyzed by immunoblotting. Data are representative immunoblots and quantitative analysis of caspase-8 phosphorylation levels in each cell line. **p < 0.01, *p < 0.05.

To investigate the molecular mechanism underlying PRKCSH-mediated TNFSF resistance, we determined the expression levels of pro- and antiapoptotic factors. PRKCSH depletion did not affect TNF-R1 and TRAIL-R2/DR5 expression (Fig. 1j, Supplementary Fig. 5a, b). In addition, the expression levels of caspase-8, FADD, FLIP, and cIAP2 were not affected by PRKCSH depletion (Supplementary Fig. 5c). As caspase-8 activity has been deemed a crucial feature of TNFSF sensitization, we investigated the effect of caspase-8 and pan-caspase (zVAD) inhibitors in PRKCSH-deficient cells. Tumor sphere size was significantly reduced by TRAIL treatment in PRKCSH-deficient cells, whereas it was restored by treatment with caspase-8 and pan-caspase inhibitors (Fig. 4a). Consistently, these inhibitors suppressed TRAIL-mediated apoptosis (Fig. 4b) and TRAIL-mediated caspase-8 activation in PRKCSH-deficient cells (Fig. 4c). These results indicate that TNFSF sensitization by PRKCSH depletion requires caspase-8 activation.

The polyubiquitination and aggregation of caspase-8 are needed for the regulation of apoptotic signaling^28^. Thus, we examined the involvement of PRKCSH in the ubiquitination of caspase-8 upon TRAIL treatment. PRKCSH depletion increased the ubiquitination of caspase-8 and thus increased the activation of caspase-8 compared to that in control cells (Fig. 4d, Supplementary Fig. 3b, c). PRKCSH depletion increased both TRAIL-mediated K48 and K63 ubiquitination of caspase-8 compared with that in control cells (Fig. 4e). These results indicate that PRKCSH regulates TNFSF resistance by inhibiting caspase-8 ubiquitination.

Caspase-8 phosphorylation inhibits its activation during spontaneous cell death and pro-apoptotic stimulation^29,30^. Thus, we investigated the levels of caspase-8 phosphorylation in PRKCSH-deficient cells. Caspase-8 phosphorylation was significantly increased by TRAIL treatment in lung cancer cells; however, it was almost completely inhibited by PRKCSH depletion (Fig. 4f). Taken together, these results suggest that the regulation of caspase-8 ubiquitination and phosphorylation by PRKCSH is associated with TNFSF resistance.

### PRKCSH inhibits caspase-9 activation through increased Mcl-1 expression

**Fig. 5:**
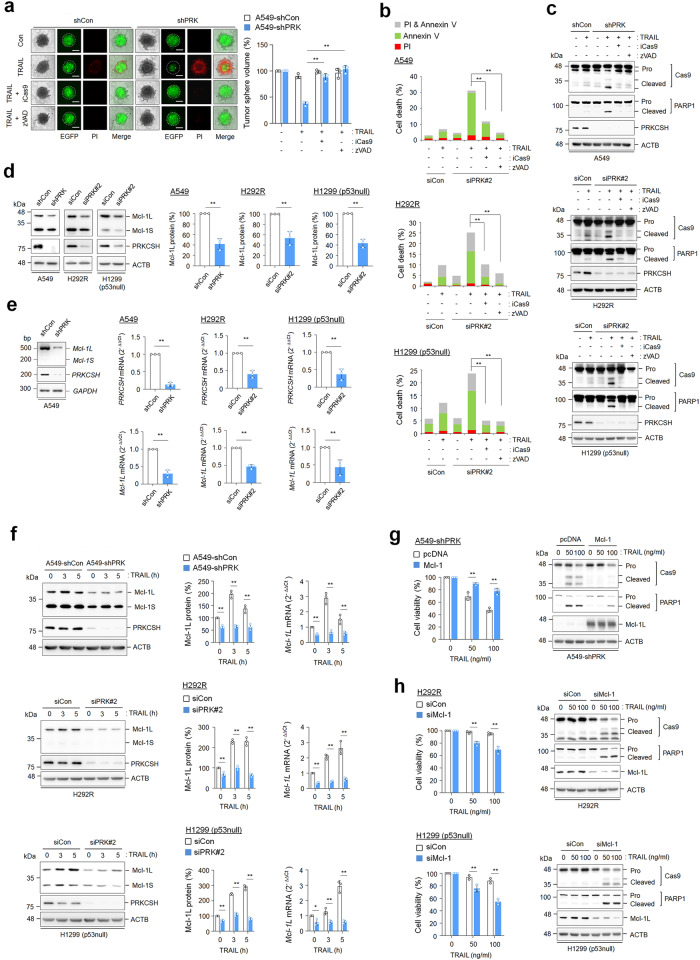
Role of PRKCSH in TNFSF-mediated caspase-9 activation and Mcl-1 expression. **a** TNFSF-mediated tumor cell killing assay in tumor 3D spheroid culture (scale bar = 200 μm). The tumor organoids of A549-shCon and A549-shPRK cells were treated with caspase-9 inhibitor or pan-caspase inhibitor (zVAD-FMK) for 1 h, followed by treatment with 50 ng/mL TRAIL for 24 h. TNFSF-mediated cytotoxicity against tumor organoids was analyzed by measuring spheroid volume. Propidium iodide-stained cells indicate dead cells in tumor organoids. Data are shown as the means ± SDs of three independent assays. The statistical significance of differences between two groups was determined with the two-tailed Student’s *t* test. **b** TNFSF-mediated tumor cell killing assay in 2D cultured tumor cells. Lung cancer cells, including A549, H292R, and H1299 (p53 null) cells, were transfected with siPRK#2 or siCon, followed by treatment with 50 ng/mL TRAIL with or without caspase-9 inhibitor or zVAD-FMK for 24 h. TNFSF-mediated cytotoxicity against tumor cells was analyzed by propidium iodide and Annexin V staining assays. **c** Immunoblot analysis of TNFSF-mediated caspase-9 activation in lung cancer cells. Cells transfected with siPRK#2 or siCon were treated with TRAIL and/or a caspase-9 inhibitor or zVAD-FMK for 24 h, and caspase-9 activation in cell lysates was analyzed by immunoblotting. **d** Immunoblot analysis of Mcl-1 protein expression in lung cancer cells. Cells were transfected with siPRK#2 or siCon for 48 h, and the expression levels of Mcl-1 long (Mcl-1L) and small form (Mcl-1S) in cell lysates were analyzed by immunoblotting. Data are representative immunoblots and quantitative analysis of Mcl-1 protein expression levels in each cell line. **e** Analysis of *Mcl-1* mRNA expression levels in lung cancer cells. The expression levels of *Mcl-1L* and *Mcl-1S* mRNA in A549-shCon and A549-shPRK cells or other lung cancer cells, including H292R and H1299 (p53 null) cells transfected with siPRK#2 or siCon, were analyzed by qPCR. Data are representative agarose gels and quantitative analysis of *Mcl-1* mRNA expression in each cell line. **f** Analysis of TNFSF-induced Mcl-1 protein and mRNA expression in lung cancer cells. Cells were treated with 50 ng/mL TRAIL for the indicated times, and the expression levels of Mcl-1L and Mcl-1S protein and mRNA were analyzed by immunoblotting and qPCR. Data are representative immunoblots and quantitative analysis of Mcl-1 protein and mRNA expression in each cell line. **g** The effect of Mcl-1 overexpression on TNFSF-mediated tumor cell killing in PRKCSH-deficient cells. A549-shPRK cells were transfected with Mcl-1 cDNA plasmid, followed by treatment with TRAIL at the indicated concentrations for 24 h. TNFSF-mediated cytotoxicity was analyzed by MTT assay, and caspase-9 activation was analyzed by immunoblotting. **h** The effect of Mcl-1 knockdown on TNFSF-mediated tumor cell killing in lung cancer cells. H292R and H1299 (p53 null) cells were transfected with siRNA to Mcl-1, followed by treatment with TRAIL at the indicated concentrations for 24 h. TNFSF-mediated cytotoxicity was analyzed by MTT assay, and caspase-9 activation was analyzed by immunoblotting. **p < 0.01, *p < 0.05.

To investigate the relevance of caspase-9 activity in TNFSF sensitization in PRKCSH-deficient cells, we investigated the effect of a caspase-9 inhibitor on tumor sphere formation. Caspase-9 inhibition restored the TRAIL-mediated reduction in tumor sphere size (Fig. 5a) and strongly suppressed TRAIL-mediated cell death and caspase-9 activation in PRKCSH-deficient cells (Fig. 5b, c). These results indicate that TNFSF sensitization in PRKCSH-deficient cells requires caspase-9 activation.

Mcl-1 is an antiapoptotic member of the Bcl-2 protein family that inhibits caspase-9 activation, and its expression is increased upon TRAIL treatment, which is involved in TNFSF resistance^31–33^. Thus, we examined whether PRKCSH altered Mcl-1 expression. Mcl-1 protein and mRNA levels were reduced in PRKCSH-deficient cells (Fig. 5d, e, Supplementary Fig. 6a, b). Upon TRAIL treatment, PRKCSH depletion decreased the protein and mRNA levels of both Mcl-1 long (Mcl-1L) and short (Mcl-1S) compared with those in control cells (Fig. 5f, Supplementary Fig. 6c, d). The overexpression of Mcl-1L in PRKCSH-deficient cells suppressed TRAIL-mediated cell death and caspase-9 activation (Fig. 5g), whereas Mcl-1 depletion in lung cancer cells enhanced TRAIL-mediated cell death and caspase-9 activation (Fig. 5h). These results suggest that Mcl-1 expression, promoted by PRKCSH, facilitates TNFSF resistance by inhibiting caspase-9 activation.

### PRKCSH potentiates IGF1R activation upon TNFSF treatment

**Fig. 6:**
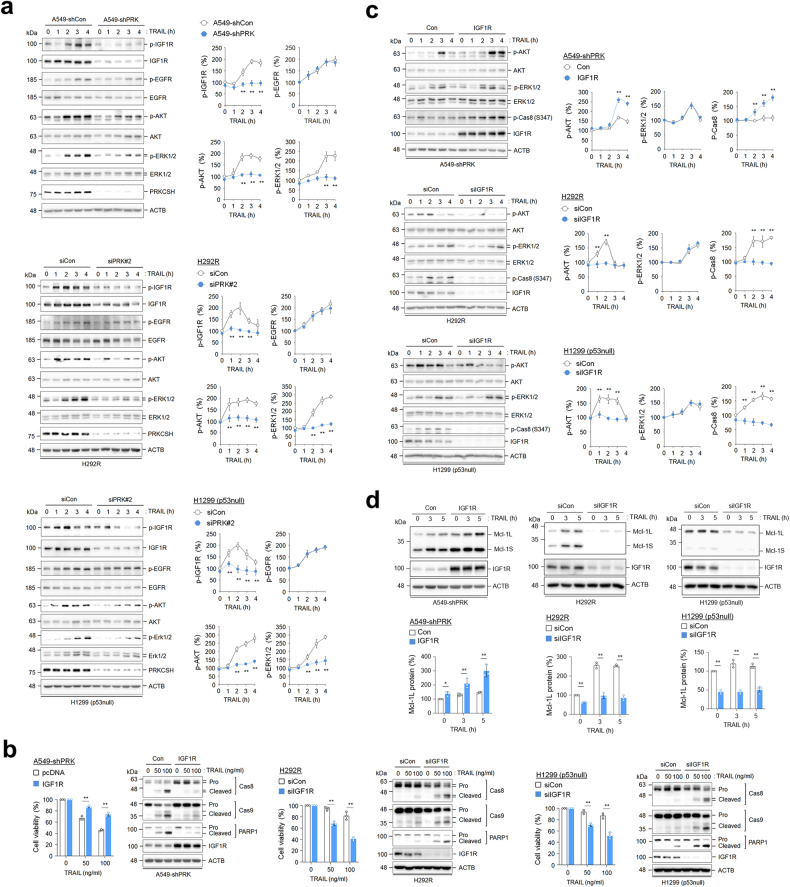
The effect of PRKCSH knockdown on the activation of the IGF1R signaling pathway. **a** Analysis of IGF1R and EGFR pathway activation in PRKCSH-deficient cells. A549-shCon and A549-shPRK cells or other lung cancer cells, including H292R and H1299 (p53 null) cells transfected with siPRK#2 or siCon, were treated with or without 50 ng/ml TRAIL for the indicated times. The phosphorylation of IGF1R, EGFR, AKT, and ERK1/2 in cell lysates was analyzed by immunoblotting. Representative immunoblots and quantitative analysis of phosphorylated protein levels in each cell line are shown. The quantitative data are shown as the means ± SDs of three independent assays. The statistical significance of differences between two groups was determined with the two-tailed Student’s *t* test. **b** The effect of IGF1R overexpression on TNFSF-mediated tumor cell killing in lung cancer cells. A549-shPRK cells were infected with lentivirus containing IGF1R cDNA plasmid, followed by treatment with TRAIL at the indicated concentrations for 24 h. TNFSF-mediated cytotoxicity was analyzed by MTT assay, and activation of caspases was analyzed by immunoblotting. H292R and H1299 (p53 null) cells were transfected with siRNA to IGF1R, followed by treatment with TRAIL at the indicated concentrations for 24 h. TNFSF-mediated cytotoxicity was analyzed by MTT assay, and activation of caspases was analyzed by immunoblotting. **c** The effect of IGF1R overexpression or knockdown on TNFSF-mediated phosphorylation of AKT and ERK1/2 in lung cancer cells. A549-shPRK cells were infected with lentivirus containing IGF1R cDNA plasmid, or H292R and H1299 (p53 null) cells were transfected with siRNA to IGF1R. Cells were treated with TRAIL for the indicated times, followed by determination of AKT and ERK1/2 phosphorylation by immunoblotting. Representative immunoblots and quantitative analysis of phosphorylated protein levels in each cell line are shown. **d** The effect of IGF1R overexpression or knockdown on TNFSF-mediated Mcl-1 expression in lung cancer cells. A549-shPRK cells were infected with lentivirus containing IGF1R cDNA plasmid, or H292R and H1299 (p53 null) cells were transfected with siRNA to IGF1R. Cells were treated with TRAIL for the indicated times, followed by determining the expression of Mcl-1L and Mcl-1S by immunoblotting. Representative immunoblots and quantitative analysis of Mcl-1L and Mcl-1S protein levels in each cell line are shown. **p < 0.01, *p < 0.05.

TNFSF can indirectly regulate the activation of receptor tyrosine kinase (RTK) families^34–36^. Thus, we investigated RTK activation by TNFSF in PRKCSH-deficient cells using an antibody array for phospho-RTK. IGF1R phosphorylation was markedly inhibited in PRKCSH-deficient cells upon TNFSF treatment (Supplementary Fig. 7). Immunoblotting results also showed that TRAIL-induced phosphorylation of IGF1R, AKT, and ERK1/2 was significantly reduced in PRKCSH-deficient cells compared with control cells, whereas the levels of phosphorylated EGFR were not significantly different between the two cell types (Fig. 6a). These results suggest that PRKCSH plays a role in the regulation of IGF1R activation.

We then investigated whether IGF1R activation is important for TNFSF resistance. IGF1R overexpression in PRKCSH-deficient cells reduced TRAIL-mediated cell death and caspase-8 and caspase-9 activation (Fig. 6b). In contrast, IGF1R depletion increased TRAIL-mediated cell death and caspase-8 and caspase-9 activation (Fig. 6b), revealing that PRKCSH-mediated IGF1R activation is involved in TNFSF resistance by inhibiting caspase-8 and caspase-9 activation. Ectopic expression of IGF1R significantly increased the levels of phosphorylated AKT and caspase-8 in PRKCSH-deficient cells (Fig. 6c), whereas IGF1R depletion decreased the levels of phosphorylated AKT and caspase-8 (Fig. 6c). Ectopic expression of IGF1R promoted both basal and TRAIL-induced Mcl-1L and Mcl-1S expression, whereas IGF1R depletion decreased Mcl-1L and Mcl-1S levels under both conditions (Fig. 6d). These results suggest that PRKCSH-mediated IGF1R activation is critical for enhancing caspase-8 phosphorylation and Mcl-1 expression.

### PRKCSH enhances the stability of IGF1R protein

**Fig. 7:**
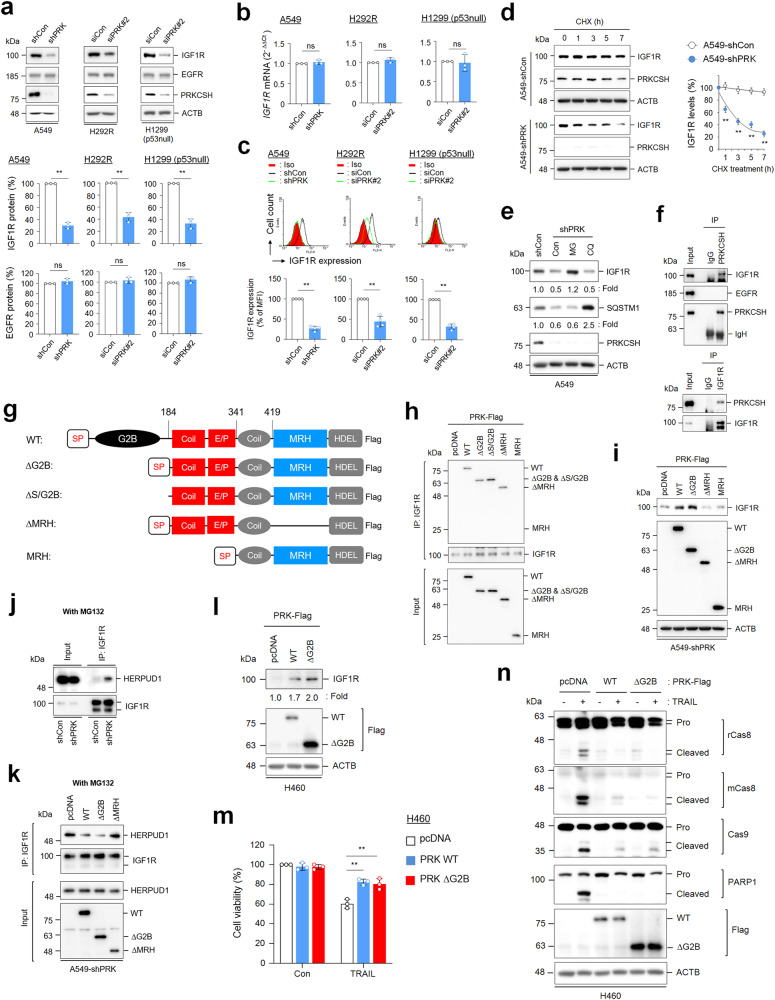
The role of PRKCSH in the regulation of IGF1R protein half-life and its functional domain. **a** Analysis of IGF1R and EGFR protein levels in PRKCSH-deficient cells. A549-shCon and A549-shPRK cells or other lung cancer cells, including H292R and H1299 (p53 null) transfected with siPRK#2 or siCon, were analyzed by immunoblotting using anti-IGF1R and EGFR antibodies. Representative immunoblots and quantitative analysis of IGF1R and EGFR protein levels in each cell line are shown. The quantitative data are shown as the means ± SDs of three independent assays. The statistical significance of differences between two groups was determined with the two-tailed Student’s *t* test. **b** Analysis of *IGF1R* mRNA expression levels in PRKCSH-deficient cells. The expression levels of *IGF1R* mRNA in A549-shCon and A549-shPRK cells or other lung cancer cells, including H292R and H1299 (p53 null) cells transfected with siPRK#2 or siCon, were analyzed by qPCR. Data are quantitative analysis of *IGF1R* mRNA expression in each cell line. **c** Analysis of IGF1R protein expression levels on the cell surface of PRKCSH-deficient cells. The expression of IGF1R protein on the cell surface of A549-shCon and A549-shPRK cells or other lung cancer cells, including H292R and H1299 (p53 null) cells transfected with siPRK#2 or siCon, was analyzed by flow cytometry. Representative flow cytometry data and quantitative analysis of IGF1R expression levels on the cell surface of each cell line are shown. **d** The effect of PRKCSH depletion on the IGF1R protein half-life in lung cancer cells. A549-shCon and A549-shPRK cells were treated with cycloheximide for the indicated times. The half-life of IGF1R protein in cell lysates was analyzed using immunoblotting. Representative immunoblots and quantitative analysis of IGF1R protein levels are shown. **e** The effect of proteasome or lysosome inhibitors on the regulation of IGF1R protein half-life in lung cancer cells. A549-shCon and A549-shPRK cells were treated with MG132 (proteasome inhibitor) or chloroquine (lysosome inhibitor) for 24 h. SQSTM1 was used as a lysosome inhibition control, and ACTB was used as a loading control. **f** Analysis of protein‒protein interactions among endogenous IGF1R, EGFR, and PRKCSH in lung cancer cells. The protein extracts from A549 cells were pulled down with anti-PRKCSH or anti-IGF1R antibodies, and the interacting proteins were identified by immunoblotting. **g** Scheme of the protein domains expressed in wild-type and mutant PRKCSH plasmids. WT, carboxyl-terminal Flag-tagged full-length PRKCSH; ΔG2B, deletion mutant of the G2B domain in WT; ΔS/G2B, deletion mutant of the signal peptide and G2B domain in WT; ΔMRH, deletion mutant of MRH in ΔG2B; MRH, deletion of the EP domain in ΔG2B. **h** Mapping domain of PRKCSH related to the interaction with IGF1R. A549 cells were transfected with WT PRKCSH and each PRKCSH deletion mutant plasmid. The protein extracts were pulled down with an anti-IGF1R antibody, and the interacting proteins were determined by immunoblotting with an anti-Flag antibody. **i** Mapping domain of PRKCSH associated with regulation of IGF1R protein half-life. A549-shPRK cells were transfected with WT and each deletion mutant of PRKCSH plasmids. IGF1R protein expression levels were determined by immunoblotting. **j** Analysis of the interaction between IGF1R and HERPUD1 in lung cancer cells. A549-shCon and A549-shPRK cells were treated with MG132 for 24 h. The protein extracts from each cell were pulled down with an anti-IGF1R antibody, and the interaction between IGF1R and HERPUDP1 protein was determined using immunoblotting. **k** The role of WT and each deletion mutant of PRKCSH in the inhibition of the interaction between IGF1R and HERPUD1. A549-shPRK cells were transfected with each plasmid, followed by treatment with MG132 for 24 h. The protein extracts from each cell were pulled down with an anti-IGF1R antibody, and the interaction between IGF1R and HERPUDP1 protein was determined using immunoblotting. **l** The effect of PRKCSH overexpression on IGF1R protein half-life. H460 lung cancer cells were transfected with WT or ΔG2B mutant plasmid. IGF1R protein expression levels were determined using immunoblotting. **m-n** The effect of PRKCSH overexpression on TNFSF-mediated tumor cell killing in H460 lung cancer cells. H460 cells were transfected with PRKCSH WT or ΔG2B mutant plasmid, followed by treatment with TRAIL for 24 h. TNFSF-mediated cytotoxicity was analyzed using MTT assay (**m**), and activation of caspases was analyzed using immunoblotting (**n**). **p < 0.01, *p < 0.05. NS, nonsignificant.

We investigated the relationship between the expression levels of PRKCSH and IGF1R. PRKCSH depletion reduced IGF1R protein levels but not EGFR protein levels (Fig. 7a and Supplementary Fig. 6e). However, there was no significant difference in *IGF1R* mRNA levels between PRKCSH-deficient and control cells (Fig. 7b and Supplementary Fig. 6e). Consistent with the effect of PRKCSH on IGF1R protein expression, the cell surface expression of IGF1R was also reduced by PRKCSH depletion (Fig. 7c). The half-life of IGF1R was significantly reduced in PRKCSH-deficient cells compared to control cells (Fig. 7d and Supplementary Fig. 8a). These data indicate that PRKCSH enhances the IGF1R half-life. PRKCSH depletion induces autophagy activation^37^; thus, we further investigated whether PRKCSH regulates IGF1R stability by regulating proteasome or lysosomal activity. Our data also showed that PRKCSH depletion promoted the degradation of the autolysosomal substrate SQSTM1 protein in lung cancer cells, and its level was increased via the lysosome inhibitor CQ. However, PRKCSH depletion-mediated degradation of the IGF1R protein was not increased by CQ but was recovered by the proteasome inhibitor MG132 (Fig. 7e and Supplementary Fig. 8b). These findings suggest that PRKCSH protects against the degradation of the IGF1R protein through the proteasome pathway.

Next, we investigated whether IGF1R stabilization was directly regulated by its interaction with PRKCSH. Endogenous IGF1R and PRKCSH interacted with each other, but EGFR and PRKCSH did not (Fig. 7f). Immunoprecipitation (IP) using deletion mutants of PRKCSH (Fig. 7g) showed that the internal coil and E/P domains (amino acids 184–341) of PRKCSH are needed for its interaction with IGF1R (Fig. 7h). Interestingly, a mutant lacking the ER localization signal peptide in PRKCSH also interacted with IGF1R, suggesting that the interaction between PRKCSH and IGF1R does not require ER components. We then determined the functional domain of PRKCSH needed to regulate IGF1R stabilization. Consistent with the role of endogenous PRKCSH, ectopic expression of PRKCSH also increased IGF1R protein levels in PRKCSH-deficient cells (Fig. 7i). Notably, a mutant lacking the MRH domain (ΔMRH) and only the MRH domain failed to increase the levels of IGF1R. These results indicated that these domains of PRKCSH (the Coil-E/P and MRH domains) are needed for binding to IGF1R and extending the IGF1R half-life, respectively. A previous study demonstrated that PRKCSH protects the PKD2 protein against HERPUD1-mediated ER-associated degradation (ERAD)^23^. Thus, we investigated whether PRKCSH competes with HERPUD1 to regulate IGF1R half-life. PRKCSH depletion promoted the interaction between HERPUD1 and IGF1R in lung cancer cells (Fig. 7j). Furthermore, the interaction was suppressed by the ectopic expression of PRKCSH-WT and a mutant lacking the G2B domain (ΔG2B) but not by the ΔMRH mutant (Fig. 7k). These results indicate that the MRH domain of PRKCSH is critical for inhibiting the interaction between HERPUD1 and IGF1R.

Subsequently, the potential role of PRKCSH in TNFSF resistance was confirmed by ectopic overexpression of PRKCSH-WT or ΔG2B in PRKCSH-deficient cancer cells. Supplementation of PRKCSH-deficient cells with PRKCSH-WT or ΔG2B significantly restored TRAIL-mediated cell death (Supplementary Fig. 8c) and suppressed TRAIL-induced caspase-8 and caspase-9 activation (Supplementary Fig. 8d). To confirm these effects, we investigated the effects of the ectopic overexpression of PRKCSH-WT or ΔG2B in the TNFSF-sensitive cancer cell line H460. Consistent with the results in PRKCSH-deficient cancer cells (Fig. 7i), overexpression of PRKCSH-WT or ΔG2B in H460 cells increased IGF1R protein levels (Fig. 7l). PRKCSH overexpression restored TRAIL-mediated cell death (Fig. 7m) and suppressed TRAIL-induced caspase activation (Fig. 7n). Collectively, these data suggested that PRKCSH enhances TNFSF resistance through IGF1R stabilization.

Previously, we reported that PRKCSH promotes IRE1α activation in hepatocellular carcinoma^26^. Thus, we investigated whether the PRKCSH-mediated extension of the IGF1R half-life is linked to IRE1α activation in lung cancer. PRKCSH depletion also inhibited ER stress-induced IRE1α activation and XBP-1 splicing in lung cancer cells (Supplementary Fig. 8e). However, this inhibition was not reversed via ectopic expression of IGF1R in PRKCSH-deficient cancer cells. Therefore, our findings suggest that the PRKCSH-mediated regulation of IGF1R half-life is not linked to the regulation of the IRE1α pathway.

### PRKCSH-IGF1R abundance attenuates NK cell-mediated antitumor effects

**Fig. 8:**
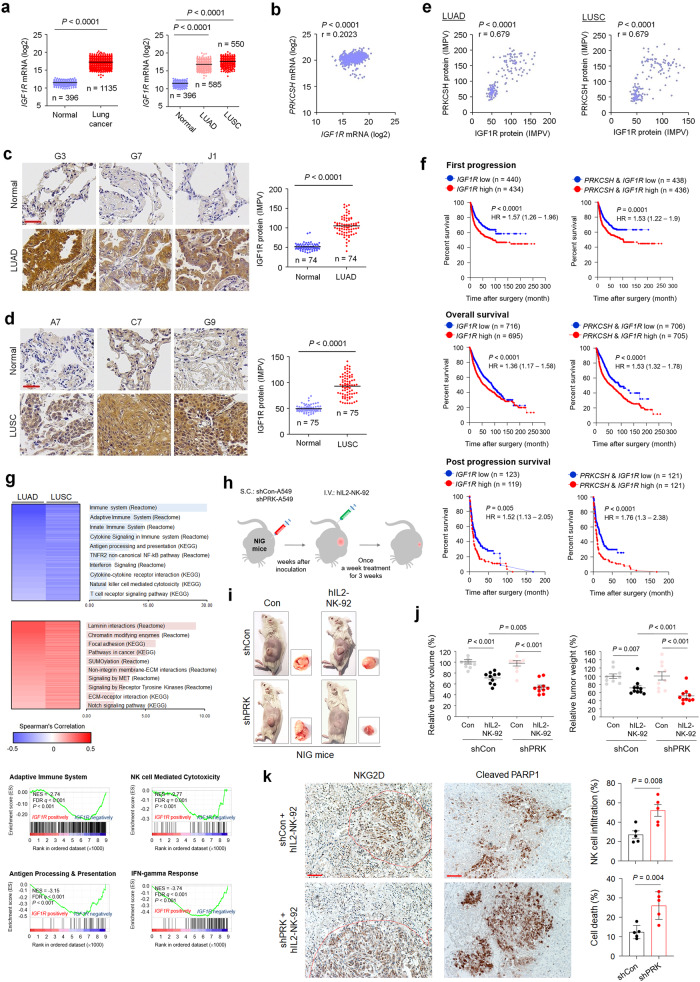
The impact of PRKCSH protein on increasing IGF1R protein levels in lung cancer tissues and the NK cell-mediated antitumor effect in vivo. **a** Quantitative analysis of *IGF1R* mRNA expression levels between normal (*n* = 396) and cancerous (*n* = 1135) lung tissues, including lung adenocarcinoma (LUAD) (*n* = 585) and lung squamous cell carcinoma (LUSC) (*n* = 550). The gene expression profiles were obtained from The Cancer Genome Atlas and Genotype-Tissue Expression databases. Data are presented as the mean ± SD. Statistical significance was determined by the two-tailed Student’s *t* test. **b** Representative immunohistochemical staining of IGF1R protein in LUAD (*n* = 74 of LUAD tissues; *n* = 74 of adjacent tissues) (scale bar = 50 μm). Quantitative analysis of IGF1R expression levels in paired clinical samples. **c** Representative immunohistochemical staining of IGF1R protein in LUSC (*n* = 75 of LUSC tissues; *n* = 75 of adjacent tissues) (scale bar = 50 μm). Quantitative analysis of IGF1R expression levels in paired clinical samples. **d** Analysis of the correlation between *PRKCSH* and *IGF1R* mRNA levels in lung cancer tissues using the gene expression profiles obtained from The Cancer Genome Atlas database. Correlation analysis was performed using Pearson’s rank correlation coefficient. **e** Analysis of the correlation between PRKCSH and IGF1R protein levels in lung cancer tissues using the immunohistochemical data determined in LUAD and LUSC tissues. **f** Kaplan‒Meier plot of the first progression rate, overall survival rate, or post progression survival rate of patients with lung cancer stratified by *IGF1R* mRNA expression level. Patients were divided into two groups: high *IGF1R* mRNA expression vs. low *IGF1R* mRNA expression. Statistical differences were determined by the two-sided log-rank test. **g** Two-dimensional hierarchical clustering shows top-ranked pathways in transcriptome analysis showing negatively coexpressed genes relative to *IGF1R* mRNA expression (blue) and positively coexpressed genes (red) in LUAD and LUSC tissues. Gene-set enrichment analysis of coexpressed genes with *IGF1R* mRNA in LUSC tissues. NES, normalized enrichment score; FDR *q*, false discovery rate *q* value. **h**‒**j** The effect of PRKCSH knockdown on the NK cell-mediated antitumor effect using the tumor-bearing IL-2Rg-deficient NOD/SCID (NIG) mouse model (each group, *n* = 10). A549-shCon and A549-shPRK cells were inoculated subcutaneously into the mice, and human IL2-NK-92 cells were injected intravenously into the mice at 3 weeks after inoculation (**h**). Tumor volume and weight were measured on Day 40 after inoculation. Representative tumor-bearing mice and enucleated tumors (**i**) and quantitative analysis of tumor volume and weight (**j**) are shown. **k** Representative immunohistochemical staining of cell death (cleaved PARP1) and NK cell infiltration (NKG2D) in tumor tissues of xenograft mice (each group, *n* = 5) (scale bar = 50 μm). Quantitative analysis of cell death and NK cell infiltration levels.

Previously, we reported that *PRKCSH* mRNA levels are upregulated in various cancers, such as glioblastoma multiforme (GBM), esophageal carcinoma (ESCA), lymphoid neoplasm- diffuse large B-cell lymphoma (DLBC), thymoma (THYM), liver hepatocellular carcinoma (LIHC), pancreatic adenocarcinoma (PAAD), stomach adenocarcinoma (STAD), skin cutaneous melanoma (SKCM) and lung cancer^26^. To investigate the relationship between the expression levels of PRKCSH and IGF1R in cancer patient tissues, we first analyzed *IGF1R* mRNA expression in various cancer tissues using the TCGA database. Unlike the pattern of *PRKCSH* mRNA expression in various cancer types, *IGF1R* mRNA levels were upregulated only in LUAD, LUSC, and THYM tissues compared normal tissues (Fig. 8a and Supplementary Fig. 9a), whereas its expression was downregulated in DLBC and was not different in GBM, ESCA, LIHC, PAAD, STAD, and SKCM compared to the corresponding normal tissues (Supplementary Fig. 9a). Subsequently, we analyzed *IGF1R* mRNA expression levels at different stages of lung cancer. IGF1R expression did not differ at different tumor stages in LUAD and LUSC (Supplementary Fig. 9b). These results suggest that increased IGF1R expression may be more important for malignant transformation, particularly in lung cancer, than in other cancer types. Thus, these findings indicate that *PRKCSH* mRNA expression is not associated with *IGF1R* mRNA expression in other cancer types. Indeed, *PRKCSH* mRNA levels are not correlated with *IGF1R* mRNA levels in lung cancer (Fig. 8b). Next, we investigated the clinical relevance of IGF1R expression using TMA to determine PRKCSH protein expression. The IGF1R protein was significantly upregulated in LUAD and LUSC tissues compared to adjacent normal tissues (Fig. 8c, d). Unlike the relationship between *PRKCSH* and *IGF1R* mRNA levels, PRKCSH protein levels were positively correlated with IGF1R protein levels in LUAD and LUSC (Fig. 8e). These results indicated that the relationship between PRKCSH and IGF1R proteins has in vivo relevance in lung cancer tissues.

Kaplan‒Meier curves demonstrated that high *IGF1R* mRNA expression was correlated with poor first progression survival and overall survival but not postprogression survival in patients with lung cancer (Fig. 8f). Notably, patients with high expression of both *PRKCSH* and *IGF1R* mRNA showed significantly lower survival rates than those with low expression of both *PRKCSH* mRNA and *IGF1R* mRNA (Fig. 8f). These results suggest that the overexpression of PRKCSH and IGF1R promotes the onset of lung cancer and correlates with an unfavorable prognosis in lung cancer patients.

To investigate IGF1R-related functional events, we searched the TCGA database of lung cancer to identify genes correlated with *IGF1R* mRNA expression. Consistent with the PRKCSH results, GO functional enrichment analysis and GSEA of genes negatively correlated with *IGF1R* mRNA expression were significantly enriched in the adaptive immune system and natural killer cell-mediated cytotoxicity (Fig. 8g and Supplementary Fig. 9c). These data suggest that PRKCSH may regulate the expression of genes related to TNFSF resistance via IGF1R-dependent transcriptional activation, although the role of PRKCSH in transcriptional regulation is quite unknown.

To determine the in vivo relationship between the PRKCSH-IGF1R axis and NK cell-mediated antitumor effects, we used a xenograft tumor model of IL2Rg gene-deleted NOD/SCID mice (NIG mice), which was optimized to determine the antitumor effects induced by NK cells^38^. Three weeks after inoculation with A549-GFP cells expressing shCon or shPRK, the mice were treated with hIL2-NK-92 cells (Fig. 8h). Administration of hIL2-NK-92 cells using intravenous (I.V.) injection for 3 weeks in mice bearing A549-shCon xenografts resulted in marginal changes in tumor weight and volume, whereas administration of hIL2-NK-92 cells significantly reduced tumor weight and volume in mice bearing A549-shPRK xenografts without any effect on body weight (Fig. 8i, j, Supplementary Fig. 10, and Supplementary Table 2). To investigate NK cell-mediated tumor killing and NK cell infiltration in a xenograft mouse model, we performed immunohistochemical analyses using antibodies against cleaved PARP1 and NKG2D in tumor tissues. Histological data revealed that PRKCSH depletion enhanced the sensitivity of NK cell-mediated tumor cell death and NK cell infiltration into the tumor tissues (Fig. 8k). Considering the levels of NK cell infiltration, our findings revealed that the actual tumor size and weight were significantly different between the shPRK and shCon groups. These data demonstrate that aberrant enhancement of the PRKCSH-IGF1R axis in tumor cells is involved in the weakening of NK cell-mediated antitumor effects.

### Discussion

We previously reported that PRKCSH expression is upregulated in various cancer tissues, such as lung, liver, colon, gastric, and breast cancer tissues^26^. Our previous study demonstrated that PRKCSH promotes IRE1α activity in liver cancer cells. However, the potential role of PRKCSH in the development and progression of lung cancer has not yet been elucidated. Here, we report for the first time that increased expression of PRKCSH in lung cancers is associated with loss of NK cell-mediated antitumor effects via adaptation to the TNFSF response, as clearly evidenced by the results of various experiments on tumor sphere formation, cell death, and in vivo tumorigenesis. Mechanistically, PRKCSH interrupts TNFSF-mediated apoptotic signaling by promoting IGF1R activation in tumor cells without affecting the expression of TNFSF receptors, resulting in the inhibition of tumor cell death (Supplementary Fig. 11).

We showed that acquired TRAIL-resistant cells exhibited features of TNF-α resistance. Although the mechanism for TNFSF resistance is complex, our data indicate that it is likely to present common factors for determining cross-resistance to TNFSF in tumor cells. Indeed, common factors may be involved in the regulation of intracellular survival signaling rather than the expression of death receptors. Notably, PRKCSH expression is increased in acquired TRAIL-resistant cells, whereas PRKCSH depletion in these cells leads to both TRAIL- and TNF-α-mediated activation of extrinsic and intrinsic death pathways. The effect of PRKCSH on TNFSF resistance can be observed in various cancer cells, including lung, colon, and hepatoma cells. Therefore, PRKCSH might be an important factor in acquiring cross-resistance in tumor cells against TNFSF. TNFSF is a representative effector molecule in the antitumor immune response and is produced by various immune cells, such as NK cells^3,10–13^. However, TNFSF promotes tumor growth and progression by bypassing TNFSF receptor-mediated death signals^8,11,39–42^, and the survival pathways are also associated with tumor resistance against NK cell-mediated antitumor immunity^43,44^. In the present study, PRKCSH depletion increased TNFSF sensitivity and NK cell-mediated antitumor effects in in vitro and xenograft mouse models. Thus, PRKCSH may be a critical target for overcoming tumor resistance against NK cell-mediated antitumor immunity as well as for combating TNFSF resistance.

Caspase-8 is upregulated in many types of tumors and plays roles in two pathways: cell death and growth^45,46^. Caspase-8 activity is regulated by phosphorylation; its phosphorylation level is substantially increased in tumor cells to suppress caspase-8 activity, leading to increased resistance to extrinsic apoptosis^47,48^. Caspase-8 phosphorylation attenuates DISC activity by inhibiting the polyubiquitination and autoproteolytic activity of caspase-8, which enhances tumor growth and migration^47,48^. Therefore, caspase-8 is an important target for improving the tumor microenvironment and enhancing antitumor immunity^47,49^. PRKCSH depletion suppresses caspase-8 phosphorylation but promotes caspase-8 ubiquitination and activation. It is clear that PRKCSH is critical for regulating the balance between the two caspase-8 modes of action, which may be involved in the regulation of NK cell-mediated antitumor immunity as well as tumor cell growth.

Mcl-1 is well known as a critical survival factor for diverse cell death stimuli in many tumor cells, including non-small cell lung cancer and melanoma cells^50–52^. Mcl-1 has recently been identified as the link between the extrinsic and intrinsic apoptotic pathways mediated by TRAIL^53^. Recently, Mcl-1 has become a promising target in cancer therapy because Mcl-1 is considered a key factor in overcoming tumor resistance to various therapeutics^54,55^. In the present study, Mcl-1 expression was decreased in PRKCSH-deficient cells under both basal and TRAIL stimulation conditions. Indeed, Mcl-1 expression in PRKCSH-deficient cells reversed TRAIL resistance, whereas Mcl-1 knockdown increased TRAIL sensitivity in lung cancer cells. Thus, our data suggest that PRKCSH contributes to tumor resistance against various cancer therapeutics as well as the TNFSF response via *Mcl-1* mRNA transcription regulation.

Generally, proteasomes and lysosomes represent the most important proteolytic machinery responsible for protein degradation and contribute to protein quality and quantity control. Endocytic receptor tyrosine kinases, such as IGF1R, are trafficked and degraded into lysosomes. A previous report demonstrated that PRKCSH depletion induces autophagy activation^37^. Therefore, we hypothesized that PRKCSH contributes to IGF1R degradation by regulating lysosomal activity. Furthermore, the receptors are primarily expressed in the ER and are processed by the folding machinery or degraded in the proteasome through the ERAD pathway. In particular, PRKCSH has an ER chaperone-like function and contributes to ER protein quality control, which is involved in protecting receptor proteins, such as the TRP family channel PKD2, against major ERAD component HERPUD1-mediated degradation^23^. In the present study, PRKCSH protected IGF1R from proteasome-mediated degradation. However, PRKCSH depletion-induced lysosomal activation was not associated with the regulation of IGF1R stability. Furthermore, PRKCSH competes with HERPUD1 to regulate IGF1R stability. The Coil-E/P domain of PRKCSH is essential for its interaction with IGF1R, whereas its MRH domain is critical for inhibiting the interaction between HERPUD1 and IGF1R. Indeed, we demonstrated that PRKCSH prolonged the half-life of the IGF1R protein, but not the EGFR protein, leading to increased IGF1R expression on the plasma membrane, promoting IGF1R activation, and inhibiting TNFSF sensitivity. Our findings suggest that PRKCSH selectively regulates the stability of specific proteins in the ER, such as IGF1R and PKD2.

The gain of IGF1R in PRKCSH-deficient cells increased caspase-8 phosphorylation and Mcl-1 expression levels, whereas IGF1R knockdown reduced caspase-8 phosphorylation and Mcl-1 expression levels in lung cancer cells. IGF1R downstream kinases, such as AKT and ERK, regulate caspase-8 activity and *Mcl-1* mRNA expression^53,56,57^. Thus, PRKCSH in the ER could indirectly regulate *Mcl-1* mRNA expression and caspase-8 phosphorylation by increasing IGF1R activity. IGF1R downstream transcription factors upregulated by PRKCSH may also be involved in the expression of various genes related to TNFSF resistance. In tumor pathology, IGF1R plays an important role in tumor resistance to antitumor immunity^58–60^. In particular, IGF1R inhibition restores the dendritic cell-mediated antitumor response in ovarian cancer^59^. A previous report demonstrated that TRAIL induces IGF1R activation in gastric cancer models, leading to resistance to TRAIL-induced cell death^35^. Therefore, the potential use of IGF1R-targeting strategies in lung cancer has been considered in several preclinical studies and clinical trials^61,62^. In the present study, IGF1R expression was found to be upregulated in lung cancer patient tissues, and its protein levels were correlated with PRKCSH protein levels. Upregulation of these two proteins was closely associated with an unfavorable prognosis in lung cancer patients, suggesting that the PRKCSH-IGF1R axis is important not only as a reference for prognostic markers in lung cancer but also as a critical target for overcoming tumor resistance against NK cell-mediated antitumor immunity.

Taken together, these results show that PRKCSH contributes to the adaptation of tumor cells against TNFSF cytotoxicity by increasing the half-life of IGF1R, which is involved in tumor resistance to antitumor immunity and various cancer therapeutics. Therefore, targeting PRKCSH may be a promising therapeutic strategy for various tumors including IGF1R-related lung cancer.

### Supplementary information

Supplemental information

Dataset 1

Dataset 2

## Data Availability

All data supporting the findings of this study are available within the article and its Supplementary Information files and from the corresponding author upon reasonable request.
