# Supplementary material for: PRKCSH contributes to TNFSF resistance by extending IGF1R half-life and activation in lung cancer
Source: Exp Mol Med. 2024 Jan 10;56(1):192–209. doi: 10.1038/s12276-023-01147-1 (PMC10834952; doi:10.1038/s12276-023-01147-1)
Supplement: Supplementary file 1 — Supplemental information [file 12276_2023_1147_MOESM1_ESM.pdf]

# Supplementary Materials for

## PRKCSH contributes to TNFSF resistance by extending IGF1R half-life and activation in lung cancer

Gu-Choul Shin *et al*.

Corresponding author: Gu-Choul Shin, rnelr@naver.com; Kyun-Hwan Kim, khkim10@skku.edu

**The file includes**

Supplementary Table 1 and 2

Supplementary Fig. 1 to 11

**Supplementary Table 1.** List of primers used in this study

| Name | Sequences (5′ → 3′) | Used |
|---|---|---|
| PRKCSH-F | GGCGTCTCCCTCACCAATCATC | qPCR |
| PRKCSH-R | TCTCCTCCCGTGCCTTCTTCCAGT | qPCR |
| GAPDH-F | ATCATCCCTGCCTCTACTGG | qPCR |
| GAPDH-R | TGGGTGTCGCTGTTGAAGTC | qPCR |
| Mcl1-F | CTCGGTACCTTCGGGAGCAGGC | qPCR |
| Mcl1-R | CCAGCAGCACATTCCTGATGCC | qPCR |
| Mcl1L-F | CCAAGAAAGCTGCATCGAACCAT | Semi-qPCR |
| Mcl1L-R | CAGCACATTCCTGATGCCACCT | Semi-qPCR |
| IGF1R-F | GAAGTCTGGCTCCGGAGGAGGGTC | qPCR |
| IGF1R-R | ATGTGGAGGTAGCCCTCGATCAC | qPCR |

**Supplementary Table 2.** The antitumor effect of hIL2-NK-92 cells in A549-shCon and A549-shPRK groups

| Group | Treatment | T/C (%) | TGI (%) | No. of survival mice |
|---|---|---|---|---|
| A549-shCon | PBS | 99.9 | 0.0 | 10/10 |
| A549-shCon + hIL2-NK-92 | hIL2-NK-92 | 71.3 | 28.6 | 10/10 |
| A549-shPRK | PBS | 100.3 | 0.0 | 10/10 |
| A549-shPRK + hIL2-NK-92 | hIL2-NK-92 | 51.5 | 48.8 | 10/10 |

T/C: Percentage of tumor weight for hIL2-NK-92 *vs.* PBS-treated group

TGI: Percentage of tumor growth inhibition

## Supplementary Figures

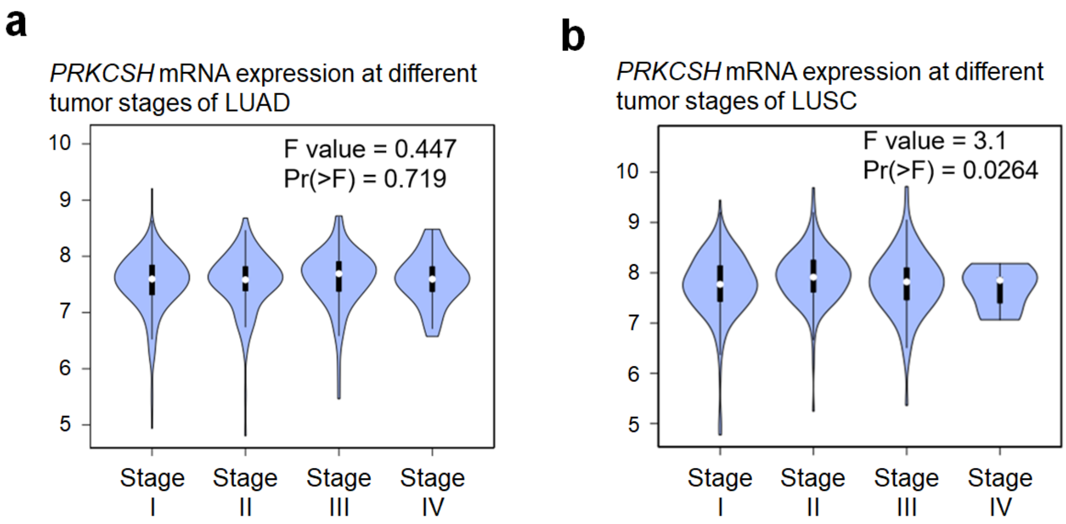


**Supplementary Fig. 1: Expression of *PRKCSH* mRNA at different tumor stages of lung cancer tissues including lung adenocarcinoma (LUAD) (a) and lung squamous cell carcinoma (LUSC) (b).**

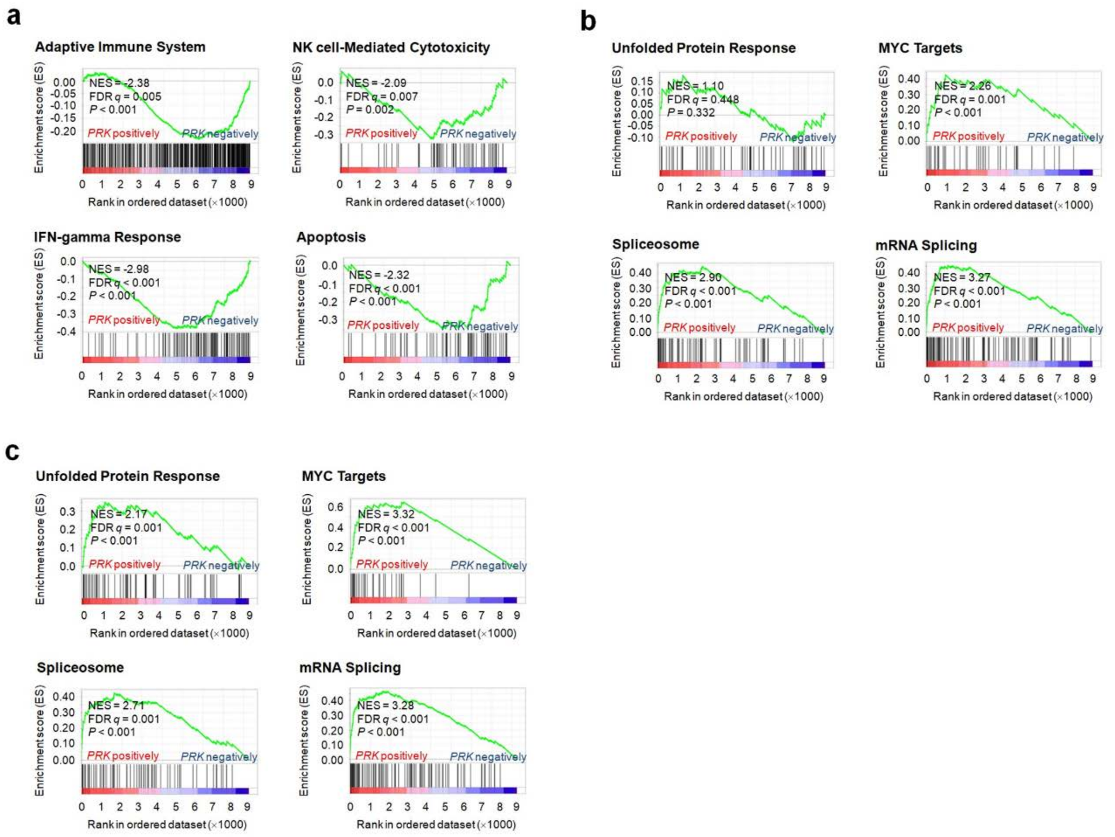


**Supplementary Fig. 2: Gene-set enrichment analysis (GSEA) of genes co-expressed with PRKCSH mRNA in lung cancer tissues. a** Functional pathway analysis using GSEA of genes negatively co-expressed with *PRKCSH* mRNA in lung adenocarcinoma tissues. **b** Functional pathway analysis using GSEA of positively co-expressed genes with *PRKCSH* mRNA in lung squamous cell carcinoma tissues. **c** Functional pathway analysis using GSEA of genes positively co-expressed with *PRKCSH* mRNA in lung adenocarcinoma tissues. NES, normalized enrichment score; FDR *q,* false discovery rate *q* value.

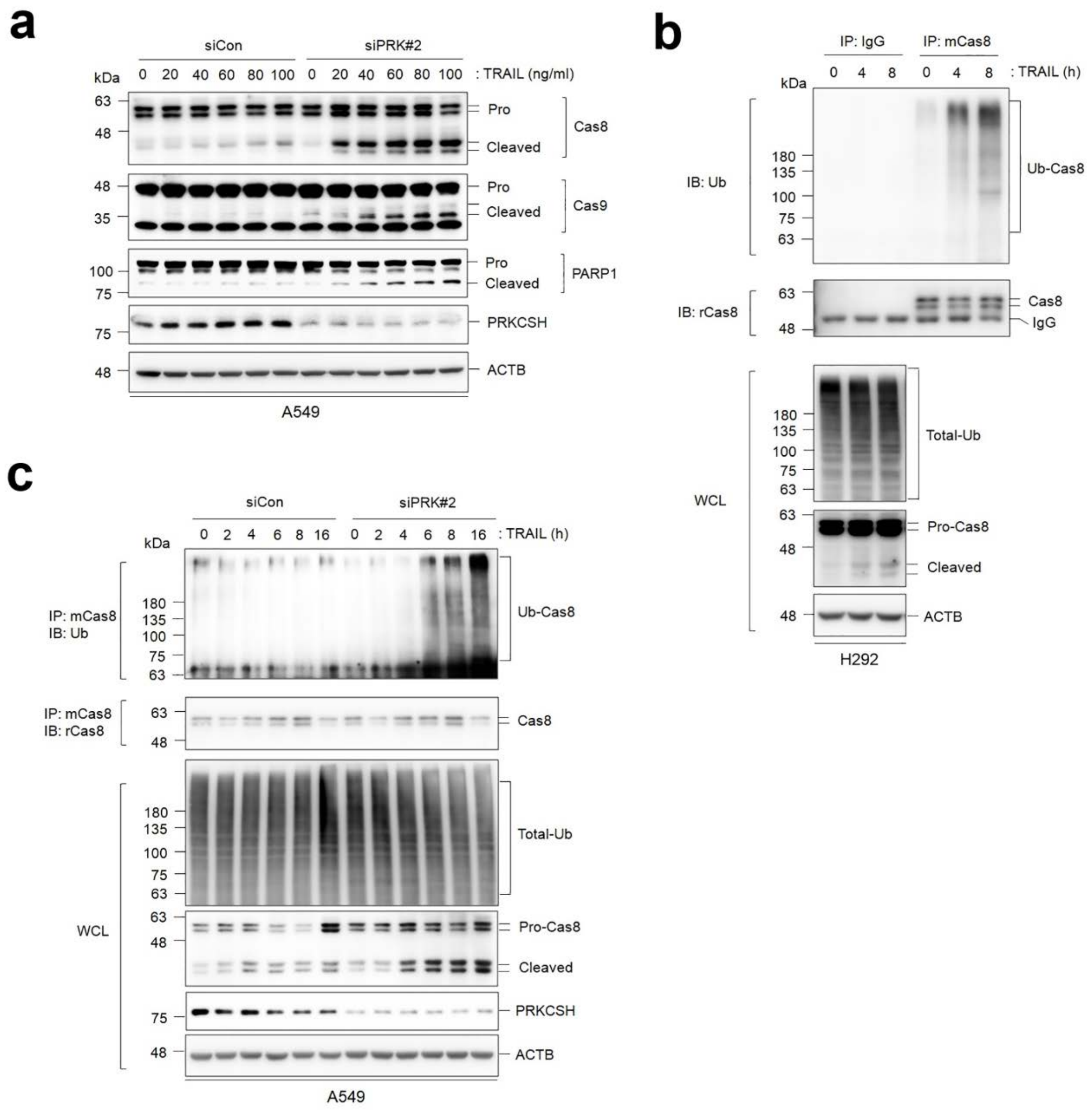


**Supplementary Fig. 3: Analysis of capase-8 and-9 activation, and capaspse-8 polyubiquitination in transiently PRKCSH knockdown cells. a** Activation of caspases in A549 cells transiently transfected with siRNA to *PRKCSH* gene, as determined by immunoblotting. Cells were transfected with siCon or siPRK#2 followed by treatment with TRAIL at the indicated concentrations for 24 h. Activation of caspases in cell lysates was analyzed by immunoblotting with anti-caspase-8, anti-caspase-9, and PARP1 antibodies.

ACTB was used as a loading control. **b** Detection of TRAIL-mediated ubiquitination of endogenous caspase-8 in lung cancer. Cells were treated with 50 ng/mL TRAIL for the indicated times, and ubiquitinated caspase-8 in cell lysates was pulled down with an anti-caspase-8 antibody, followed by immunoblot analysis with anti-ubiquitin and anti-caspase-8 antibodies. **c** Analysis of TRAIL-mediated ubiquitination of caspase-8 in A549 cells transiently transfected with siPRK#2 or siCon. Cells were treated with 50 ng/ml TRAIL for the indicated times, and ubiquitinated caspase-8 in cell lysates was pulled down with anti-caspase-8 antibody, followed by immunoblot analysis with anti-ubiquitin antibody.

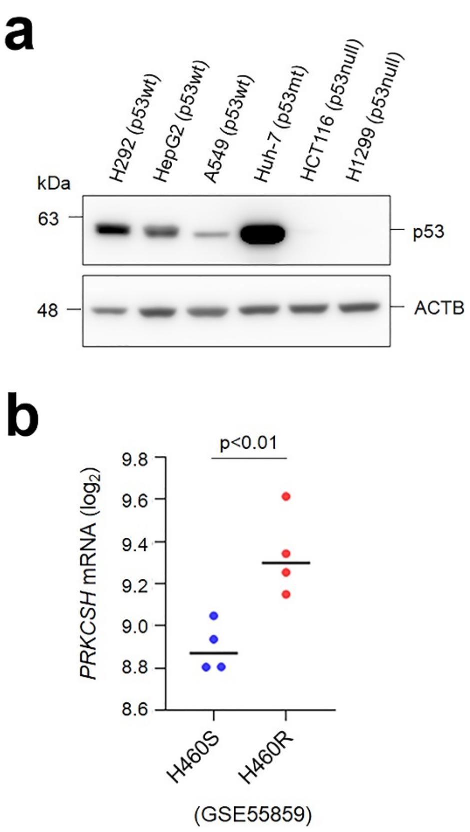


**Supplementary Fig. 4: Analysis of p53 expression and PRKCSH expression in lung cancer cells. a** Immunoblot analysis of p53 expression in the cancer cells used in this study. p53 was detectable in H292 (p53wt), HepG2 (p53wt), A549 (p53wt), and Huh-7 (p53mt) cells, whereas p53 was not expressed in HCT116 (p53null) and H1299 (p53null) cells. **b** Analysis of *PRKCSH* mRNA levels in TRAIL-resistant H460 (H460R) and parent H460 (H460S) cells using a public data set (GSE55859).

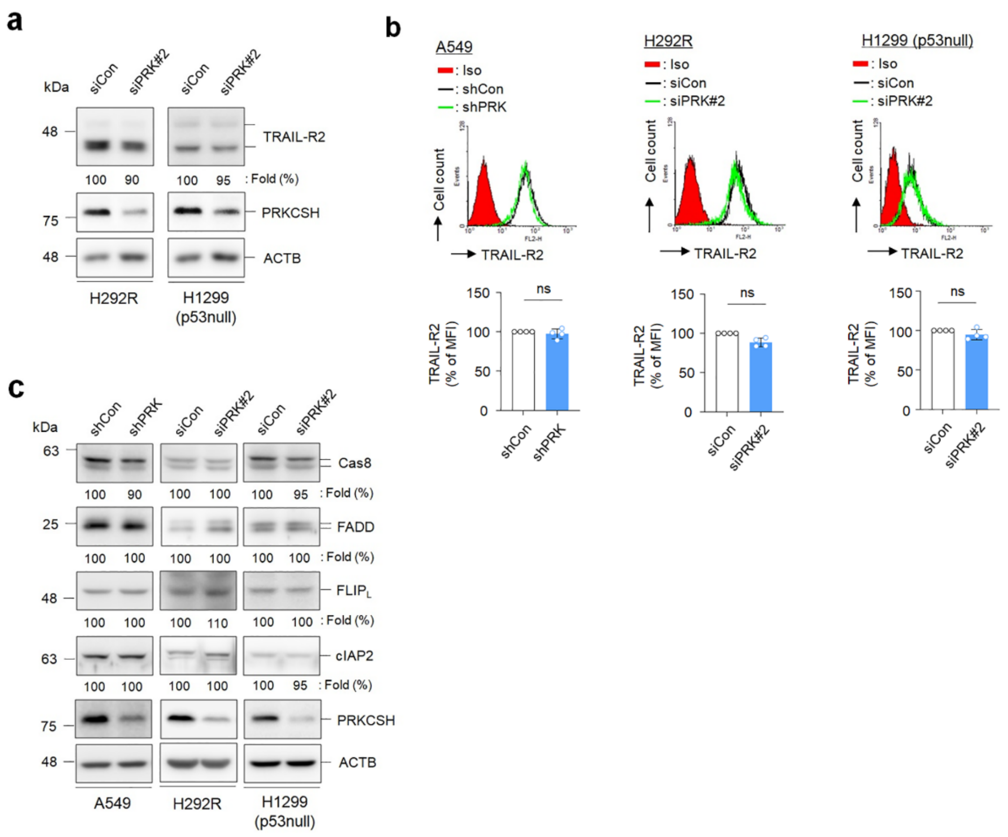


**Supplementary Fig. 5: Effect of PRKCSH depletion on the expression of genes related to TRAIL-mediated apoptosis. a** Immunoblot analysis of TRAIL-R2 expression in other lung cancer cell lines, including H292R and H1299 (p53null). Quantitative analysis of TRAIL-R2 expression levels were calculated by densitometry measurement of band intensity and shown to each band below. **b** Analysis of TRAIL-R2 expression levels on the surface of PRKCSH-deficient cells, including A549-shPRK, H292R transfected with siPRK#2, and H1299 (p53null) transfected with siPRK#2. Representative flow cytometry data and quantitative analysis of TRAIL-R2 expression levels on the surface of each cell line are shown. **c** Immunoblot analysis of other apoptosis-related genes, including caspase-8, FADD, FLIP, and cIAP2 in PRKCSH-deficient A549, H292R, and H1299 (p53null) cells. NS, non-significant.

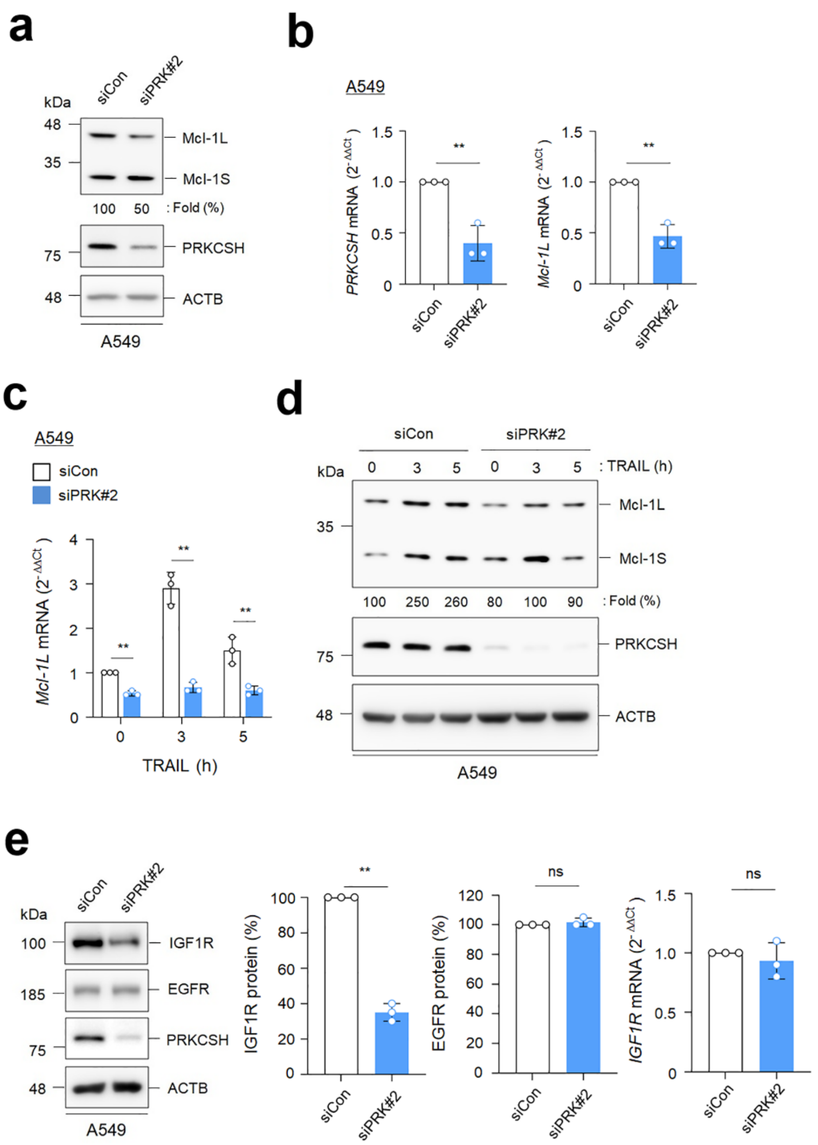


**Supplementary Fig. 6: Analysis of Mcl-1 and IGF1R expression in A549 cells transiently transfected with siPRK#2 or siCon. a-b** The expression of Mcl-1 protein (a) and mRNA (b) levels were analyzed by immunoblotting and qPCR, respectively. **c-d** TRAIL-induced expression of Mcl-1 mRNA (c) and protein (d) were analyzed by qPCR and immunoblotting, respectively. **p < 0.01. **e** Expression of IGF1R and EGFR protein levels was analyzed by immunoblotting. Data are representative immunoblots and quantitative analyses

of IGF1R and EGFR expression levels. *IGF1R* mRNA levels were analyzed using qPCR. **p < 0.01. NS, non-significant.

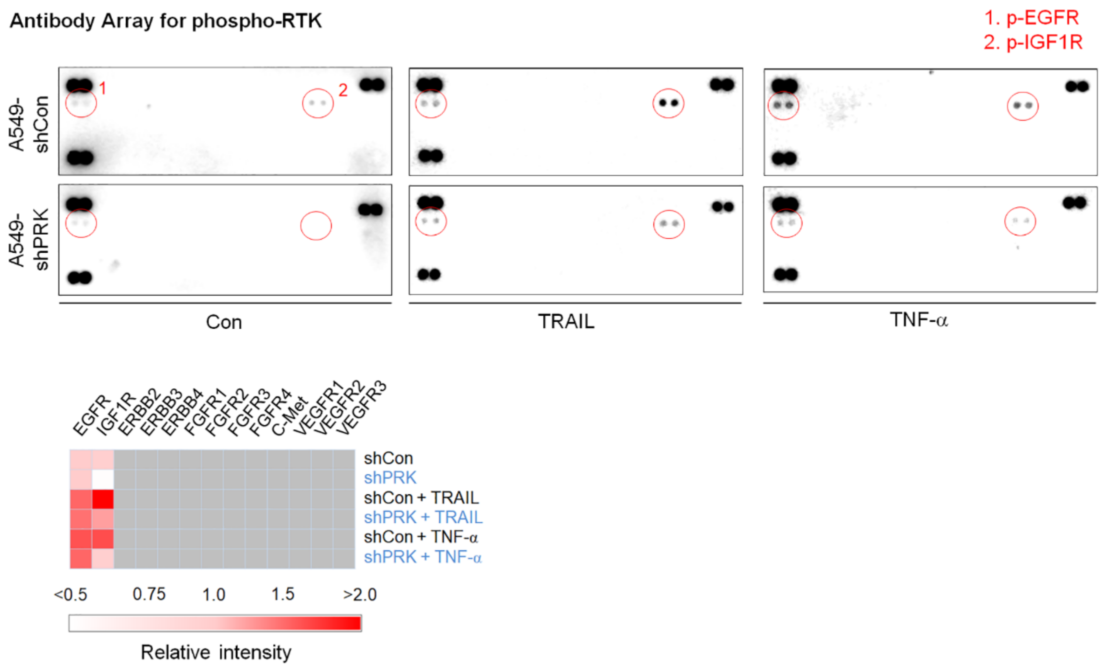


**Supplementary Fig. 7:** Antibody array analysis of TNFSF-mediated receptor tyrosine kinases (RTKs) activation in PRKCSH-deficient cells. The protein extracts from A549-shCon and -shPRK cells treated with or without 50 ng/ml TRAIL or 5 ng/ml TNF-α were incubated onto antibody array for phospho-RTKs, followed by determination of the signal intensity of each spot. Representative antibody array and quantitative analysis of RTKs phosphorylation are shown. Signal intensity of each RTK phosphorylation is indicated by the heatmap. Each cell line is color-coded based on the relative abundance of each RTK phosphorylation in the shPRK cells or TNFSF-treated cells relative to the shCon cells.

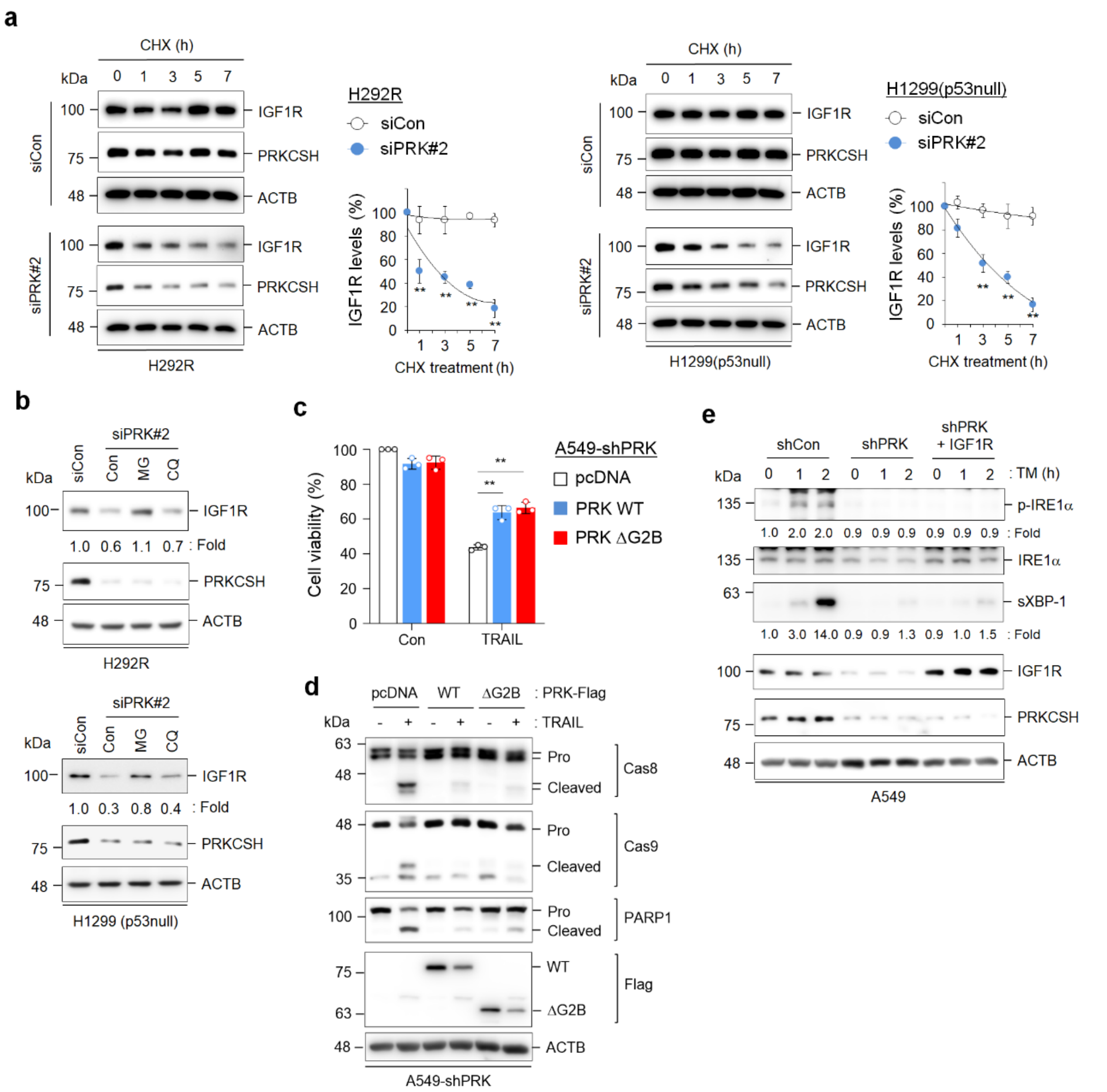


**Supplementary Fig. 8: Mechanism of PRKCSH-mediated extension of IGF1R protein lifespan, the impact of its overexpression on IGF1R lifespan and TNFSF-mediated tumor cell killing, and the role of IGF1R on PRKCSH-mediated promotion of IRE1α activation. a** The effect of PRKCSH depletion on the IGF1R protein half-life in lung cancer cells. H292R and H1299 (p53null) cells transfected with siPRK#2 or siCon were treated with cycloheximide for indicated times. The lifespan of IGF1R protein in cell lysates was analyzed using immunoblotting. Representative immunoblots and quantitative analysis of

IGF1R protein levels in each cell line are shown. **b** The effect of proteasome or lysosome inhibitors on the regulation of IGF1R protein lifespan in lung cancer cells. H292R and H1299 (p53null) cells transfected with siPRK#2 or siCon were treated with MG132 (proteasome inhibitor) or chloroquine (lysosome inhibitor) for 24 h. IGF1R protein levels were determined using immunoblotting. **c-d** The effect of PRKCSH WT or ΔG2B mutant overexpression on TNFSF-mediated tumor cell killing in PRKCSH-deficient cells. A549-shPRK cells were transfected with PRKCSH WT or ΔG2B mutant plasmid, followed by treatment with TRAIL for 24 h. TNFSF-mediated cytotoxicity was analyzed by MTT assay (**c**) and activation of caspases was analyzed by immunoblotting (**d**). **e** The role of IGF1R on PRKCSH-mediated promoting IRE1α activation. A549-shPRK cells were infected with lentivirus containing IGF1R cDNA plasmid, followed by treatment with tunicamycin for indicated times. The levels of IRE1α activation and its downstream XBP-1 splicing in A549-shCon, -shPRK, and -shPRK overexpressed with IGF1R cells were analyzed using immunoblotting. $^{**}p < 0.01$.

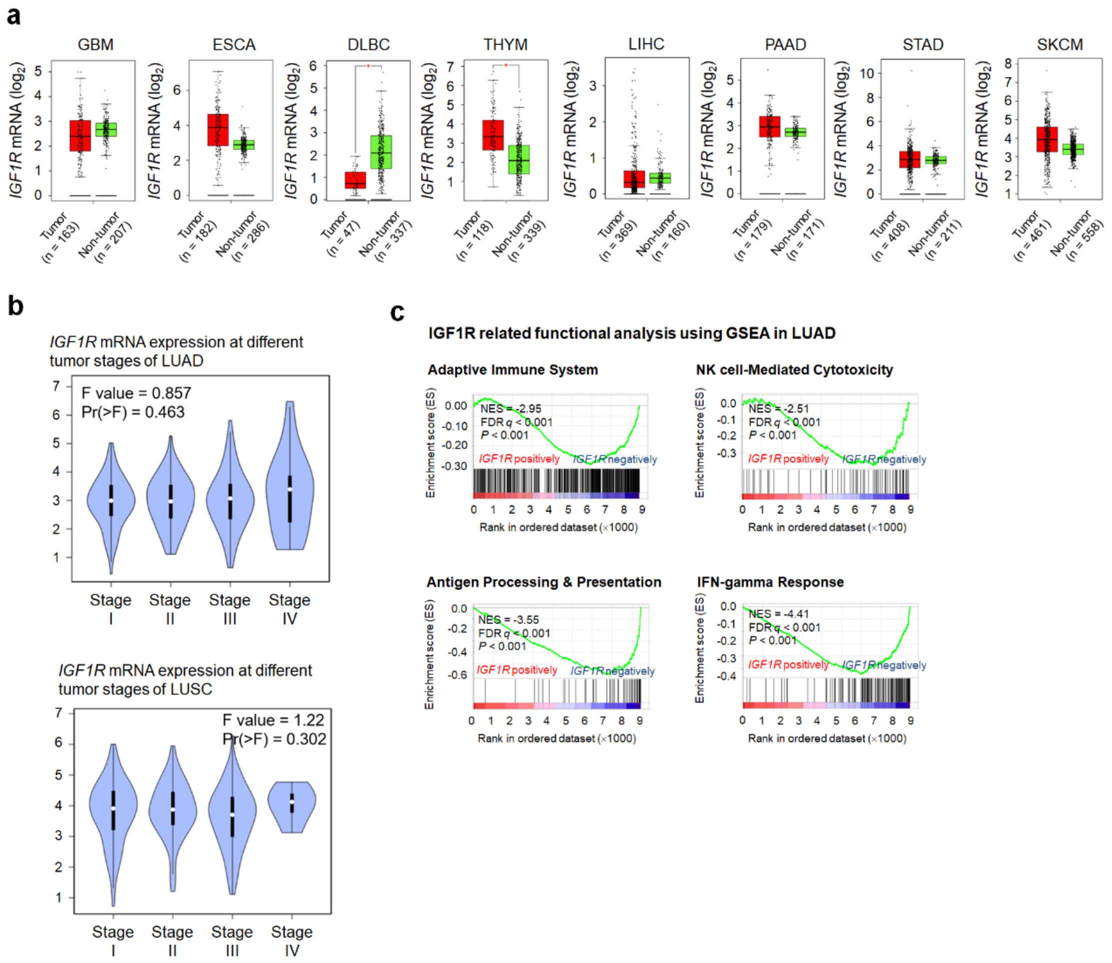


**Supplementary Fig. 9: Analysis of *IGF1R* mRNA expression in various cancer tissues, its expression at different tumor stages of lung cancer tissues, and GSEA of genes related to *IGF1R* mRNA expression. a** Quantitative analysis of *IGF1R* mRNA expression levels between normal and cancerous tissues including glioblastoma multiforme (GBM), esophageal carcinoma (ESCA), lymphoid neoplasm- diffuse large B-cell lymphoma (DLBC), thymoma (THYM), liver hepatocellular carcinoma (LIHC), pancreatic adenocarcinoma (PAAD), stomach adenocarcinoma (STAD), and skin cutaneous melanoma (SKCM). **b** Analysis of *IGF1R* mRNA expression at

different tumor stages of lung adenocarcinoma (LUAD) and lung squamous cell carcinoma (LUSC). **c** GSEA of genes co-expressed with *IGF1R* mRNA in LUAD tissues. Functional pathway analysis of genes negatively co-expressed with *IGF1R* mRNA. NES, normalized enrichment score; FDR *q,* false discovery rate, *q* value.

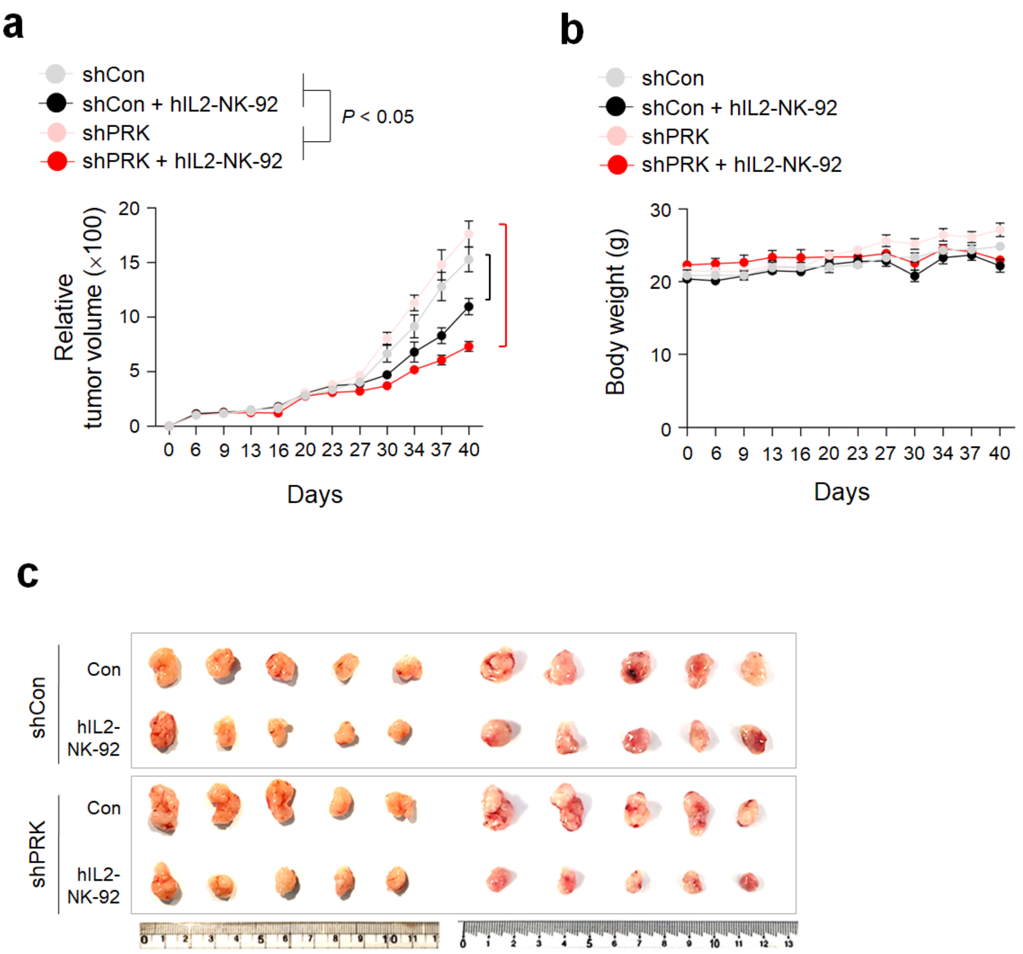


**Supplementary Fig. 10: *In vivo* relationship between PRKCSH and natural killer cell-mediated antitumor effect in the xenograft tumor model of IL-2Rg deficient NOD/SCID (NIG) mice. a-b** Relative tumor volume (a) and body weight (b) were measured for 40 days after inoculation of tumor cells. A549-shCon and -shPRK cells were inoculated subcutaneously into the mice, and hIL2-NK-92 cells were injected intravenously into the mice at 3 weeks after inoculation. Tumor volume and body weight were measured. **c** Mice were sacrificed on day 40 after the inoculation of tumor cells, and tumor sizes were measured. These data come from repeating the whole experiment independently twice (each group, n = 10).

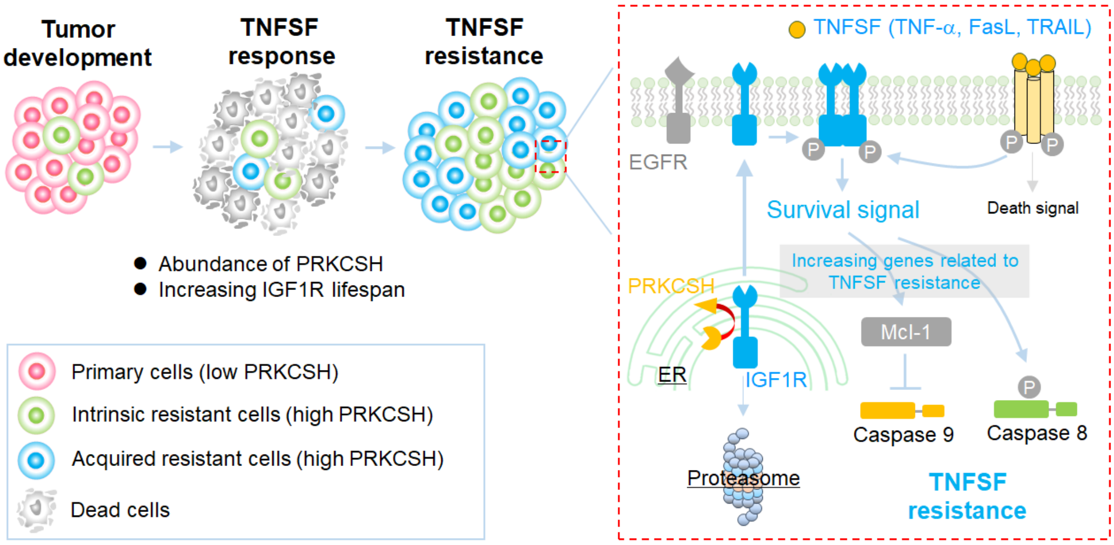


**Supplementary Fig. 11: Schematic summary for the role of PRKCSH in TNFSF resistance in lung cancer.** TNFSF is important for the T and NK cell-based antitumor activity. TNFSF resistance has attracted attention in the field of cancer treatment, because TNFSF resistance is involved in tumor development and prognosis, incapacitation of antitumor immunity, and chemotherapeutics resistance. PRKCSH abundance in cancer cells contributes to extending the lifespan of IGF1R protein and promoting its activation, but not EGFR protein, suggesting that PRKCSH can selectively regulate the specific protein lifespan through physiological interaction in the endoplasmic reticulum. Extending IGF1R activation leads to increasing caspase-8 phosphorylation and Mcl-1 mRNA expression under both basal and TNFSF treatment. PRKCSH abundance turns TNFSF death signal into survival signal, leading to TNFSF resistance of lung cancers.
