# Supplementary figures and images for: PRKCSH contributes to TNFSF resistance by extending IGF1R half-life and activation in lung cancer
Source: Exp Mol Med. 2024 Jan 10;56(1):192–209. doi: 10.1038/s12276-023-01147-1 (PMC10834952; doi:10.1038/s12276-023-01147-1)

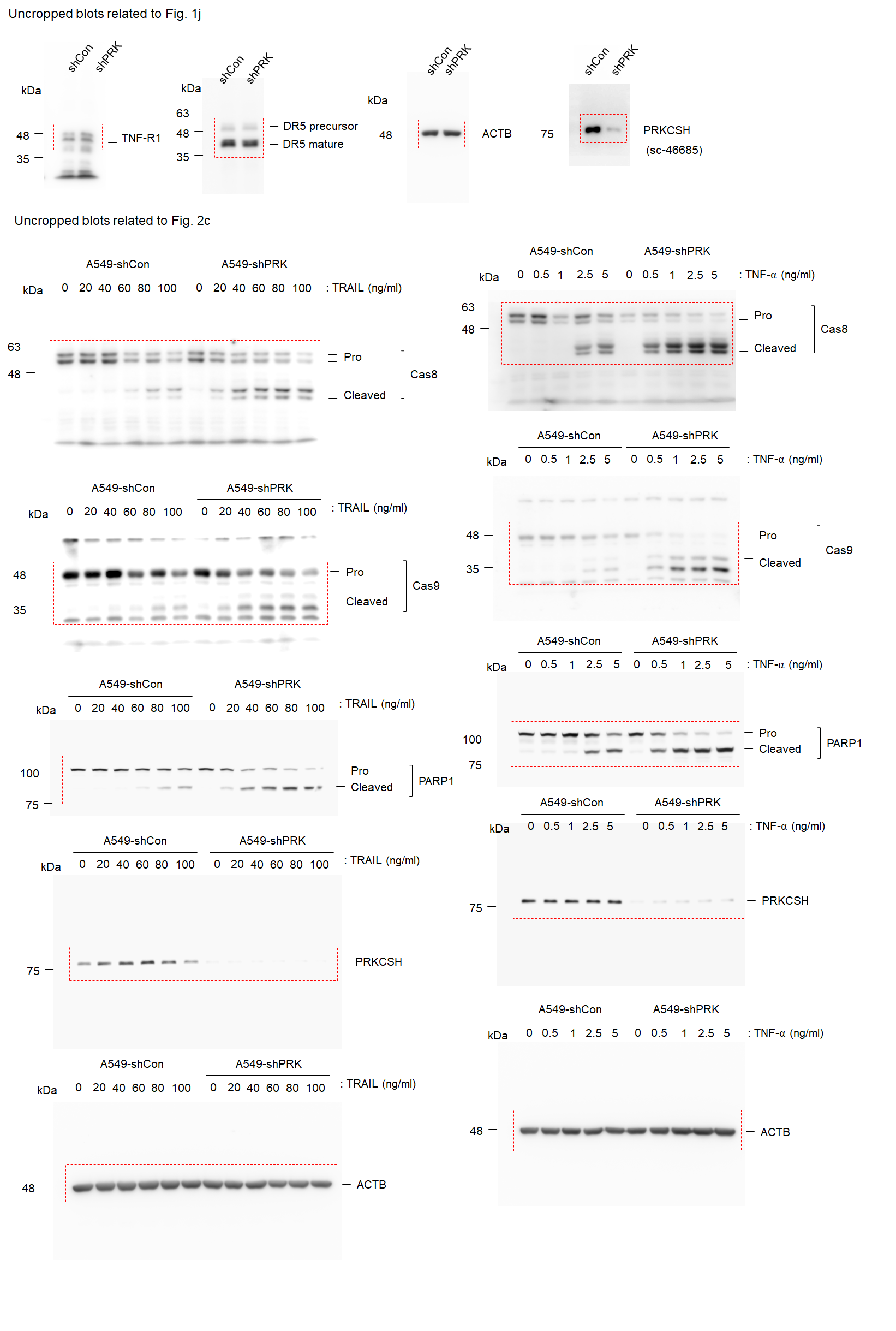

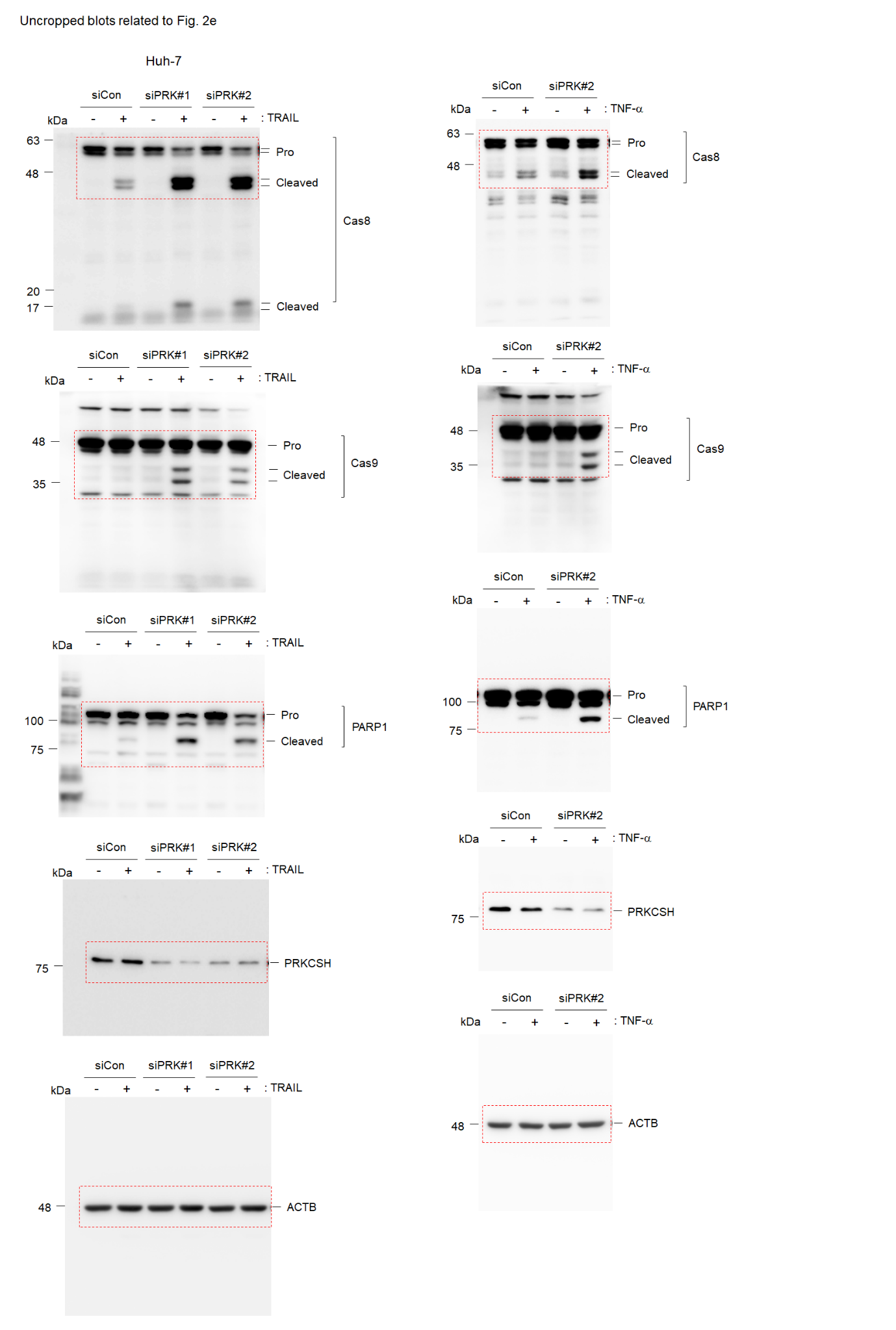

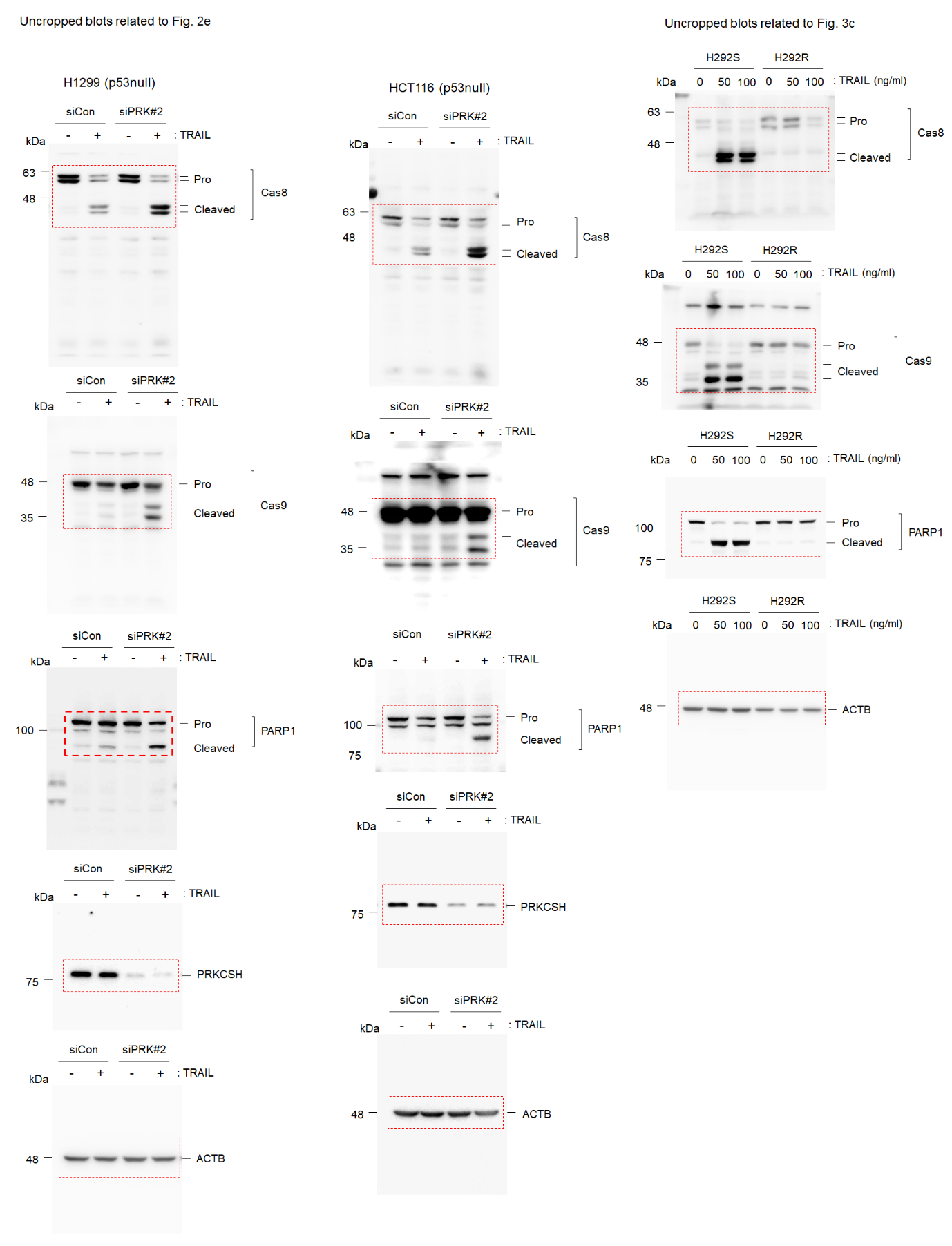

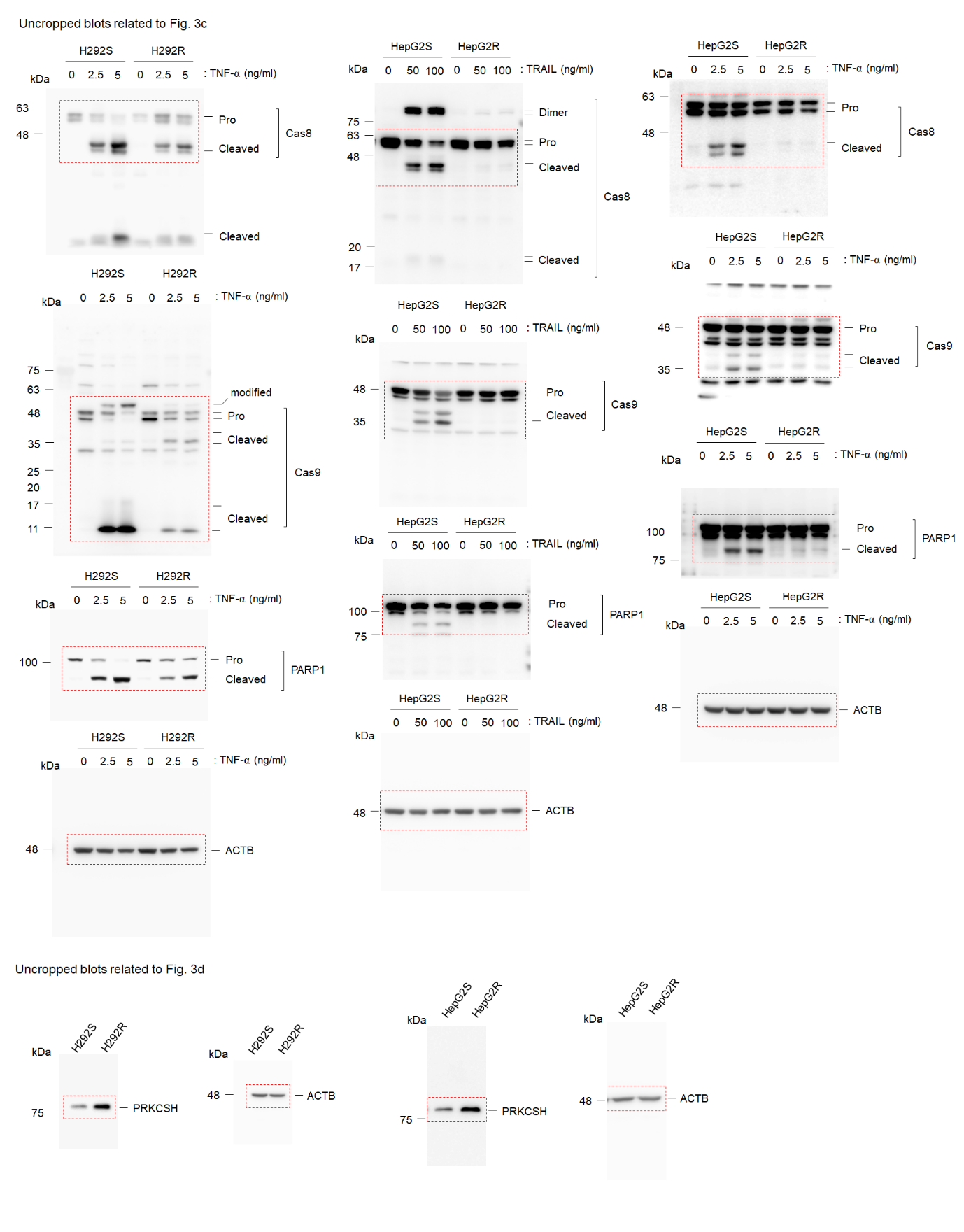

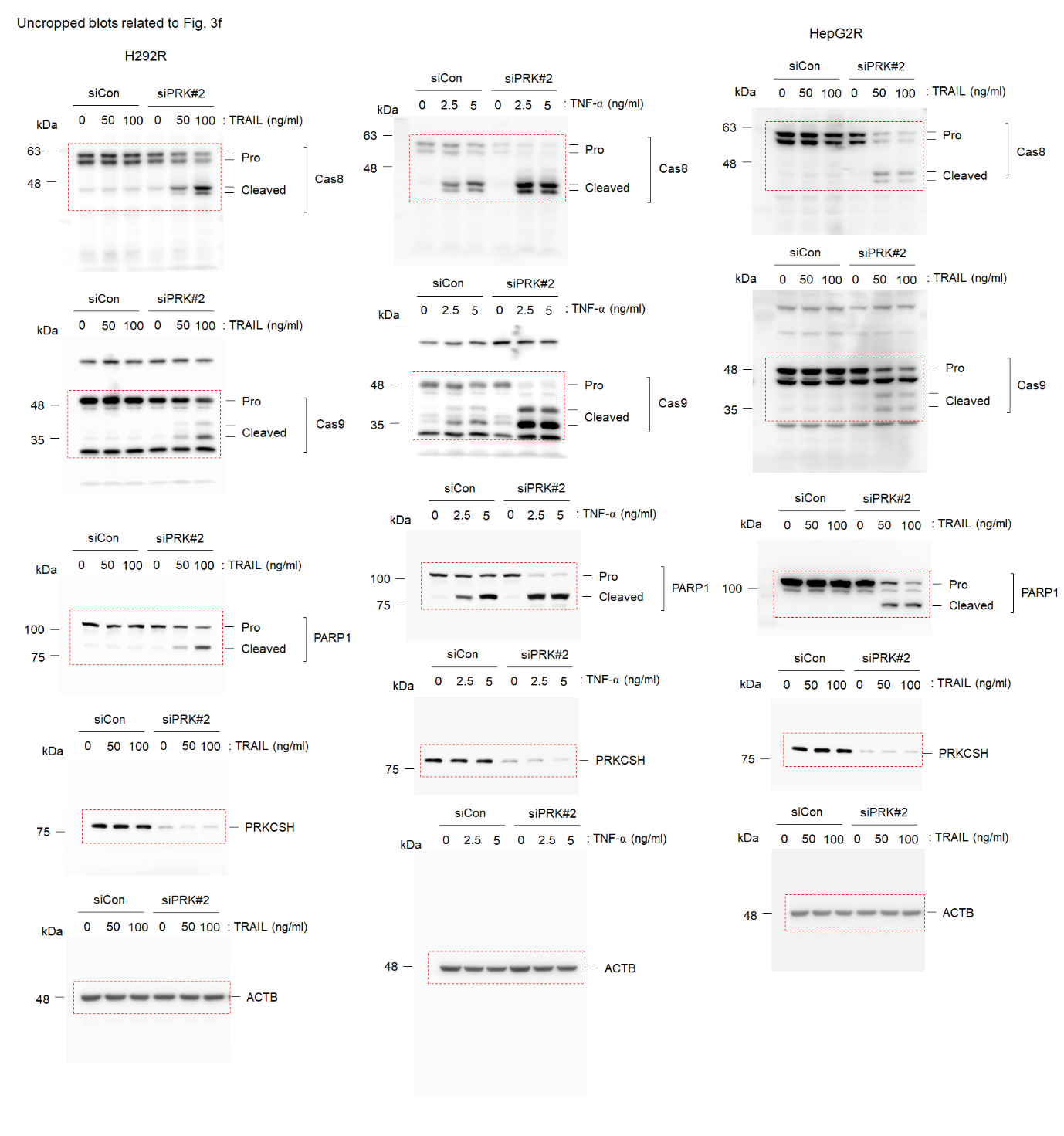

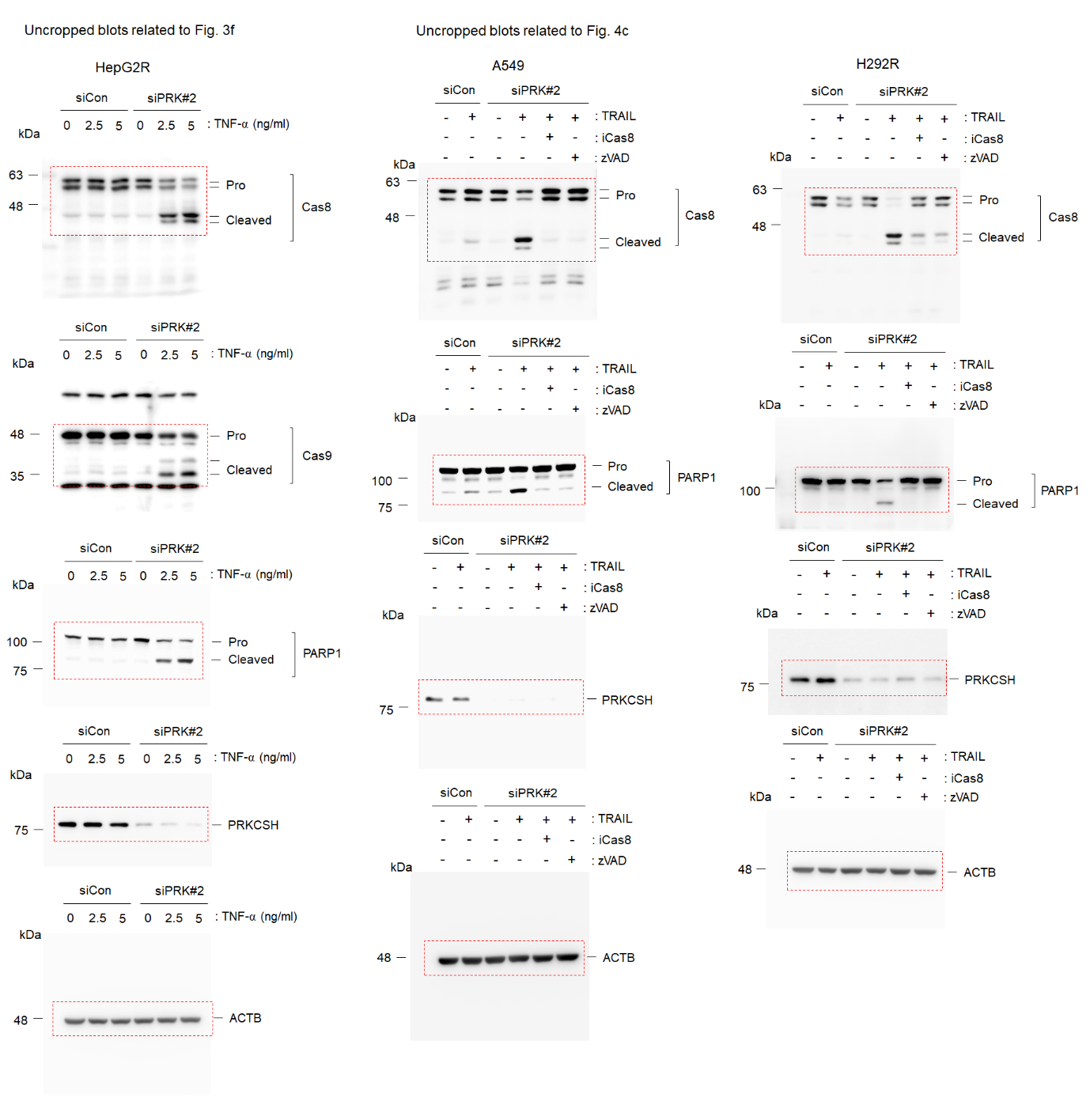

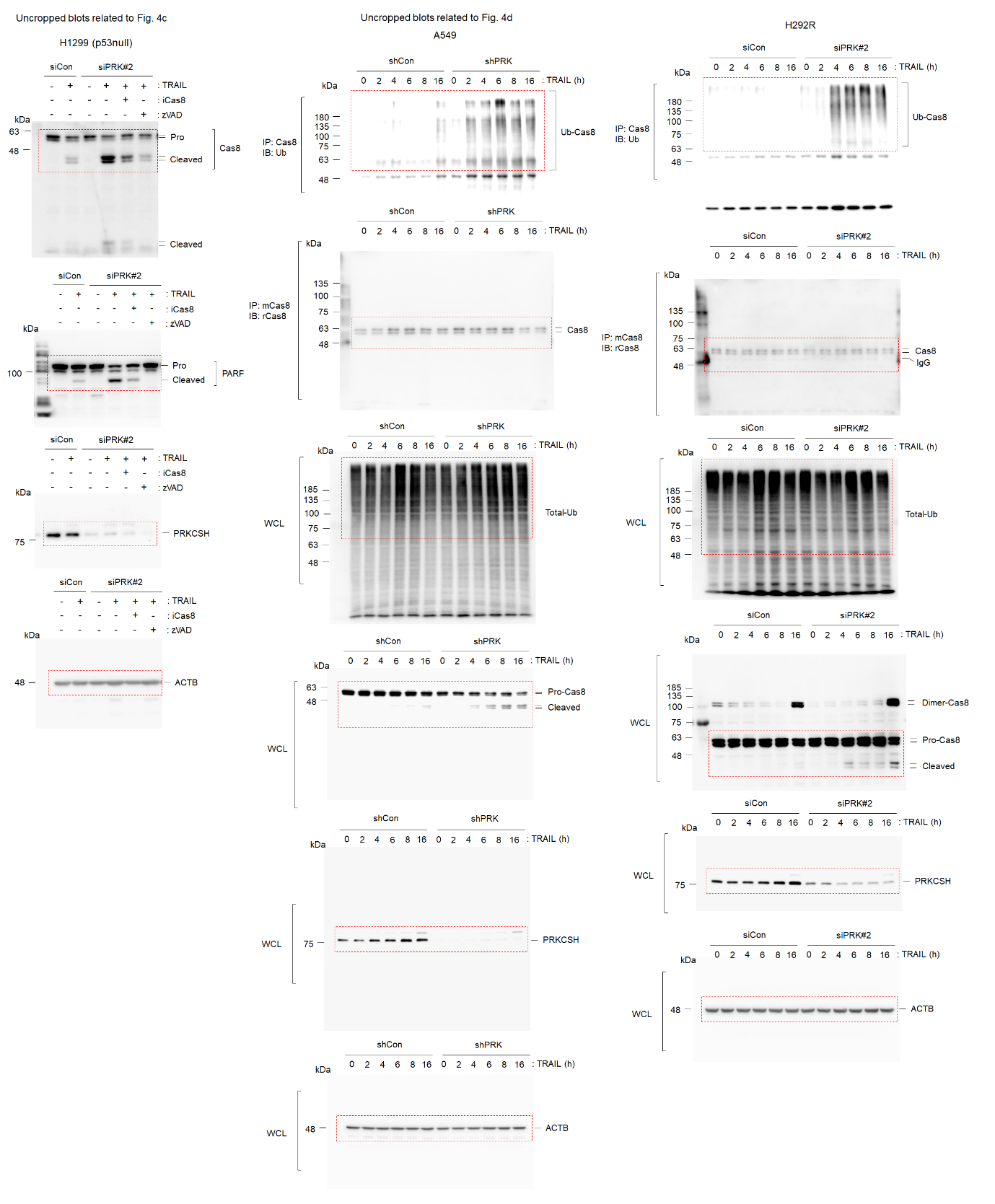

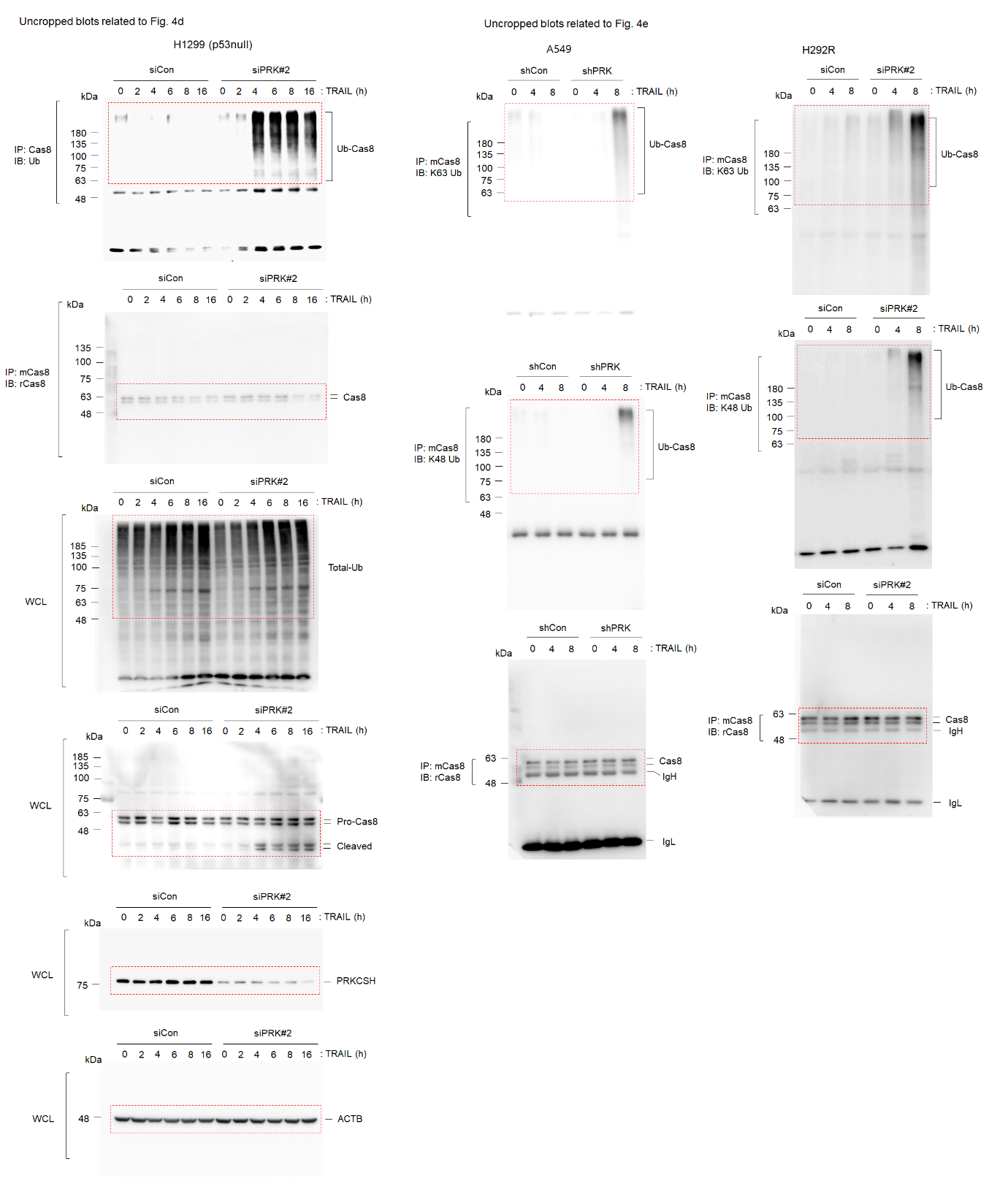

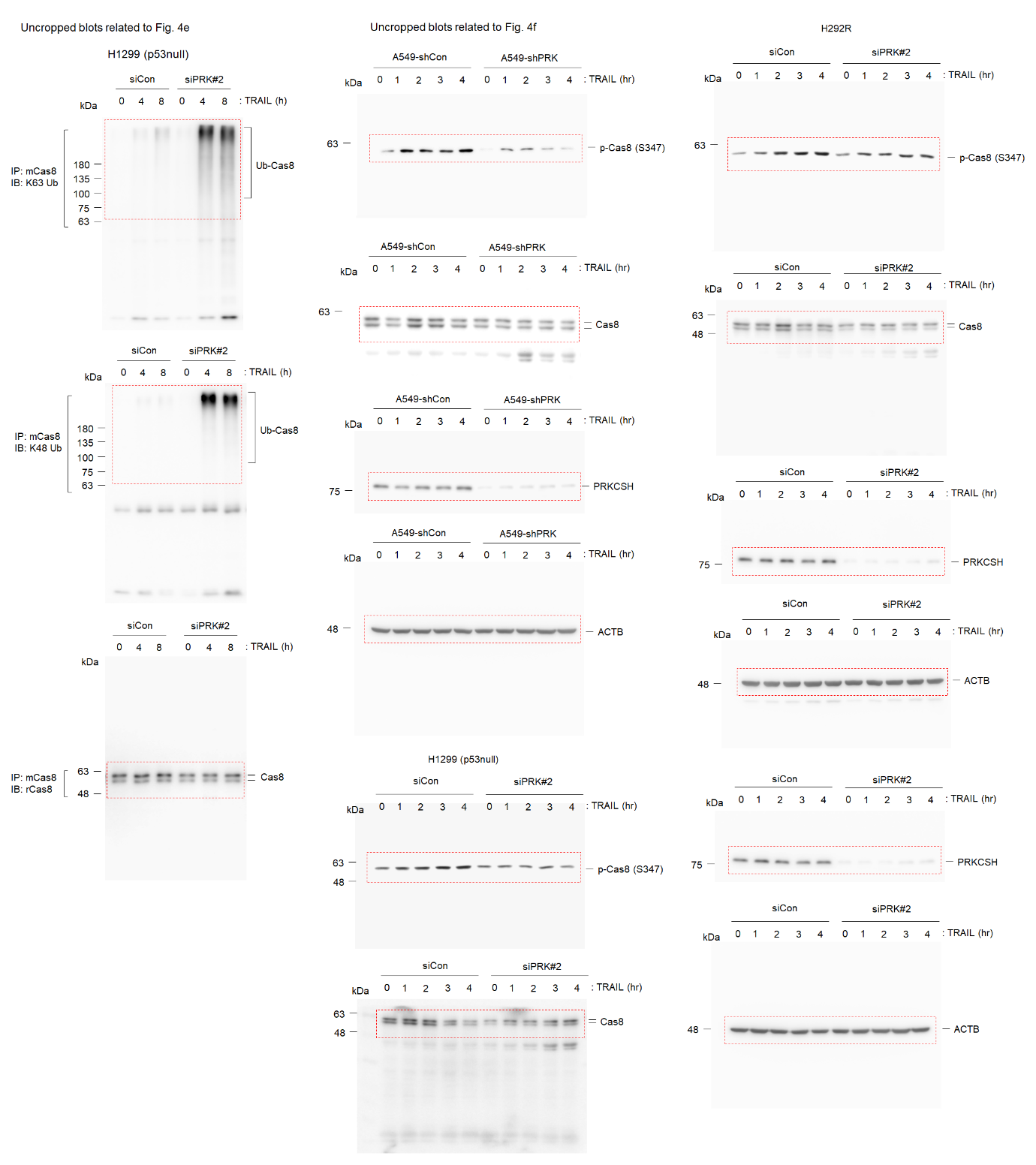


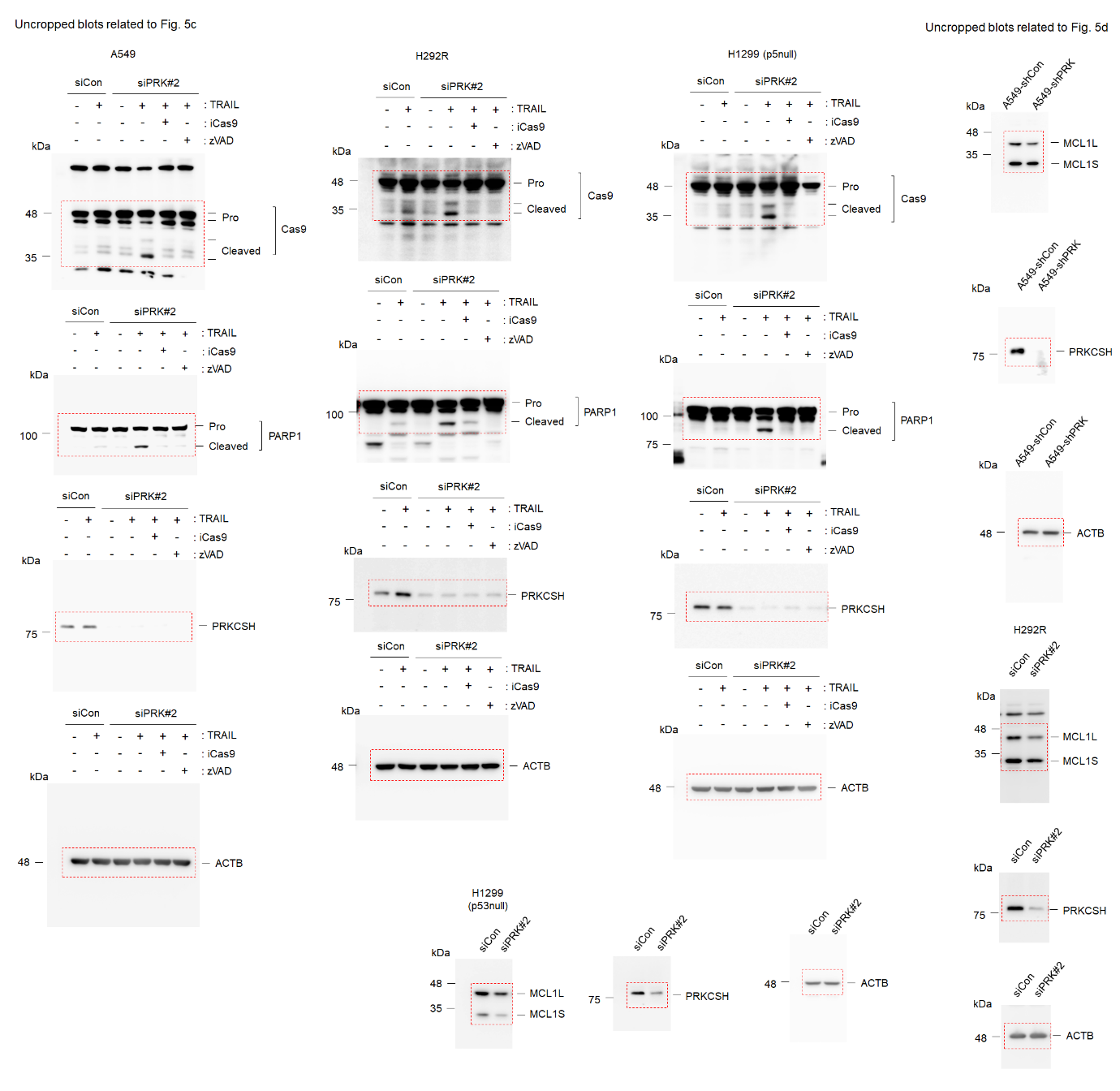

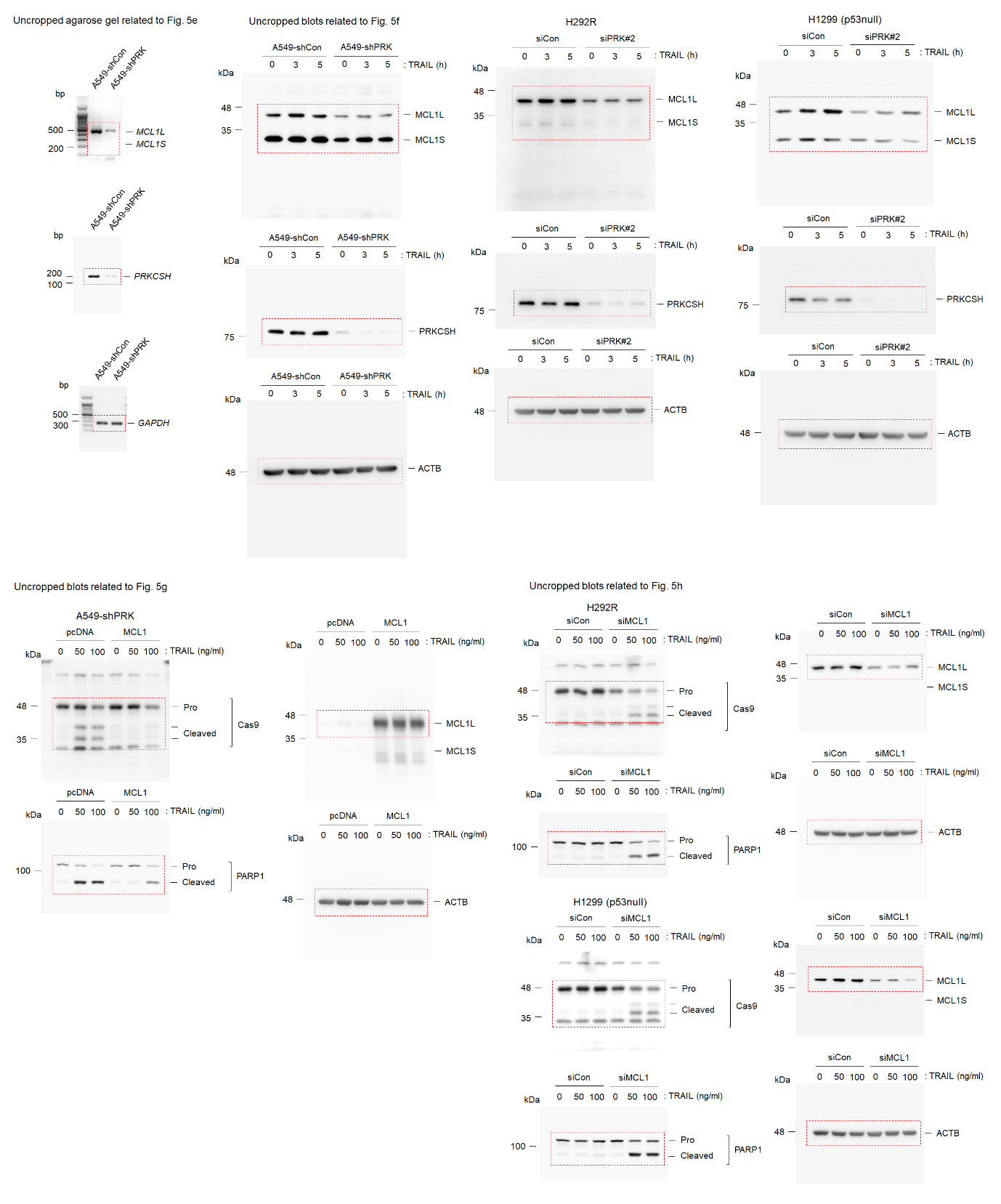

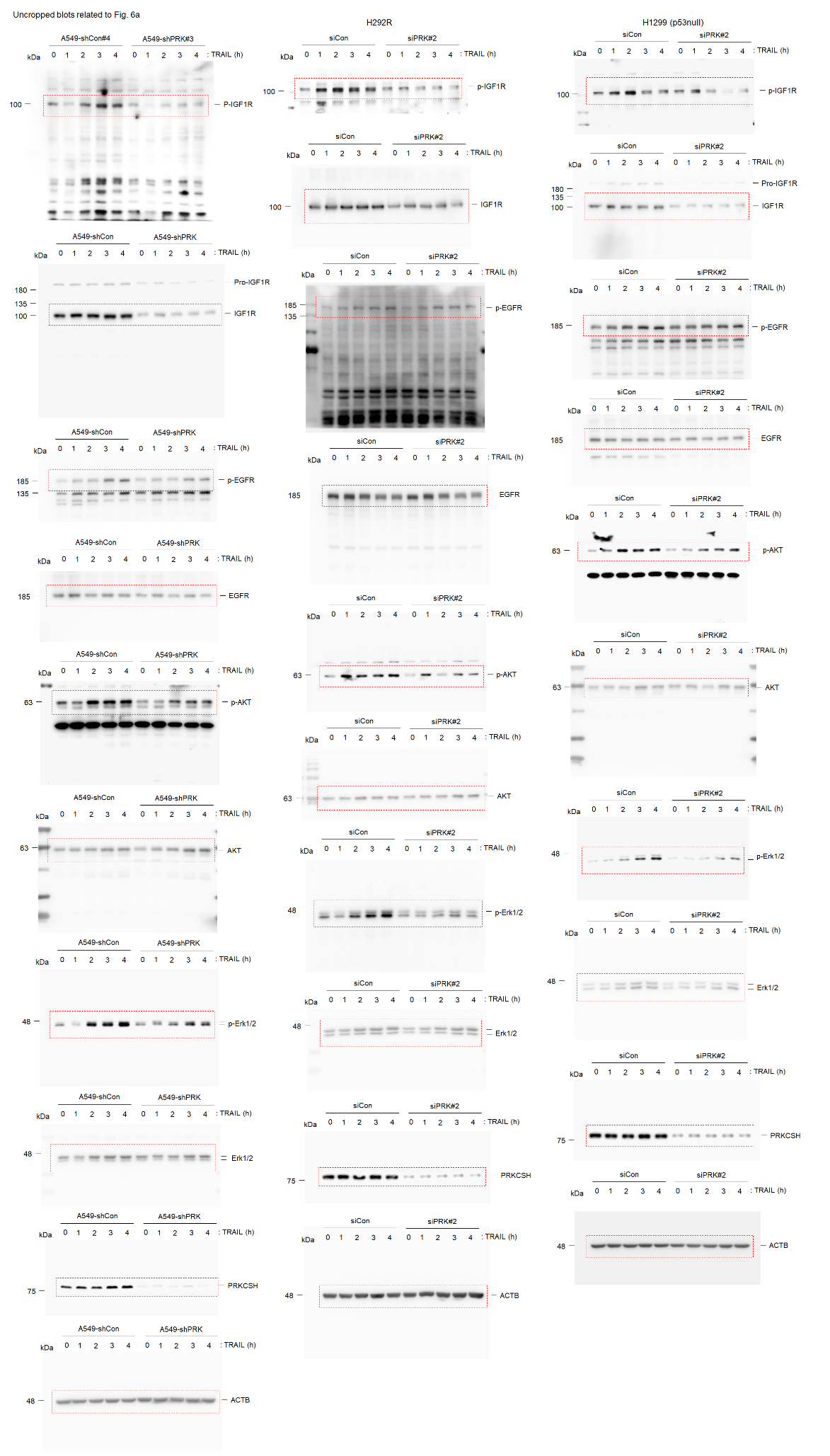

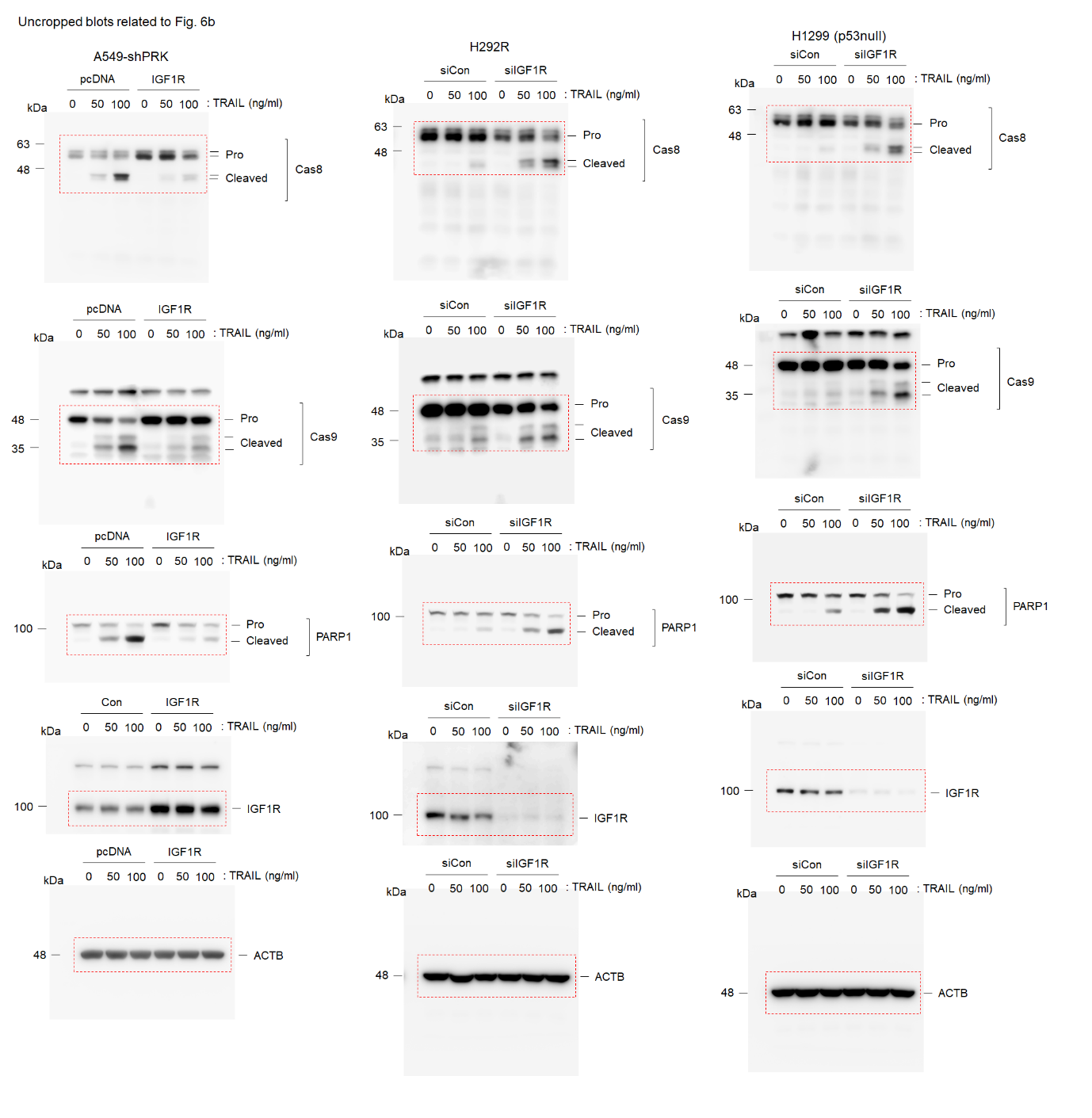

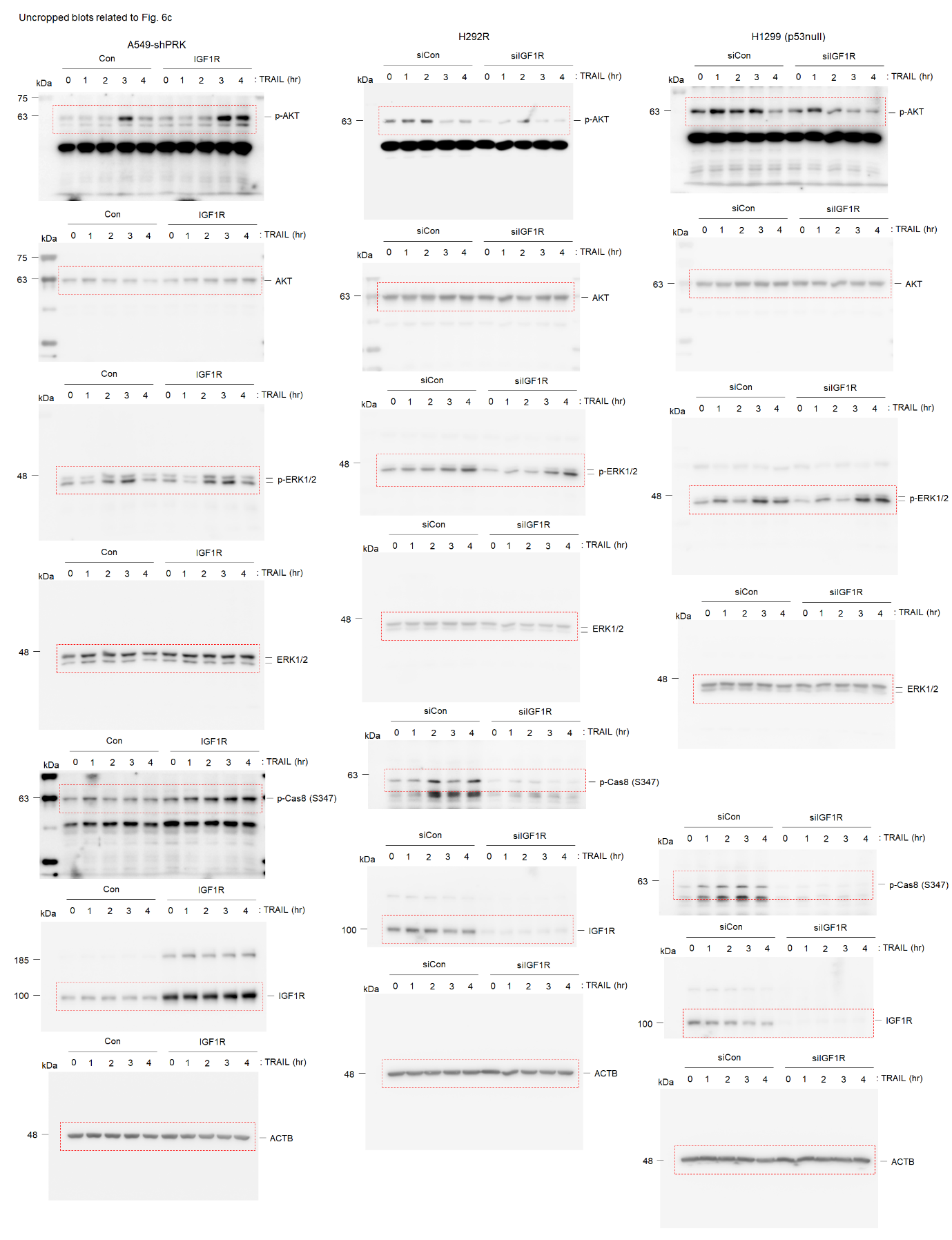

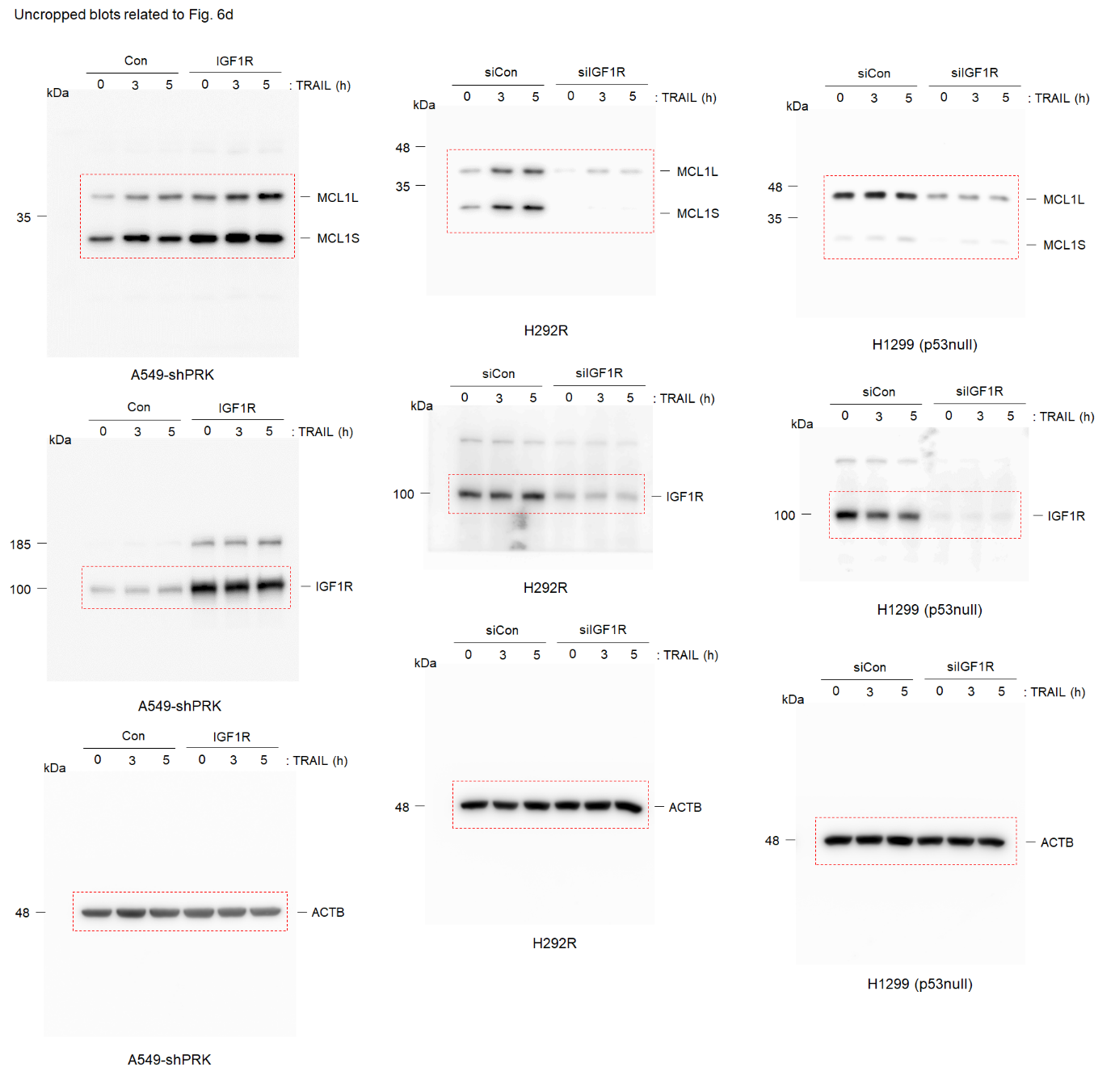

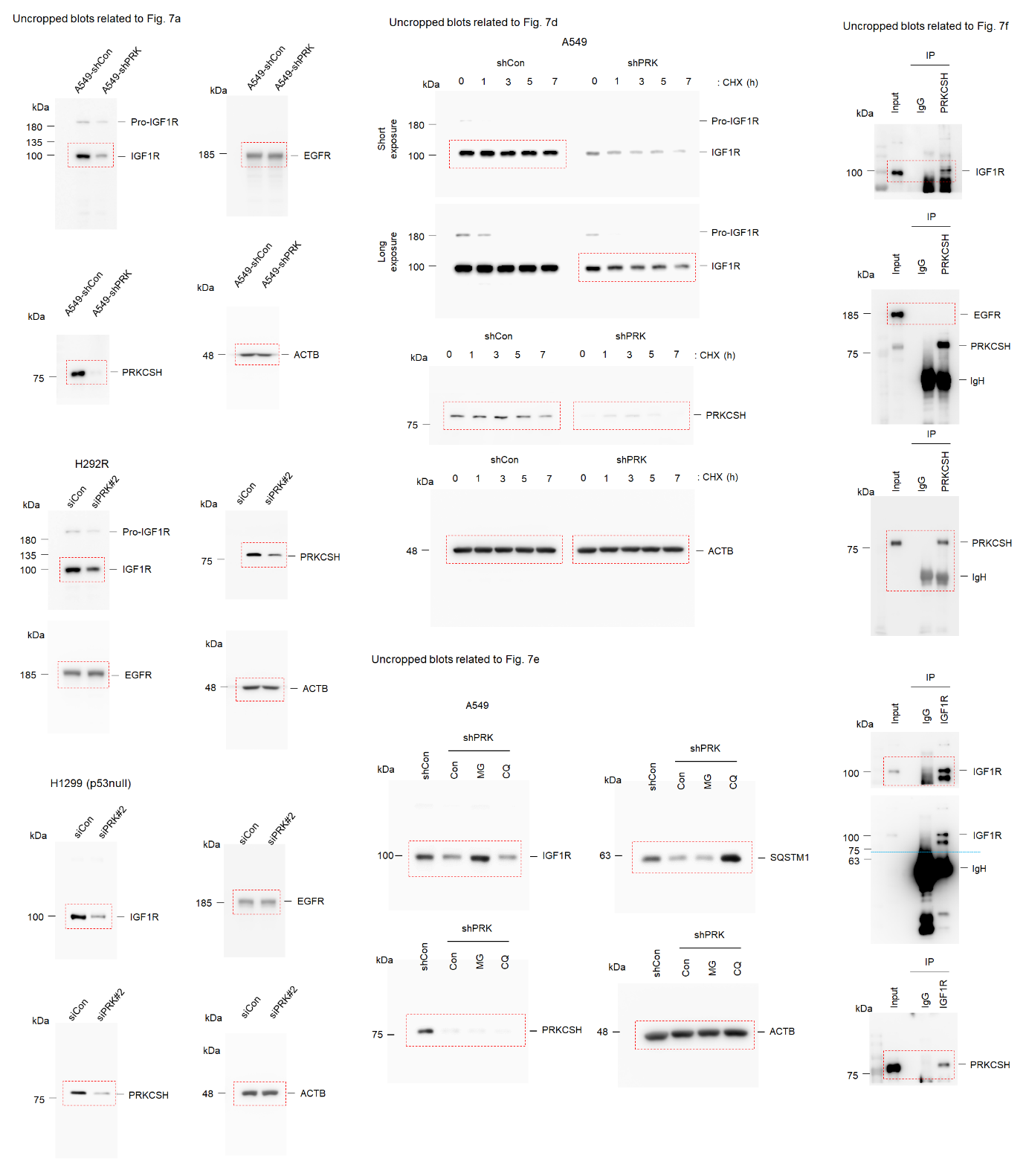


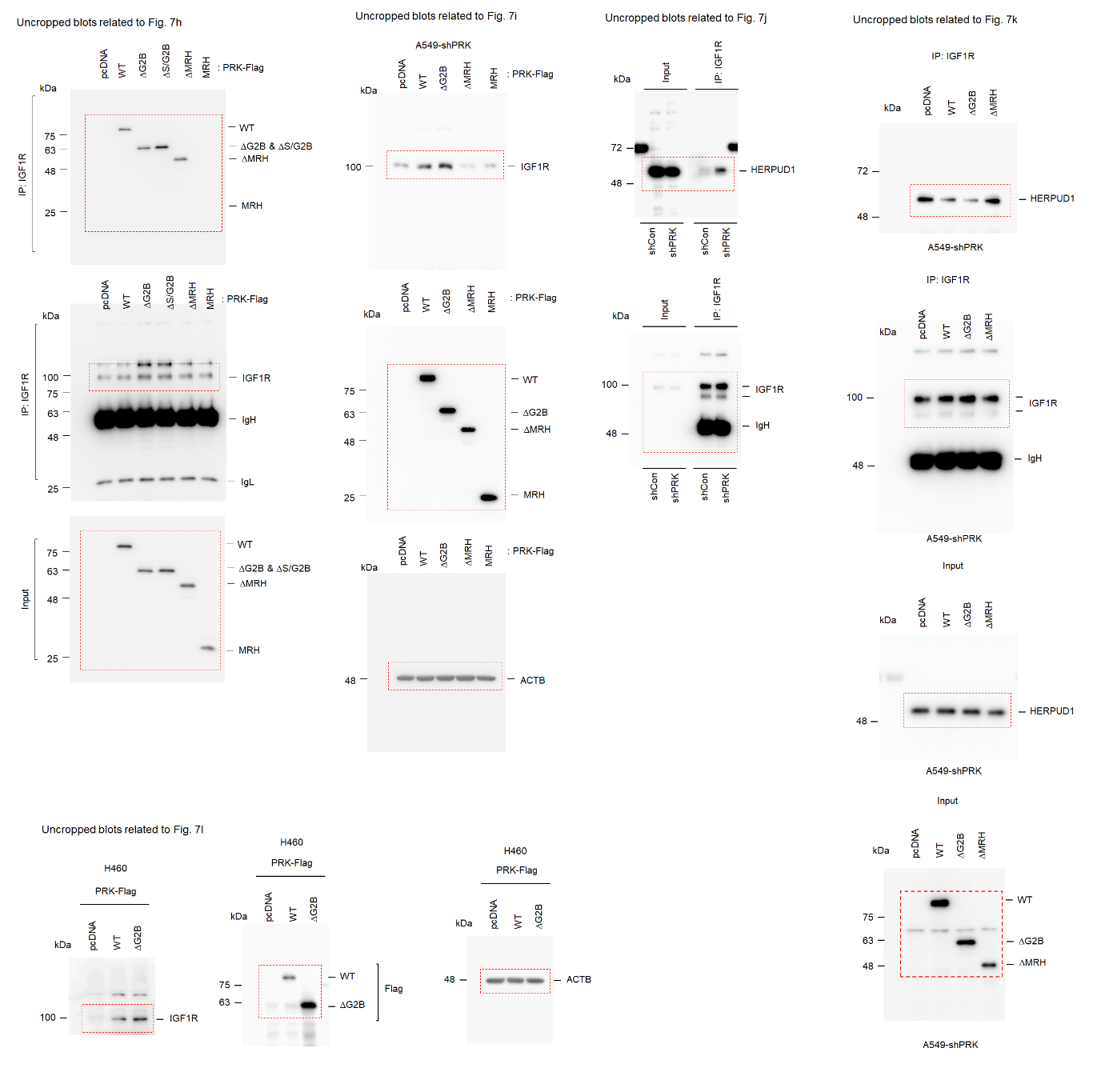


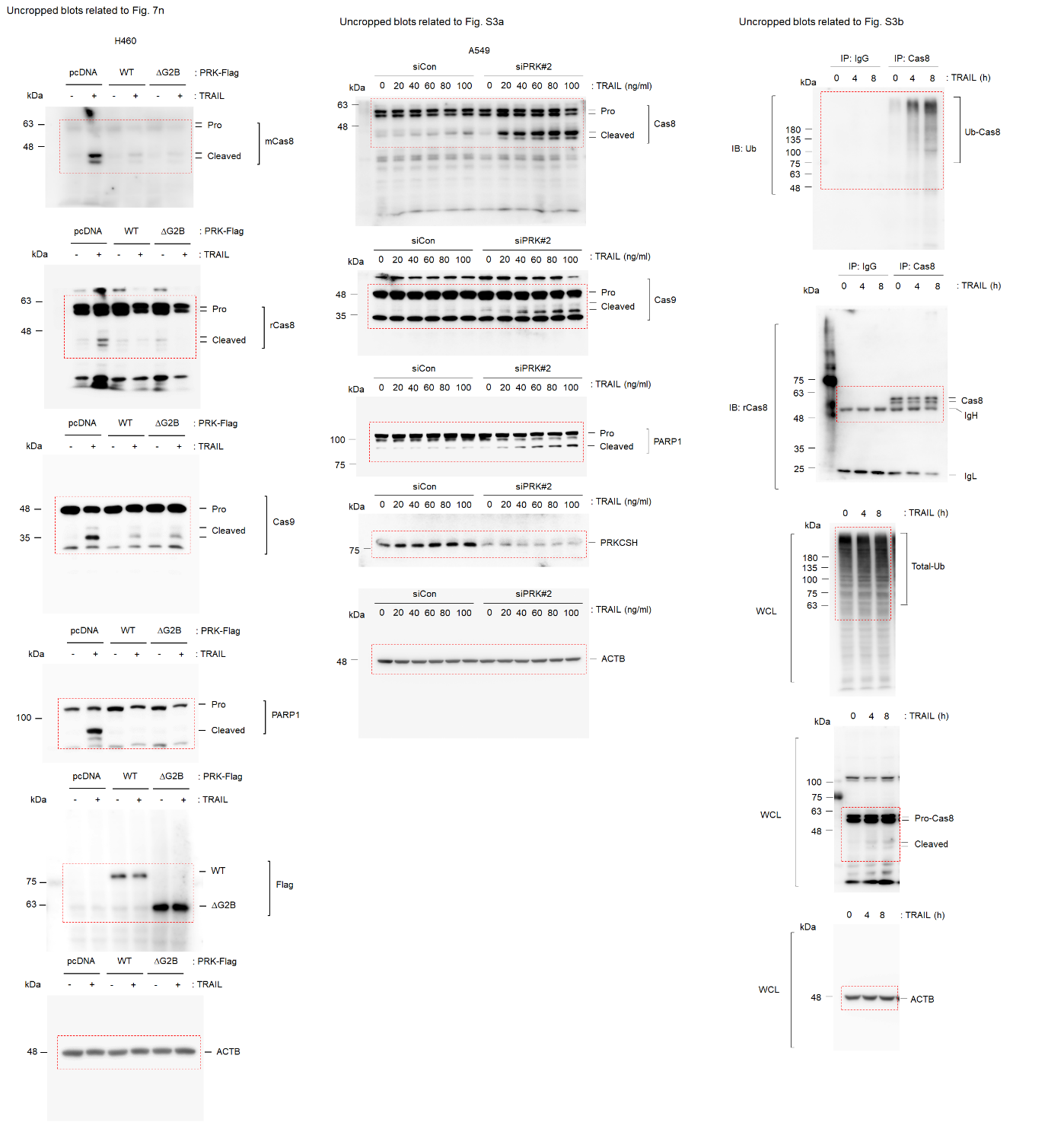


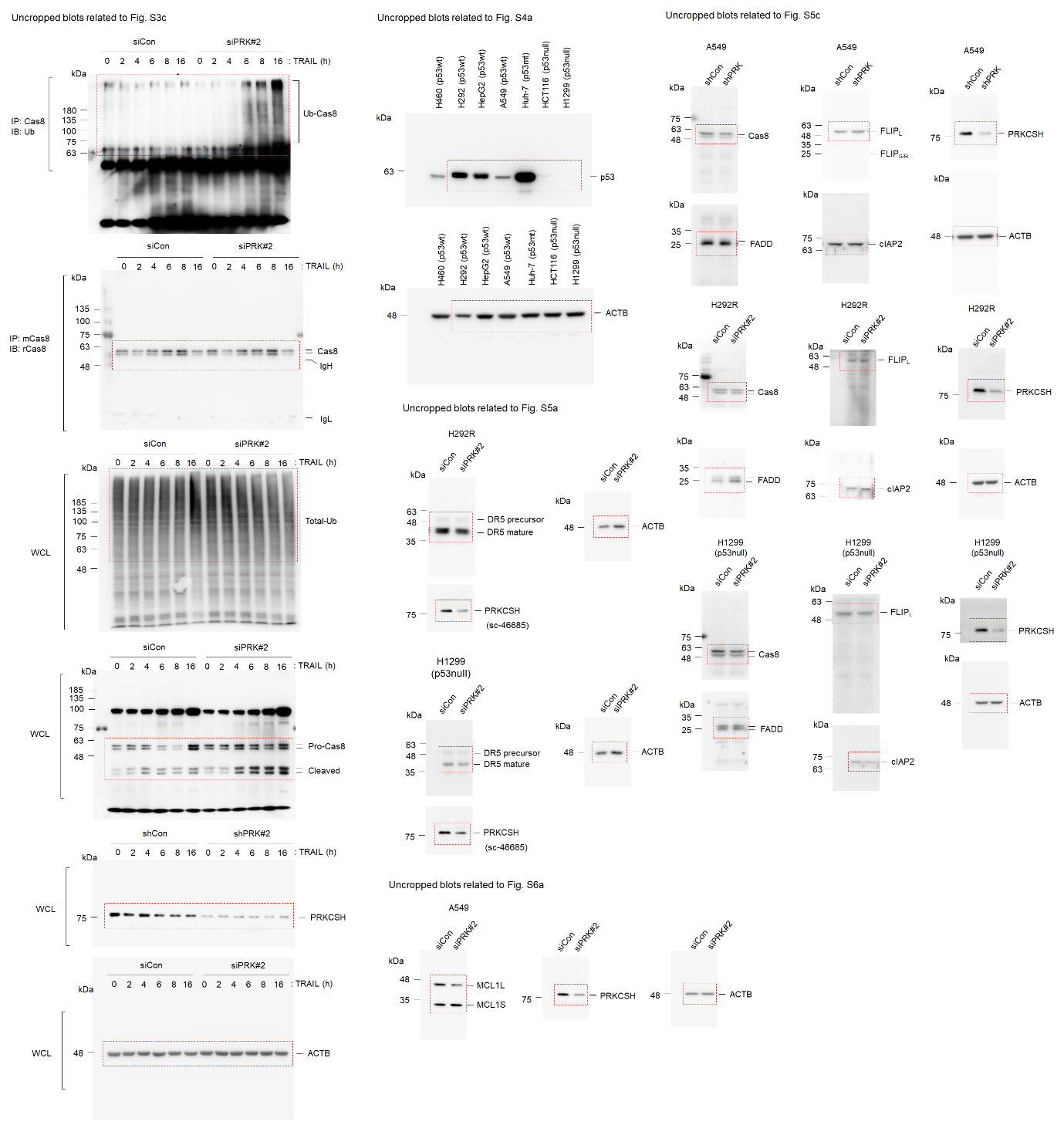

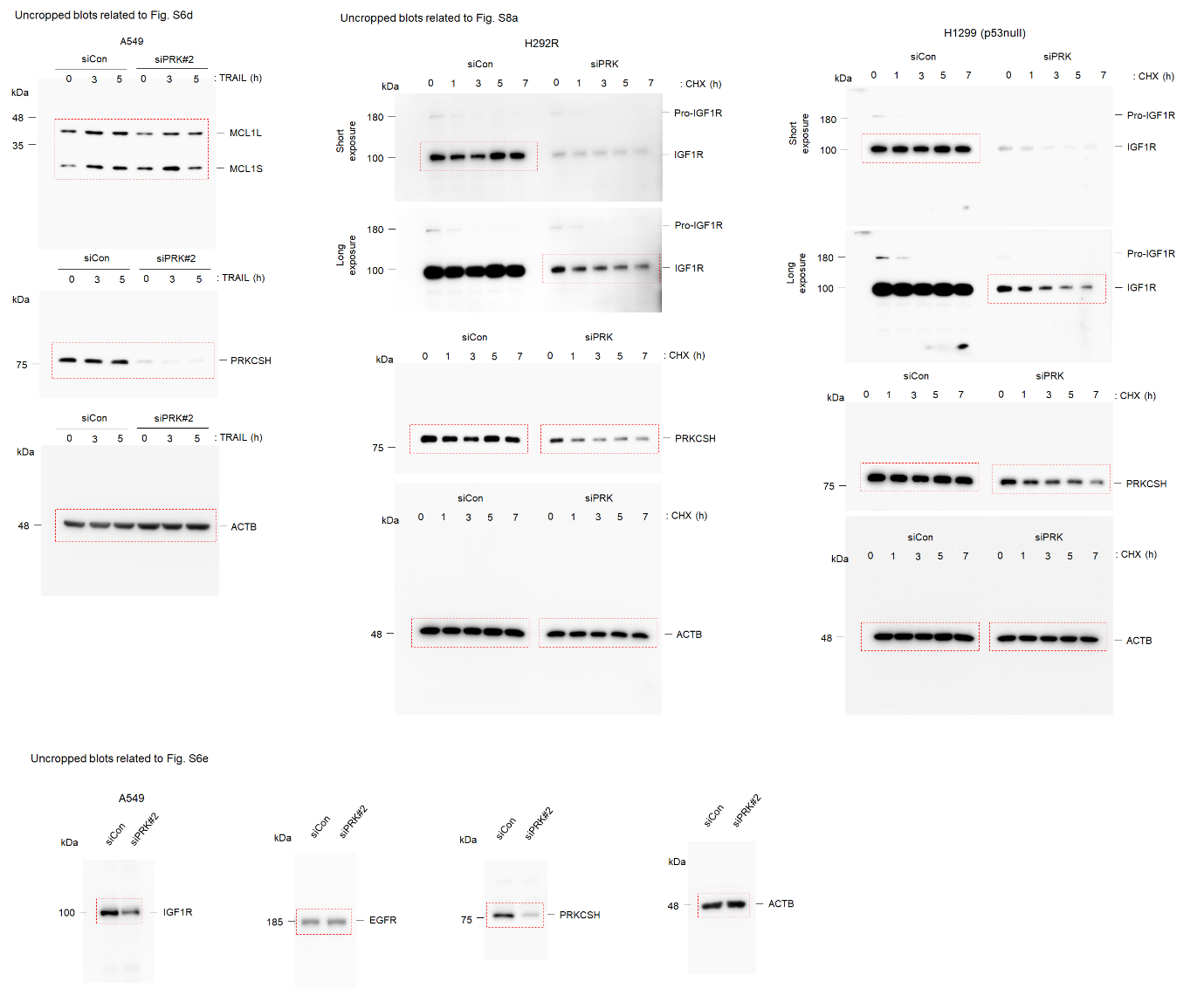


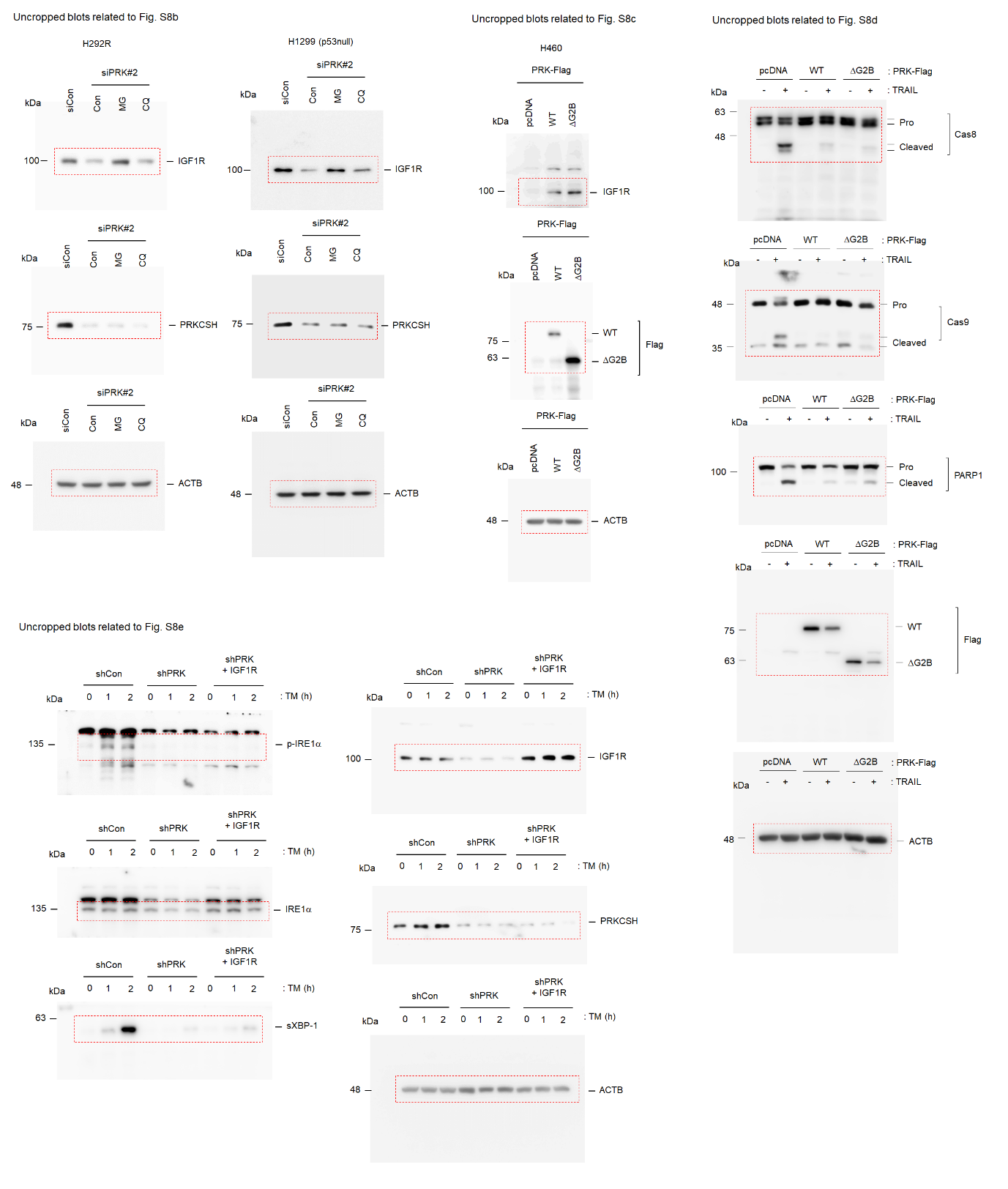

Supplement: Supplementary file 3 — Dataset 2 [file 12276_2023_1147_MOESM3_ESM.docx]
